# OTUD6B-mediated K48 deubiquitination of FXR1 forms a positive feedback loop activating MEK2/ERK signaling in colorectal cancer liver metastasis

**DOI:** 10.1038/s41419-026-08812-z

**Published:** 2026-04-29

**Authors:** Ying Lu, Ji Liu, Ya-Nan Li, Lin xiang, Guo-Bin Song, Tian Peng, Zhen Wang, Xue Yang, Hou-Qun Ying, Xue-Xin Cheng

**Affiliations:** 1 Jiangxi Province Key Laboratory of Immunology and Inflammation, Jiangxi Provincial Clinical Research Center for Laboratory Medicine, Department of Clinical Laboratory, The Second Affiliated Hospital, Jiangxi Medical College, Nanchang University, Nanchang, Jiangxi China; 2 Department of Transplantation, Jiangxi Provincial People’s Hospital, The First Affiliated Hospital of Nanchang Medical College, Nanchang, Jiangxi China; 3 Jiangxi Provincial Key Laboratory of Urinary System Diseases, Department of Urology, The First Affiliated Hospital, Jiangxi Medical College, Nanchang University, Nanchang, Jiangxi China

**Keywords:** Oncogenesis, Colorectal cancer

## Abstract

Liver metastasis remains a major cause of mortality in patients with colorectal cancer (CRC). Here, we identify OTUD6B, a deubiquitinating enzyme of the OTU family, as a key driver of CRC liver metastasis. OTUD6B is upregulated in metastatic CRC tissues and is correlated with poor prognosis. Functionally, OTUD6B promotes CRC cell proliferation, migration, and invasion in vitro and enhances tumor growth and liver metastasis in vivo. Mechanistically, OTUD6B binds the KH domain of fragile X-related protein 1 (FXR1) via its N-terminal region and stabilizes FXR1 by removing K48-linked polyubiquitin chains in a catalytic activity-dependent manner. In turn, FXR1 binds and stabilizes MEK2 mRNA, leading to increased MEK2 expression and activation of ERK signaling. Notably, FXR1 also upregulates OTUD6B expression, establishing a feed-forward loop that amplifies the oncogenic OTUD6B–FXR1–MEK2/ERK axis. OTUD6B, FXR1, and MEK2 levels are positively correlated in clinical CRC liver metastasis samples. Crucially, the MEK2 inhibitor U0126 acts synergistically with OTUD6B silencing to suppress liver metastasis. Collectively, these findings delineate a previously unrecognized oncogenic cascade in which OTUD6B stabilizes FXR1 to activate MEK2/ERK signaling, thereby driving CRC liver metastasis. Dual targeting of OTUD6B and MEK2 may represent a promising therapeutic strategy for advanced CRC.

## Introduction

Colorectal cancer (CRC) is the third most diagnosed malignancy worldwide and the second leading cause of cancer-related death [1]. The overall 5-year survival rate of patients with CRC is approximately 64%; however, the prognosis for those with metastatic CRC remains poor, with survival rates less than 15% [2]. The liver is the most frequent site of CRC metastasis, largely due to the direct drainage of mesenteric venous blood into the portal vein [3, 4]. As hepatic metastasis represents a primary driver of CRC-related mortality and poses a major clinical challenge, elucidating the mechanisms underlying CRC liver metastasis and identifying potential therapeutic targets are of substantial clinical importance.

Deubiquitinases (DUBs) are key components of the ubiquitin–proteasome system. By removing ubiquitin chains from target proteins, they prevent proteasomal degradation and play essential roles in maintaining cellular homeostasis and modulating diverse signaling pathways [5]. Among them, the ovarian tumor-related protease (OTU) family of DUBs has recently gained increasing attention for its involvement in tumor initiation, progression, and metastasis, as well as tumor microenvironment remodeling and immune evasion [6, 7]. Based on this, we previously explored the metastatic potential of OTU family members in clear cell renal cell carcinoma (ccRCC) [8], which further motivated us to investigate whether OTU DUBs may also drive metastatic progression in CRC. Nevertheless, the expression landscape, functional relevance, and mechanistic roles of OTU family DUBs in CRC remain incompletely characterized, particularly in metastatic settings. To address this gap, we performed unbiased transcriptomic profiling of OTU family genes in paired CRC and adjacent normal tissues. This analysis identified OTUD6B as one of the most prominently upregulated OTU family members in CRC, distinguishing it from other family members. Notably, elevated OTUD6B expression was strongly associated with adverse clinical outcomes, providing a compelling rationale for prioritizing OTUD6B in this study. OTUD6B has been reported to exert context-dependent roles in tumor biology: it displays tumor-suppressive functions in hepatocellular carcinoma [9] and esophageal squamous cell carcinoma [10], yet demonstrates oncogenic activity in cholangiocarcinoma [11], multiple myeloma [12] and breast cancer [13]. However, the molecular basis underlying its role in CRC liver metastasis—particularly with respect to its physiological substrates, ubiquitin-linkage specificity, and downstream signaling circuitry—remains insufficiently understood, representing a critical gap in our understanding of CRC metastatic progression.

Fragile X-related protein 1 (FXR1) is a pivotal RNA-binding protein (RBP) that broadly regulates posttranscriptional processes, including RNA splicing, subcellular localization, polyadenylation, translation, stability, and degradation [14]. As a canonical RBP, FXR1 has been increasingly recognized as a cancer-relevant regulator, and its dysregulation has been linked to malignant phenotypes such as enhanced proliferation, migration/invasion, and epithelial–mesenchymal transition, accompanied by reduced apoptosis [15, 16]. Elevated FXR1 expression has been reported in multiple malignancies, including head and neck squamous cell carcinoma, prostate cancer, and esophageal cancer, where it is strongly associated with poor prognosis [15, 17]. Nevertheless, the precise role of FXR1 in CRC initiation and progression—particularly in liver metastasis—remains to be fully elucidated.

The extracellular signal-regulated kinase (ERK) pathway is a highly conserved signaling cascade activated by diverse receptors [18]. Activating mutations frequently arise in core components such as RTKs, RAS, BRAF, CRAF, MEK1, and MEK2, converting these proteins into oncogenic drivers that sustain aberrant ERK activation and render tumor cells highly dependent on this pathway [19]. The ERK signaling axis plays a central role in tumor growth, invasion, metastasis, extracellular matrix remodeling, and angiogenesis [20, 21]. Notably, increasing evidence indicates that dysregulated activation of the MEK–ERK cascade promotes CRC proliferation and metastasis [22, 23].

In this study, we demonstrated that OTUD6B is markedly upregulated in CRC, and that its silencing suppresses CRC proliferation and liver metastasis both in vitro and in vivo. Mechanistically, OTUD6B stabilizes FXR1 protein expression through its deubiquitinating activity. FXR1, in turn, binds to and stabilizes MEK2 mRNA, thereby increasing MEK2 protein expression and activating the ERK signaling pathway. Moreover, FXR1 upregulates OTUD6B expression, forming a positive feedback loop that amplifies the oncogenic effect of the OTUD6B/FXR1/MEK2/ERK axis. Notably, the MEK2 inhibitor U0126 synergizes with OTUD6B silencing to further inhibit CRC liver metastasis. Collectively, these findings highlight the OTUD6B/FXR1/MEK2/ERK axis as a promising therapeutic target for CRC liver metastasis.

## Results

### OTUD6B overexpression is associated with adverse prognosis and metastasis progression in CRC

**Fig. 1:**
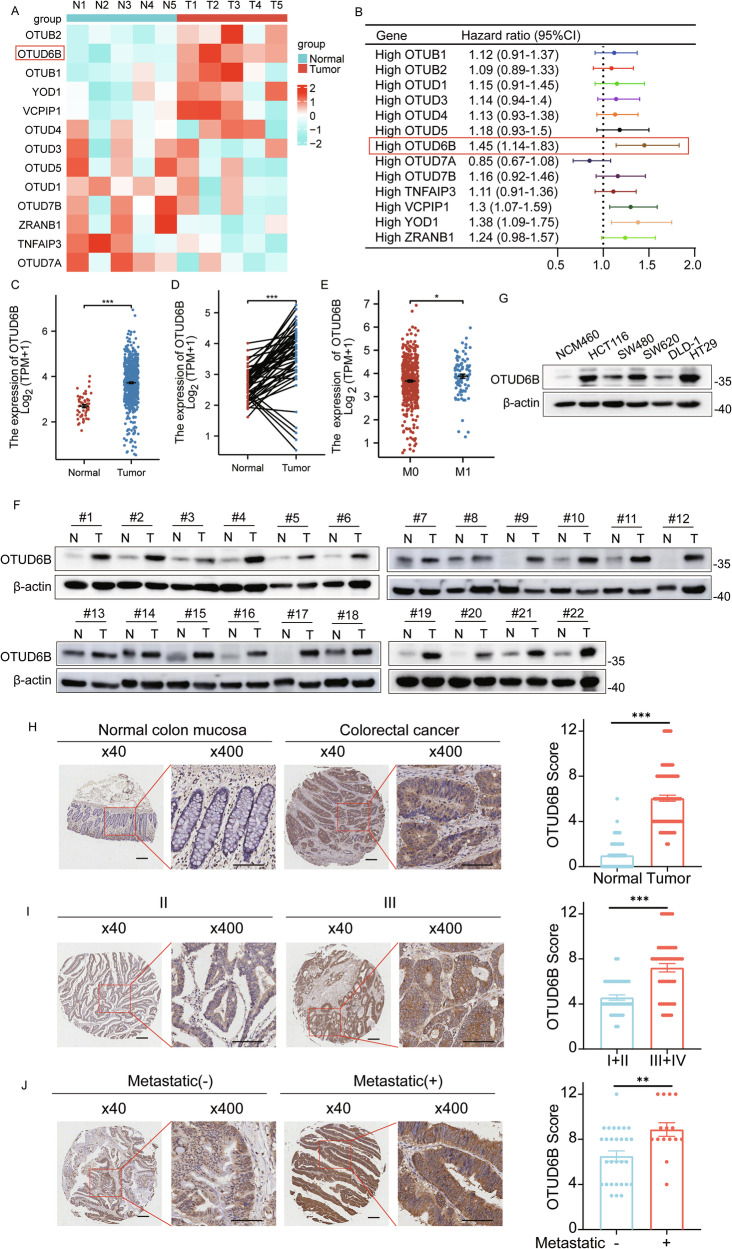
OTUD6B overexpression is associated with adverse prognosis and metastasis progression in CRC. **A** Transcriptomic profiling of OTU family genes in CRC as determined by whole-transcriptome sequencing. **B** Forest plot of overall survival-associated hazard ratios (HRs) for multiple OTU family genes in CRC, analyzed using the Kaplan–Meier Plotter database. **C** OTUD6B mRNA expression in CRC tissues versus normal colorectal epithelium, based on TCGA data. **D** Paired comparison of OTUD6B mRNA levels between CRC and matched adjacent normal tissues within the TCGA cohort. **E** OTUD6B expression in primary tumors from patients with and without distant metastases (TCGA). **F** Western blot analysis of OTUD6B protein levels in CRC and adjacent normal tissues, N = normal, T = tumor. **G** Differential OTUD6B protein expression in normal colonic epithelial cells and five CRC cell lines. **H** Representative immunohistochemical staining of OTUD6B in CRC and paired adjacent tissues (left), and corresponding quantification (right). Scale bars: ×40 = 200 μm; ×400 = 100 μm. **I** IHC analysis of OTUD6B expression in early-stage (I–II) versus late-stage (III–IV) CRC tissues. **J** IHC analysis of OTUD6B expression in patients with CRC without (stage I–III) and with liver metastases (stage IV). Statistical significance was determined using two-tailed Student’s *t* tests for two-group comparisons. *p < 0.05; **p < 0.01; ***p < 0.001.

To characterize the expression profiles of OTU family genes in CRC, we collected five pairs of fresh tumor and adjacent normal tissue samples and performed whole-transcriptome sequencing. Among the 13 OTU family members, only OTUB2, OTUD6B, and OTUD7A displayed significant differential expression, with the expression of OTUB2 and OTUD6B markedly upregulated and that of OTUD7A significantly downregulated (Fig. 1A, Table S1). To evaluate the prognostic significance of OTU family DUBs in CRC, an analysis based on the Kaplan–Meier Plotter database was conducted. High expression levels of OTUD6B, VCPIP1, and YOD1 were significantly associated with poor overall survival, with OTUD6B exhibiting the highest hazard ratio (Fig. 1B and Figure S1), suggesting a strong correlation with adverse clinical outcomes. Integration of these transcriptomic data with prognostic analyses revealed that OTUD6B is a candidate of CRC pathogenesis. To further characterize OTUD6B expression in CRC, RNA sequencing data from 647 patients with CRC in the TCGA were analyzed. OTUD6B mRNA levels were significantly higher in tumor tissues than in adjacent normal tissues (Fig. 1C). This pattern was consistently observed in 50 matched CRC–normal tissue pairs within the same cohort (Fig. 1D). Furthermore, OTUD6B expression was higher in primary tumors from patients with distant metastasis than in those without (Fig. 1E), indicating its potential role in tumor dissemination.

Western blot analysis of 22 paired CRC samples confirmed significantly elevated OTUD6B expression in tumor tissues compared with matched normal tissues (Fig. 1F). Similarly, compared with the normal colonic epithelial cell line NCM460, five CRC cell lines (HCT116, SW480, SW620, DLD-1, and HT29) presented increased OTUD6B protein levels (Fig. 1G). To validate the association between OTUD6B expression and CRC progression, immunohistochemistry was performed on tissue microarrays containing 90 paired CRC and adjacent normal samples. The OTUD6B protein was markedly overexpressed in tumor tissues (Fig. 1H). Notably, OTUD6B levels were significantly higher in patients with lymph node metastasis (stage III–IV) than in those without nodal involvement (stage I–II) (Fig. 1I) and in patients with distant metastasis (stage IV) than in nonmetastatic patients (stage I–III) (Fig. 1J). These findings support a strong link between OTUD6B overexpression and aggressive, metastatic CRC phenotypes.

### OTUD6B promotes CRC growth and metastasis in vitro and in vivo

**Fig. 2:**
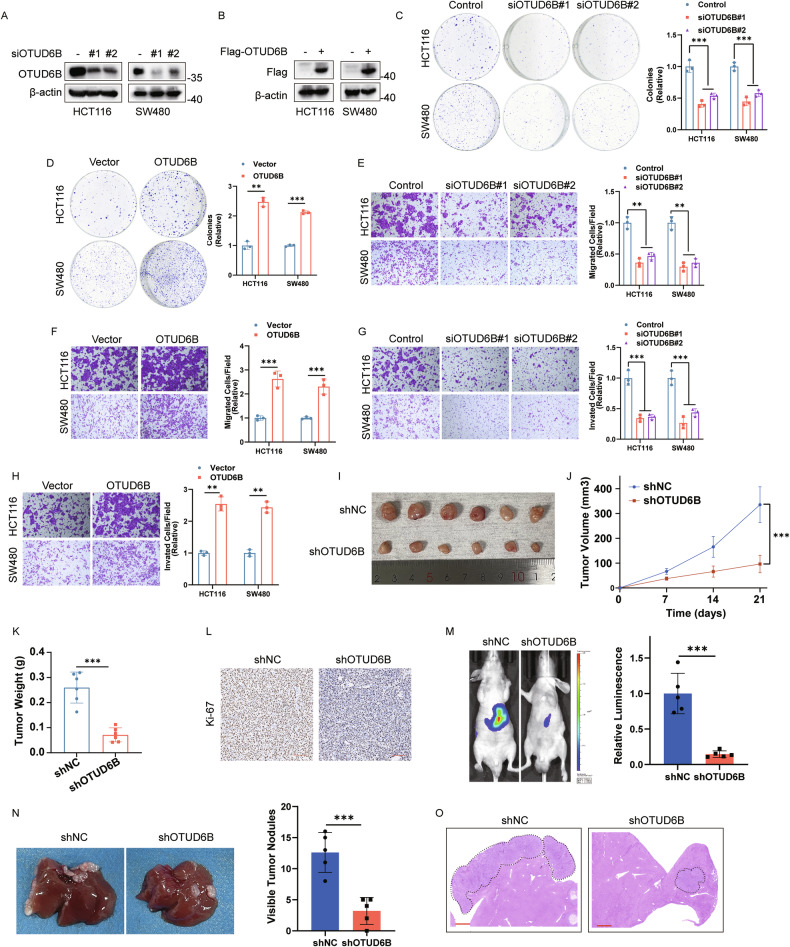
OTUD6B promotes CRC growth and metastasis in vitro and in vivo. **A**, **B** OTUD6B was silenced or overexpressed in HCT116 and SW480 cells using OTUD6B-specific siRNAs or overexpression plasmids, respectively; **C**, **D** Representative images and quantification of colony formation assays following OTUD6B knockdown (**C**) or overexpression (**D**); **E–H** Transwell assays were used to assess the effects of OTUD6B knockdown or overexpression on cell migration (**E**, **F**) and invasion (**G**, **H**). **I–L** In vivo tumorigenesis was evaluated by subcutaneous injection of shNC or shOTUD6B HCT116 cells into nude mice. Tumor morphology (**I**), growth curves (**J**), tumor weights (**K**), and Ki-67-positive rates (**L**) were compared; scale bar: 100 μm; **M–O** Liver metastases were analyzed in the splenic injection models. Representative in vivo fluorescence images and quantification of hepatic metastases (**M**), macroscopic liver surface nodules (**N**), and histological evaluation of metastatic lesions (**O**) are shown; scale bar: 1 mm. Statistical significance was determined using two-tailed Student’s *t* tests and ANOVA. **p < 0.01; ***p < 0.001.

Given its association with metastatic CRC, we next examined the functional impact of OTUD6B on the CRC cell metastatic process. First, cells were transfected with OTUD6B-specific siRNAs or an OTUD6B overexpression plasmid to establish knockdown and overexpression models, respectively (Fig. 2A–B). In vitro functional assays demonstrated that OTUD6B knockdown significantly suppressed CRC cell proliferation, migration, and invasion (Fig. 2C, E, G), whereas OTUD6B overexpression markedly enhanced these malignant phenotypes (Fig. 2D, F, H). To further assess its oncogenic role in vivo, HCT116 cells with stable OTUD6B knockdown were established (Fig. S2A) and implanted into BALB/c nude mice to generate subcutaneous xenograft and splenic injection liver metastasis models. OTUD6B-deficient tumors exhibited significantly slower growth and lower tumor weight compared with controls (Fig. 2I–K). Immunohistochemical analysis of Ki-67 confirmed markedly decreased proliferative activity in tumors lacking OTUD6B (Fig. 2L). In the liver metastasis model, silencing OTUD6B led to a pronounced reduction in hepatic metastatic burden, as evidenced by diminished fluorescence signal intensity (Fig. 2M), fewer visible nodules on the liver surface (Fig. 2N), and a significantly lower number of histologically confirmed metastatic foci (Fig. 2O). Collectively, these findings indicate that OTUD6B promotes CRC cell proliferation, migration, invasion, and liver metastasis both in vitro and in vivo.

### OTUD6B specifically interacts with FXR1 via its N-terminal region and the KH domain of FXR1

**Fig. 3:**
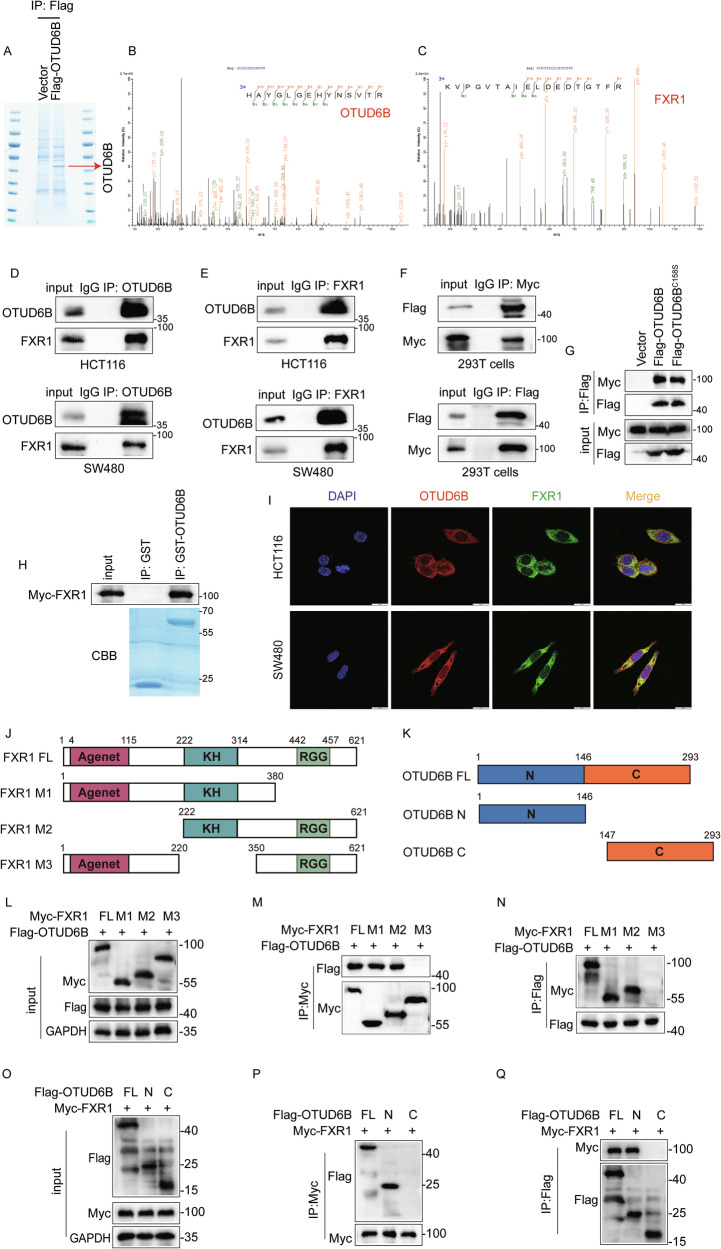
OTUD6B specifically interacts with FXR1 via the KH domain of FXR1 and the N-terminal region of OTUD6B. **A** HEK-293T cells were transfected with Flag-tagged OTUD6B or empty vector. OTUD6B-interacting proteins were separated and visualized by co-IP followed by Coomassie blue staining. **B**, **C** LC–MS/MS analysis revealed specific peptides of OTUD6B (**B**) and FXR1 (**C**) detected in the Flag-OTUD6B IP complex; **D**, **E** Co-IP using anti-OTUD6B antibody (**D**) or anti-FXR1 antibody (**E**) was performed in HCT116 and SW480 cells to validate endogenous OTUD6B-FXR1 interaction, with IgG as a negative control; **F** Co-IP using anti-Myc antibody or anti-FXR1 antibody was performed in HEK-293T cells ectopically coexpressing Flag-OTUD6B and Myc-FXR1. **G** Flag-tagged OTUD6B or its catalytic mutant OTUD6B^C158S^ was coexpressed with Myc-FXR1 in HEK-293T cells, and co-IP was performed using anti-Flag antibody to assess interaction. **H** Purified GST or GST-OTUD6B protein was incubated with lysates from Myc-FXR1-transfected HEK-293T cells, followed by pull-down with GSH beads and detection using anti-Myc antibody; GST-OTUD6B protein purity was assessed by Coomassie staining (CBB). **I** Immunofluorescence staining was performed using anti-OTUD6B and anti-FXR1 antibodies in HCT116 and SW480 cells, scale bar: 20 μm. **J**, **K** Schematic diagrams of Myc-tagged FXR1 (**J**) and Flag-tagged OTUD6B (K) truncation mutants. **L** Expression of FXR1 full-length and truncation mutants was confirmed following co-transfection with Flag-OTUD6B in HEK-293T cells; **M**, **N** Myc-FXR1 full-length or truncation constructs were co-expressed with Flag-OTUD6B in HEK-293T cells, and co-IP was conducted using anti-Myc antibody (**M**) or anti-Flag antibody (**N**) to detect interactions. **O** Expression of OTUD6B full-length and truncation mutants was confirmed following co-transfection with Myc-FXR1 in HEK-293T cells. **P**, **Q** Flag-OTUD6B full-length or truncation mutants were co-expressed with Myc-FXR1 in HEK-293T cells, and co-IP was conducted using anti-Myc antibody (**P**) or anti-Flag antibody (**Q**) to analyze protein interaction.

Given that OTUD6B is a DUB that regulates protein stability or activity by removing substrate ubiquitination, we sought to identify its potential interacting substrates in cells. HEK-293T cells were transfected with Flag-tagged OTUD6B or empty vector, followed by co-IP, SDS–PAGE, and Coomassie blue staining. Specific gel bands were excised and subjected to LC–MS/MS analysis (Fig. 3A). To ensure high-confidence identification, the LC-MS/MS output was first filtered based on the following stringent criteria: (1) proteins exclusively detected in the Flag-OTUD6B group but absent in the Flag-Vector control; (2) protein FDR confidence classified as “High”; and (3) a sum PEP score ≥ 5, indicating robust peptide evidence. The filtered candidates were subsequently subjected to Gene Ontology (GO) enrichment analysis, which revealed significant overrepresentation of biological processes associated with RNA metabolism and post-transcriptional regulation—most notably RNA splicing and mRNA processing (Fig. S2B, C). Key genes mapping to these pathways included RBM4B, HNRNPU, RBM14, SRSF3, FXR1, and TIA1. To further refine the most biologically relevant substrate, we examined the expression of these top GO-enriched candidates following OTUD6B knockdown in HCT116 cells. Strikingly, only FXR1 exhibited a robust reduction upon OTUD6B depletion, while the other candidates remained largely unchanged (Fig. 3B, C and Fig. S2D). Combined with its well-documented roles in tumor metastasis and EMT processes, these results strongly supported FXR1 as the most physiologically relevant OTUD6B-interacting substrate in the context of CRC metastasis. Co-IP assays using OTUD6B or FXR1 antibodies in HCT116 and SW480 cells confirmed this interaction (Fig. 3D, E). Coexpression of Flag-OTUD6B and Myc-FXR1 in HEK-293T cells resulted in strong binding (Fig. 3F). Furthermore, a catalytically inactive mutant (OTUD6B^C158S^) maintained its interaction with FXR1, suggesting that the binding does not depend on OTUD6B enzymatic activity (Fig. 3G). A glutathione S-transferase (GST) pull-down assay using purified GST-OTUD6B protein further validated the direct interaction between OTUD6B and FXR1 in vitro (Fig. 3H). Furthermore, immunofluorescence staining revealed prominent cytoplasmic colocalization of endogenous OTUD6B and FXR1 in HCT116 and SW480 cells (Fig. 3I). To define the interaction domains, we generated Myc-tagged FXR1 truncation mutants (Fig. 3J) and Flag-tagged OTUD6B truncation mutants (Fig. 3K). Co-IP experiments demonstrated that OTUD6B primarily interacted with the KH domain of FXR1 (Fig. 3L–N), whereas FXR1 predominantly bound to the N-terminal region of OTUD6B (Fig. 3O–Q). These findings identify FXR1 as a specific interactor of OTUD6B, mediated by the KH domain of FXR1 and the N-terminal region of OTUD6B.

### OTUD6B enhances FXR1 protein stability and promotes CRC progression

**Fig. 4:**
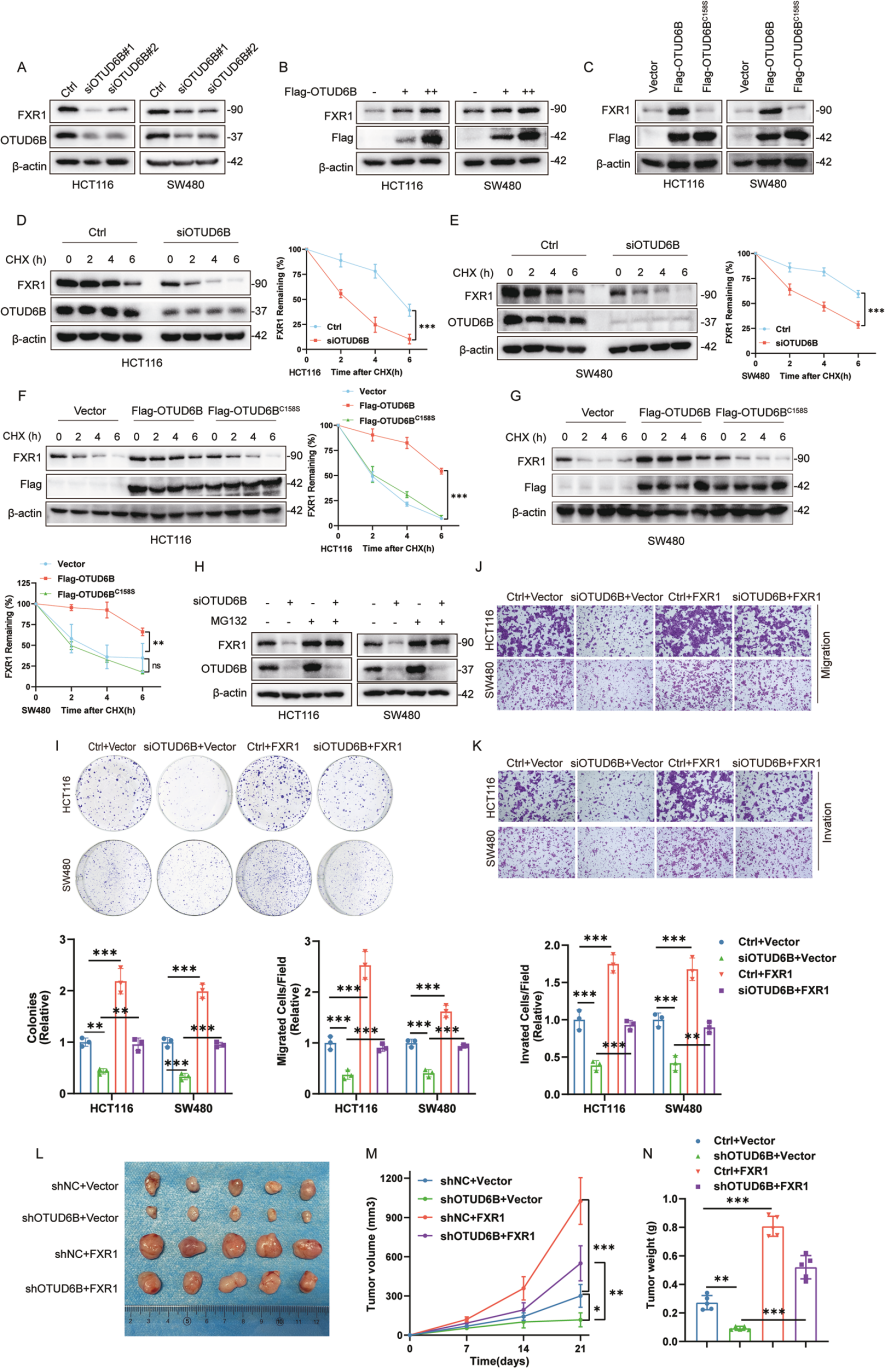
OTUD6B stabilizes the FXR1 protein and promotes CRC cell growth and metastasis. **A**, **B** Detection of FXR1 protein levels after OTUD6B knockdown (**A**) or OTUD6B overexpression (+: 1 μg Flag-OTUD6B plasmid; ++: 3 μg Flag-OTUD6B plasmid) (**B**) in CRC cells; **C** Detection of FXR1 protein in CRC cells ectopically expressing wild-type OTUD6B or the catalytically inactive mutant OTUD6B^C158S^. **D**, **E** Cells were treated with cycloheximide (CHX, 20 μM) after OTUD6B knockdown, and FXR1 degradation was monitored at the indicated time points by Western blotting in HCT116 (**D**) and SW480 (**E**) cells; **F**, **G** Cells were transfected with Flag-OTUD6B or Flag-OTUD6B^C158S^ followed by CHX treatment (20 μM), and FXR1 stability was assessed over time in HCT116 (**F**) and SW480 (**G**) cells. **H** Detection of FXR1 protein in CRC cells after OTUD6B knockdown, with or without treatment with MG132 (25 μM, 6 h); **I–K** Cells were transfected with OTUD6B siRNAs or FXR1 plasmids (with or without cotransfection of OTUD6B siRNAs), followed by colony formation assays (**I**) and Transwell migration (**J**) and invasion assays (**K**). **L–N** In vivo tumorigenesis was evaluated by subcutaneous injection of indicated cells into nude mice. Tumor morphology (**L**), growth curves (**M**) and tumor weights (**N**) were compared. Statistical significance was determined using ANOVA. ns = No significant difference; *p < 0.05; **p < 0.01; ***p < 0.001.

Given the established interaction between OTUD6B and FXR1, we next investigated whether OTUD6B regulates FXR1 protein stability. FXR1 protein levels significantly decreased in response to OTUD6B knockdown (Fig. 4A), whereas OTUD6B overexpression led to a marked and dose-dependent increase in FXR1 expression (Fig. 4B). In contrast, the catalytically inactive OTUD6B^C158S^ mutant failed to alter FXR1 levels, indicating that the stabilizing effect was dependent on the deubiquitinase activity of OTUD6B (Fig. 4C). Cycloheximide chase assays revealed that silencing OTUD6B accelerated FXR1 protein degradation (Fig. 4D, E), whereas OTUD6B overexpression markedly prolonged the half-life of FXR1, and no effect was observed with the OTUD6B^C158S^ mutant (Fig. 4F, G). Furthermore, proteasome inhibition with MG132 reversed the decrease in the expression of FXR1 induced by OTUD6B knockdown, suggesting that OTUD6B maintains the stability of FXR1 by preventing its proteasomal degradation (Fig. 4H). Functionally, FXR1 overexpression reversed the suppressive effects of OTUD6B depletion on cell proliferation, migration, invasion and tumor growth (Fig. 4I–N). Importantly, these results were recapitulated in the KRAS wild-type CRC cell line HT29, in which OTUD6B knockdown significantly suppressed, whereas FXR1 overexpression effectively rescued, these malignant phenotypes (Fig. S3A–C). We further assessed the expression of key EMT markers (E-cadherin and N-cadherin) and metastasis-associated markers (MMP9 and TIMP1) in tumor tissues from subcutaneous xenograft models. OTUD6B depletion significantly increased E-cadherin and TIMP1 levels while reducing N-cadherin and MMP9 expression, indicating a suppression of EMT and metastatic potential; notably, FXR1 overexpression reversed these alterations (Fig. S3D). Collectively, these data indicate that OTUD6B stabilizes FXR1 and promotes CRC cell proliferation, EMT, and metastatic traits in an FXR1-dependent manner.

### OTUD6B stabilizes FXR1 by removing K48-linked polyubiquitin chains in a catalytic activity-dependent manner

**Fig. 5:**
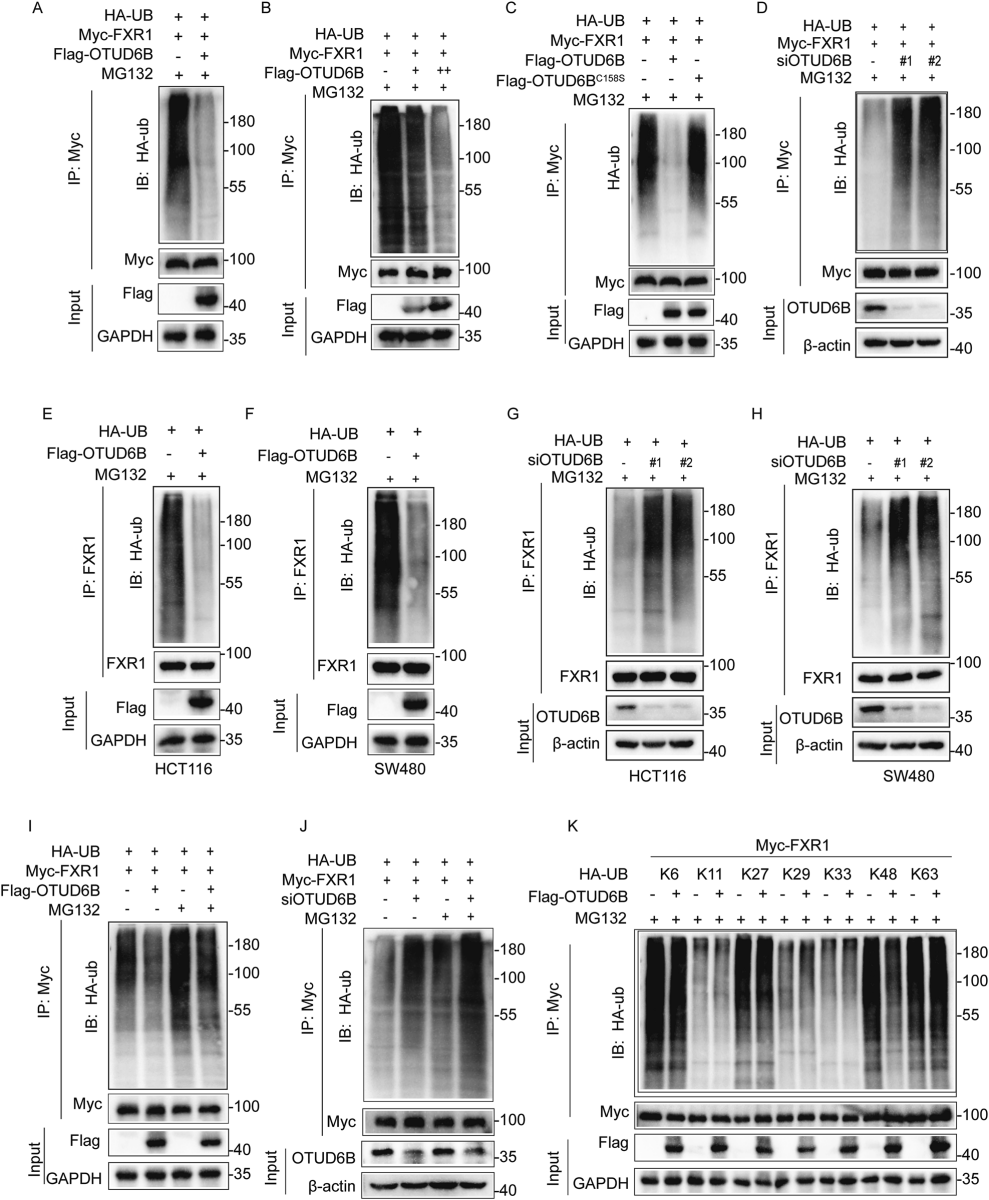
OTUD6B removes K48-linked polyubiquitin chains from FXR1 in a catalytic activity-dependent manner. **A** HA-UB, Myc-FXR1, and Flag-OTUD6B were ectopically expressed in HEK-293T cells, which were then treated with 25 μM MG132 for 6 h. FXR1 ubiquitination was detected by co-IP using an anti-Myc antibody. **B** HA-UB and Myc-FXR1 were cotransfected with increasing doses of Flag-OTUD6B ( + : 1 μg, ++: 3 μg) into HEK-293T cells. FXR1 ubiquitination was analyzed to assess dose dependency. **C** HA-UB and Myc-FXR1 were cotransfected with wild-type OTUD6B or a catalytically inactive mutant OTUD6B^C158S^, followed by treatment with MG132. FXR1 ubiquitination was evaluated as described in (**A**). **D** HA-UB, Myc-FXR1, and siOTUD6B were cotransfected into HEK-293T cells, which were analyzed by co-IP and immunoblotting. **E**, **F** HA-UB and Flag-OTUD6B were ectopically expressed in HCT116 (**E**) and SW480 (**F**) cells. FXR1 ubiquitination was examined by IP with an anti-FXR1 antibody; **G**, **H** HA-UB and siOTUD6B were cotransfected into HCT116 (**G**) and SW480 (**H**) cells. FXR1 ubiquitination was analyzed by IP using an anti-FXR1 antibody. **I**, **J** HA-UB and Myc-FXR1 were ectopically coexpressed with either Flag-OTUD6B (**I**) or siOTUD6B (**J**) in HEK-293T cells. After MG132 treatment, FXR1 ubiquitination was assessed as described above; **K** Wild-type or lysine-specific ubiquitin mutants (K6, K11, K27, K29, K33, K48, and K63), Flag-OTUD6B, and Myc-FXR1 were cotransfected into HEK-293T cells. After MG132 treatment, FXR1 ubiquitination was analyzed by co-IP with anti-Myc antibody.

Based on the observation that OTUD6B enhances FXR1 stability, we investigated whether this effect is mediated through the blockade of ubiquitination and proteasomal degradation. Ectopic coexpression of Flag-OTUD6B, Myc-FXR1, and HA-ubiquitin (HA-UB) in HEK-293T cells, followed by coimmunoprecipitation analysis, demonstrated that compared with the control treatment, OTUD6B overexpression markedly decreased FXR1 ubiquitination (Fig. 5A). This effect was dose-dependent (Fig. 5B) and abolished by the catalytic mutant OTUD6B^C158S^ (Fig. 5C, Fig. S4A), indicating a reliance on the enzymatic activity of OTUD6B. Conversely, OTUD6B knockdown significantly increased FXR1 ubiquitination (Fig. 5D). Similar effects were observed in CRC cell lines, with OTUD6B overexpression reducing FXR1 ubiquitination (Fig. 5E, F), whereas OTUD6B knockdown led to increased FXR1 ubiquitination (Fig. 5G, H). These effects were consistent regardless of MG132 treatment: OTUD6B overexpression reduced FXR1 ubiquitination (Fig. 5I), whereas OTUD6B knockdown increased its ubiquitination (Fig. 5J). Notably, MG132 treatment further promoted ubiquitin accumulation, indicating that the degradation of FXR1 is proteasome-dependent. To examine linkage specificity, a panel of ubiquitin mutants (Ub-K6, K11, K27, K29, K33, K48, and K63) was employed. OTUD6B overexpression selectively removed K48-linked polyubiquitin from FXR1 (Fig. 5K). Collectively, these findings indicate that OTUD6B directly cleaves K48-linked polyubiquitin chains from FXR1 in a catalytic activity-dependent manner.

### The OTUD6B-FXR1 feedback loop sustains activation of the MEK2/ERK signaling pathway

**Fig. 6:**
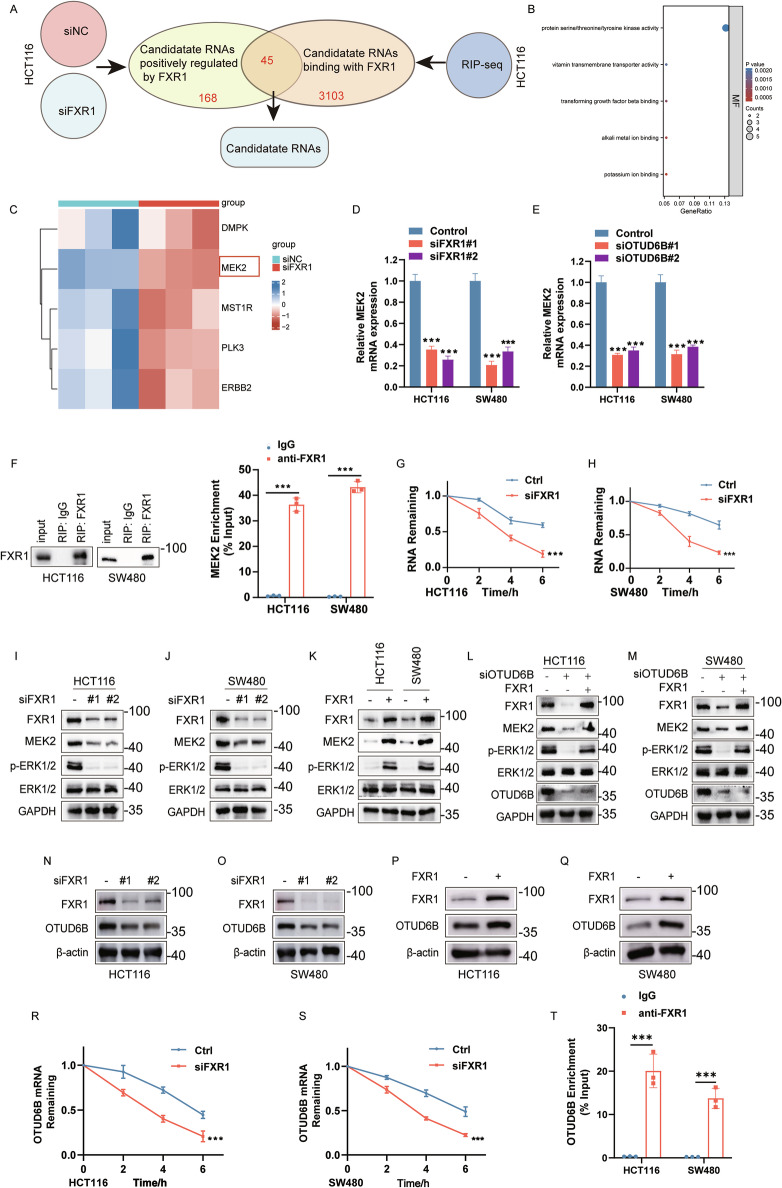
The OTUD6B–FXR1 feedback loop sustains activation of the MEK2/ERK signaling pathway. **A** Schematic diagram of the screening strategy combining RNA-seq of FXR1-silenced HCT116 cells and RIP-seq for FXR1-bound mRNAs. **B**, **C** KEGG enrichment and heatmap analysis identified enrichment of genes involved in kinase activity. **D**, **E** qRT–PCR analysis showed that MEK2 mRNA levels were significantly reduced upon knockdown of FXR1 (**D**) or OTUD6B (**E**) in CRC cells. **F** RIP-qPCR confirmed the direct binding between FXR1 and MEK2 mRNA. **G**, **H** Actinomycin D treatment (5 μg/mL) followed by qRT–PCR demonstrated accelerated MEK2 mRNA degradation upon FXR1 knockdown. **I–K** The protein levels of MEK2, pERK1/2 and total ERK1/2 were detected after FXR1 knockdown (**I**, **J**) and overexpression (**K**). **L**, **M** Detection of MEK2 and pERK1/2 protein levels after OTUD6B knockdown, with or without FXR1 overexpression; **N–Q** Detection of OTUD6B protein levels after FXR1 knockdown (**N**, **O**) or overexpression (**P**, **Q**). **R**, **S** Actinomycin D treatment (5 μg/mL) followed by qRT–PCR demonstrated accelerated OTUD6B mRNA degradation upon FXR1 knockdown. **T** RIP-qPCR confirmed the direct binding between FXR1 and OTUD6B mRNA; Statistical significance was determined using two-tailed Student’s *t* test and ANOVA. ***p < 0.001.

To identify downstream targets of FXR1, an RNA-binding protein implicated in mRNA stability [24–26], RIP-seq and transcriptome analysis were integrated to profile candidate mRNAs bound by FXR1 (Fig. 6A). KEGG pathway enrichment analysis of mRNAs that were both significantly enriched by RIP-seq and positively regulated by FXR1 revealed a predominance of genes involved in protein kinase activity, particularly serine/threonine/tyrosine kinases (Fig. 6B, C). Among these, MEK2, a core kinase in the MAPK pathway, emerged as a potential key FXR1 target. Further qRT–PCR experiments confirmed that MEK2 mRNA expression is positively regulated by FXR1 (Fig. 6D). Notably, MEK2 levels were also reduced upon OTUD6B silencing (Fig. 6E), indicating that MEK2 functions as a downstream target of the OTUD6B/FXR1 axis. RIP assays demonstrated a direct interaction between FXR1 and MEK2 mRNA (Fig. 6F), and RNA stability assays following actinomycin D treatment revealed accelerated MEK2 mRNA decay upon FXR1 depletion (Fig. 6G, H). Western blot analysis revealed that FXR1 knockdown suppressed MEK2 and phosphorylated ERK1/2 (pERK1/2) levels without affecting total ERK1/2 protein levels (Fig. 6I, J), whereas FXR1 overexpression increased MEK2 and pERK1/2 expression (Fig. 6K). FXR1 overexpression reversed the downregulation of MEK2/pERK1/2 induced by OTUD6B knockdown (Fig. 6L, M).

Interestingly, transcriptomic analysis revealed a significant reduction in OTUD6B mRNA expression upon FXR1 silencing (Fig. S4B), suggesting potential feedback regulation. This finding was further confirmed by qRT–PCR and Western blot analyses, which demonstrated that FXR1 positively regulates OTUD6B expression at both the mRNA and protein levels (Fig. S4C, Fig. 6N–Q). To investigate the potential regulatory mechanism, we conducted exploratory actinomycin D assays and found that silencing FXR1 expression significantly accelerated the degradation of OTUD6B mRNA, suggesting that FXR1 likely regulates OTUD6B at the posttranscriptional level (Fig. 6R, S). Consistent with these findings, RIP assays confirmed the direct interaction between FXR1 and OTUD6B mRNA (Fig. 6T). Collectively, OTUD6B and FXR1 establish a positive feedback loop that sustains the activation of the MEK2/ERK signaling pathway.

### MEK2 inhibition cooperates with OTUD6B depletion to suppress CRC growth and liver metastasis

**Fig. 7:**
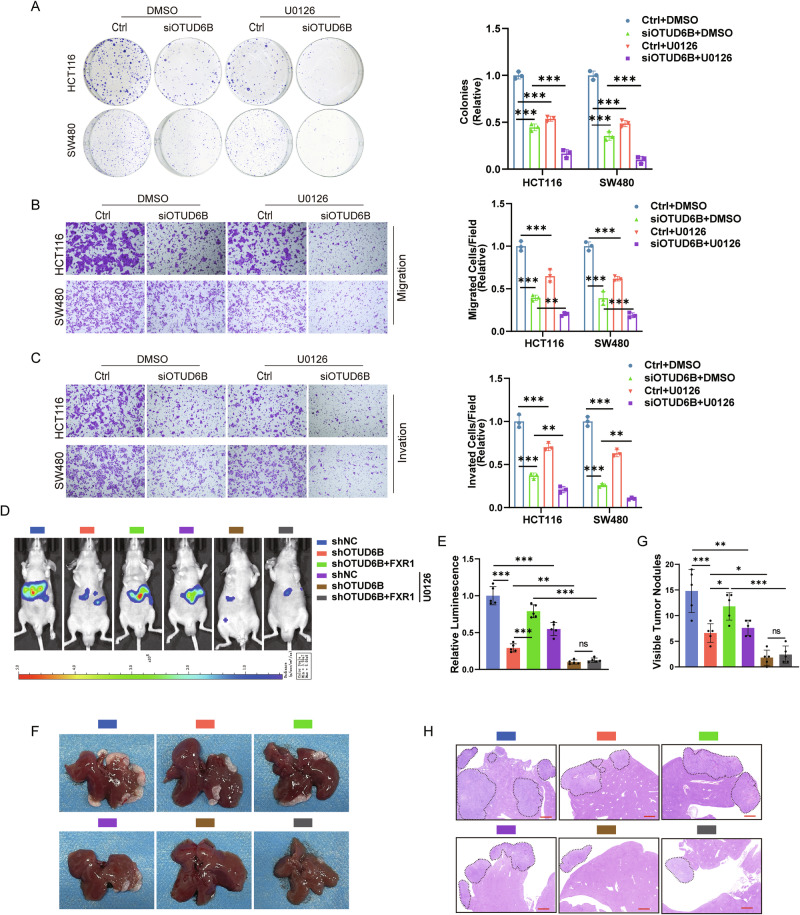
MEK2 inhibition with U0126 enhances the suppressive effect of OTUD6B depletion on CRC growth and dissemination. **A** Colony formation assays were performed in HCT116 and SW480 cells treated with control or OTUD6B-targeting siRNA and exposed to DMSO or U0126 (10 μM). Colony numbers per well were quantified. **B**, **C** Transwell migration (**B**) and Matrigel invasion (**C**) assays were performed in cells treated as described in (**A**). **D–H** Liver metastases were analyzed in splenic injection models. Experimental liver metastasis models were generated from HCT116 cells with stable OTUD6B knockdown, with or without FXR1 overexpression; U0126 (15 mg/kg) or PBS was administered intraperitoneally every 3 days. Representative in vivo fluorescence images (**D**), quantification of hepatic metastases (**E**), macroscopic liver surface nodules (**F**, **G**), and histological evaluation of metastatic lesions (**H**) are shown. Scale bar: 1 mm. Statistical significance was assessed using one-way ANOVA for comparisons among more than two groups. ns = no significant difference; *p < 0.05; **p < 0.01; ***p < 0.001.

To elucidate the role of MEK2/ERK signaling in the OTUD6B-mediated malignant behavior of CRC, we treated cells with the MEK2 inhibitor U0126 and evaluated their proliferative and metastatic capacities. U0126 synergistically enhanced the inhibitory effect of OTUD6B silencing on CRC cell proliferation, migration and invasion (Fig. 7A–C). Furthermore, U0126 combined with OTUD6B silencing significantly reduced CRC liver metastasis in nude mice, and overexpression of FXR1 reversed the inhibitory effect of OTUD6B silencing; however, this reversal was completely abolished by U0126 treatment (Fig. 7D–H). Collectively, these findings indicate that OTUD6B promotes CRC progression through the FXR1–MEK2–ERK axis and that combining OTUD6B silencing with U0126 may represent a promising therapeutic strategy to suppress CRC liver metastasis.

### OTUD6B, FXR1, and MEK2 expression are positively correlated in primary and metastatic CRC tissues

**Fig. 8:**
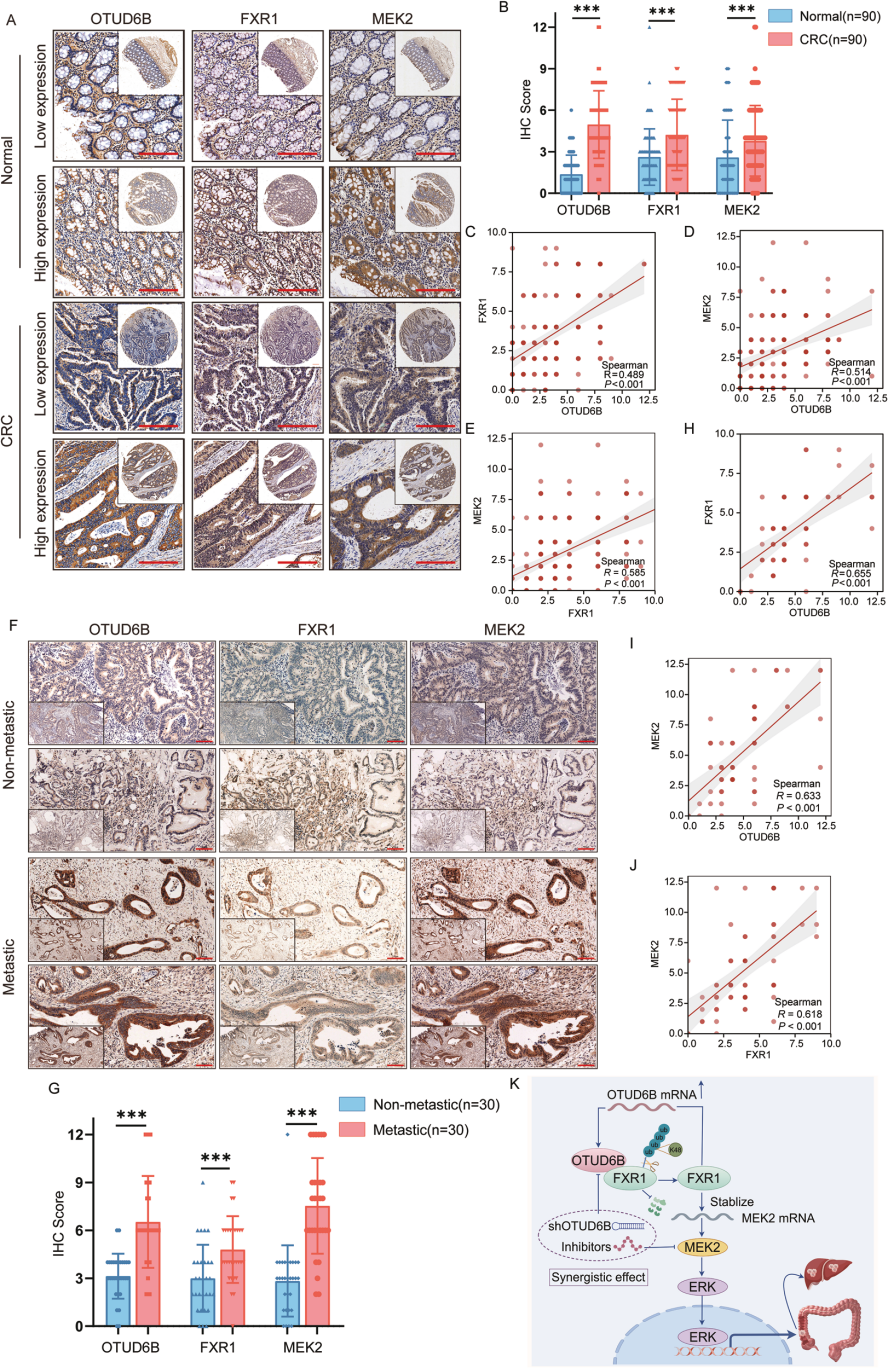
OTUD6B expression is positively correlated with the expression levels of FXR1 and MEK2 in primary and metastatic CRC tissues. **A**, **B** Representative IHC images and quantification of OTUD6B, FXR1, and MEK2 expression in 90 paired CRC and adjacent normal tissues; scale bar: 200 μm. **C–E** Spearman correlation analysis of OTUD6B, FXR1, and MEK2 expression in CRC tissues. **F**, **G** Representative IHC images and quantification of OTUD6B, FXR1, and MEK2 expression in 30 liver-metastatic and 30 nonmetastatic CRC samples; scale bar: 100 μm. **H–J** Spearman correlation confirmed robust coexpression of OTUD6B, FXR1, and MEK2 in liver-metastatic CRC. **K** Schematic diagram of the OTUD6B/FXR1/MEK2/ERK axis in the regulation of CRC liver metastasis; statistical significance was determined using two-tailed Student’s *t* test and Spearman correlation analysis. ***p < 0.001.

To investigate the clinical relevance of OTUD6B in relation to that of FXR1 and MEK2 in CRC, we evaluated their expression in a tissue microarray containing 90 paired CRC and adjacent normal samples. IHC analysis demonstrated markedly elevated expression of OTUD6B, FXR1, and MEK2 in CRC tissues compared with matched adjacent mucosa. Notably, compared with patients with low OTUD6B expression, patients with high OTUD6B expression presented significantly increased levels of FXR1 and MEK2 (Fig. 8A, B). Spearman correlation analysis further revealed strong positive correlations among OTUD6B, FXR1, and MEK2 expression levels (Fig. 8C–E).

To further assess their relevance to metastasis, 30 liver-metastatic and 30 nonmetastatic CRC samples were subjected to IHC staining. The expression levels of OTUD6B, FXR1, and MEK2 were significantly higher in liver-metastatic CRC tissue samples than in nonmetastatic samples (Fig. 8F–G). Notably, Spearman correlation analysis revealed robust positive correlations among OTUD6B, FXR1, and MEK2 in metastatic CRC (Fig. 8H–J). Taken together, these results indicate that OTUD6B is upregulated in liver metastatic CRC and strongly correlates with FXR1 and MEK2, suggesting that OTUD6B may promote CRC progression and metastasis through the FXR–MEK2 signaling axis.

## Discussion

Recent advances have highlighted the pivotal role of the OTU family of DUBs in tumor initiation and progression [7, 8, 27, 28]. As modulators of protein stability and signaling cascades, OTU DUBs represent promising candidates for targeted cancer therapy. In this study, we identified OTUD6B as a critical OTU family member whose expression is progressively upregulated from normal colonic mucosa to primary tumors and is further elevated in metastatic CRC tissues. Functional analyses revealed that OTUD6B promotes CRC cell proliferation, migration, invasion, and liver metastasis both in vitro and in vivo. Mechanistically, OTUD6B stabilizes the RNA-binding protein FXR1 through removing its ubiquitination, which in turn enhances MEK2 mRNA stability, leading to sustained activation of the ERK signaling pathway. Intriguingly, FXR1 also reinforces OTUD6B expression, resulting in the formation of a positive feedback loop that amplifies the oncogenic OTUD6B–FXR1–MEK2–ERK axis. Moreover, a MEK2 inhibitor combined with OTUD6B silencing significantly suppressed CRC liver metastasis (Fig. 8K).

Previous studies have demonstrated that OTUD6B plays a critical role in regulating tumor growth and metastasis [9, 13, 29]. The oncogenic potential of OTUD6B is further supported by our studies, in which silencing OTUD6B significantly impaired tumor growth and reduced liver metastatic nodules. In recent years, the identification of multiple OTUD6B substrates has provided important insights into its enzymatic activity and chain specificity. Although some reports suggest that OTUD6B can regulate pVHL protein independently of its DUB activity [9], the catalytic site of OTUD6B is indispensable for regulating the ubiquitination of other substrates such as β-TrCP [10], LIN28B [12], PTK2 [11], and KIFC1 [13]. In line with these findings, our data demonstrate that OTUD6B regulates FXR1 protein stability in a DUB activity-dependent manner. Although the catalytic domain of OTUD6B resides at the C-terminus, we found that its N-terminal domain is essential for FXR1 binding, a pattern consistent with previous studies showing OTUD6B’s N-terminal interactions with KIFC1 and IRF3 [13, 30]. FXR1 contains three principal domains—Agenet, KH, and RGG—and the KH and RGG domains are generally considered central mediators of protein–protein interactions [31, 32]. In support of this, Wang et al. reported that the KH domain of FXR1 is critical for its interaction with CEP63 [33]. In line with these findings, our study demonstrated that the KH domain likewise plays a central role in mediating the direct binding of FXR1 to OTUD6B.

FXR1 has attracted considerable attention owing to its prominent oncogenic effects across multiple tissues [34, 35]. In this study, we identified FXR1 as a critical downstream effector of the oncogenic activity of OTUD6B. Ectopic expression of FXR1 effectively rescued the impaired proliferative, migratory, and invasive capacities induced by OTUD6B silencing. Notably, despite the significance of FXR1 in cancer, few studies have investigated its ubiquitination regulation. Prior research revealed that the E3 ligase Fbxo4 ubiquitinates FXR1 at an undefined lysine in head and neck squamous cell carcinoma, leading to its proteasomal degradation [36]. CEP63 has also been shown to remove K63-linked ubiquitin chains from FXR1 [33]. Our findings provide new mechanistic insight, demonstrating that OTUD6B specifically removes K48-linked ubiquitin chains from FXR1, thus increasing the current understanding of the posttranslational regulation of FXR1. As a DUB, the primary function of OTUD6B is to selectively remove ubiquitin modifications from its substrates. Previous studies have reported that OTUD6B cleaves K11- and K33-linked ubiquitin chains from IRF3 [30], removes K11-linked ubiquitination from KIFC1 [13], and eliminates K48-linked ubiquitination of LIN28B [12]. Consistent with these findings, our study demonstrated that OTUD6B specifically removes K48-linked ubiquitin chains from FXR1, thereby increasing the stability of the FXR1 protein.

FXR1 contributes to cellular transformation and promotes tumor cell invasion and migration by regulating mRNA translation, alternative splicing, polyadenylation, and stability [37, 38]. To identify FXR1-bound transcripts, we performed a RIP assay combined with RAN-seq, as well as transcriptomic profiling following FXR1 knockdown. MEK2 mRNA emerged as a top candidate. This interaction was confirmed by RIP–qPCR, and assays revealed that silencing FXR1 accelerated MEK2 mRNA decay, resulting in decreased MEK2 protein levels and attenuated ERK1/2 phosphorylation. In contrast, FXR1 overexpression increased MEK2 expression and downstream ERK activation. These data support a model in which FXR1 regulates MEK2 posttranscriptionally to sustain oncogenic ERK signaling. Importantly, protein expression analyses from two independent clinical CRC cohorts confirmed a positive correlation between OTUD6B, FXR1, and MEK2, particularly in metastatic lesions, further supporting the relevance of this signaling axis in CRC.

Currently, no specific inhibitors of OTUD6B have been reported, leaving a substantial gap in the development of OTUD6B-targeted therapies. U0126, a well-characterized MEK inhibitor, has been shown to block MEK/ERK signaling and suppress tumor progression across multiple models, including oral squamous cell carcinoma [39], esophageal squamous cell carcinoma [40], and CRC [41]. In our study, the combination of U0126 and OTUD6B knockdown synergistically suppressed CRC metastasis both in vitro and in vivo, indicating the translational significance of targeting this axis.

In addition to the mechanistic insights provided by our study, several limitations and translational considerations should be acknowledged. First, while we observed that OTUD6B expression was markedly lower in normal colonic epithelial NCM460 cells than in CRC cell lines, a limitation of the present work is that we did not systematically assess the functional impact of OTUD6B inhibition on normal epithelial cells. Nonetheless, the tumor-enriched expression pattern suggests that therapeutic targeting of OTUD6B may have minimal impact on normal tissues. Previous reports have shown that OTUD6B can interact with multiple substrates—such as LIN28B [12], PTK2 [11], and KIFC1 [13]—in diverse biological contexts, implying that its substrate spectrum and functional roles are likely context-dependent. Although the relevance of these interactions in CRC remains to be determined, future studies should systematically investigate the substrate network of OTUD6B and employ structure-guided inhibitor design, multitarget screening, and multiple independent RNAi/CRISPR reagents with rescue experiments to ensure target specificity and minimize off-target effects. Second, all in vivo experiments in this study were conducted in immunocompromised nude mice, which lack a fully functional adaptive immune system. This model allowed us to focus on the tumor cell–intrinsic effects of the OTUD6B/FXR1 axis on CRC growth and liver metastasis; however, it does not fully recapitulate tumor–immune interactions within the metastatic microenvironment. Future research using immunocompetent or humanized CRC models will be important to further evaluate how OTUD6B inhibition influences immune regulation and to enhance translational relevance. Finally, although correlative analyses using clinical CRC samples support the coexpression of OTUD6B, FXR1, and MEK2, larger multicenter cohorts with detailed clinicopathological annotations and long-term follow-up data are needed to confirm their prognostic value and ability to predict therapeutic response.

## Conclusion

This study reveals a novel oncogenic cascade in which OTUD6B stabilizes FXR1 through regulating K48-linked ubiquitination, leading to MEK2 mRNA stabilization and ERK pathway activation. This OTUD6B–FXR1–MEK2/ERK axis promotes CRC progression and liver metastasis. Moreover, the synergistic effects observed upon combining OTUD6B depletion with MEK inhibition underscore the therapeutic potential of dual-targeting strategies. These findings offer a compelling rationale for further preclinical and clinical development of interventions that disrupt this oncogenic circuit in metastatic CRC.

## Materials and Methods

### Cell culture

For all lines, complete medium consisted of the indicated basal medium supplemented with 10% fetal bovine serum and 1% penicillin-streptomycin. NCM-460, SW620, DLD-1, and HT29 were cultured in high-glucose Dulbecco’s modified Eagle’s medium (DMEM). HCT116 was maintained in McCoy’s 5 A, and SW480 was maintained in Leibovitz’s L-15. Cultures were kept at 37 °C in a humidified incubator; the atmospheric conditions were set according to the recommendations for each medium.

### Tissue samples

Two types of clinical CRC samples were analyzed. The first cohort comprised treatment-naïve patients with CRC, comprising patients both with and without liver metastases, who underwent radical resection at the Second Affiliated Hospital of Nanchang University; all the patients were confirmed to have adenocarcinoma by preoperative imaging and postoperative pathology, and none had received neoadjuvant radiotherapy or chemotherapy. The study was conducted in accordance with the Declaration of Helsinki and was approved by the Institutional Ethics Committee of the Second Affiliated Hospital of Nanchang University (Approval NO. 2025-97). The second cohort consisted of commercial CRC tissue microarrays—Ade180CS-01 (Shanghai Outdo Biotech, Shanghai, China) and ZL-Cocsur1801 (Zhuoli Biotechnology, Shanghai, China)—containing 90 paired formalin-fixed, paraffin-embedded CRC tissues and matched adjacent noncancerous tissues, each reviewed by pathologists. Complete clinicopathological data (demographic features, tumor location, histological grade, and TNM stage) were provided by the suppliers. Hematoxylin and eosin (H&E) staining was used to confirm tissue integrity prior to analysis.

### Subcutaneous xenograft and liver metastasis models

Four-week-old male BALB/c nude mice (5–6 per group; GemPharmatech Co., Ltd., Nanjing, China) were acclimated for 5–7 days before experimentation. For the subcutaneous xenograft model, 1 × 10^7^ HCT116 cells in a 1:1 mixture with Matrigel were injected into the axillary subcutaneous region. Tumor growth was measured weekly by length (L) and width (W), and tumor volume was calculated as V = 0.5 × L × W². Mice were euthanized when the tumors reached the predetermined size. For the liver metastasis model, 2 × 10^6^ HCT116 cells in 100 μL of PBS were injected into the splenic parenchyma of anesthetized nude mice. U0126 was dissolved in DMSO, diluted in PBS (final DMSO ≤ 10%), and administered intraperitoneally at 15 mg/kg every 3 days; control mice received equal volumes of PBS. Hepatic metastases were assessed weekly by in vivo bioluminescence imaging (IVIS system) following intraperitoneal injection of D-luciferin. At the endpoint, the mice were euthanized, and liver tissues were harvested, fixed in 4% paraformaldehyde, and subjected to histological analysis. All animal experiments were approved by the Nanchang University Animal Care Committee (Approval NO. NCULAE-20250509002).

### Antibodies and purified proteins

Antibodies used in this study: anti-OTUD6B (Proteintech, 25430-1-AP), anti-β-actin (Proteintech, HRP-60008), anti-Ki-67 (Proteintech, 27309-1-AP), anti-FXR1 (Santa Cruz, sc-374148), anti-Myc (Cell Signaling Technology, #2276; Proteintech, 10828-1-AP), anti-HA (Cell Signaling Technology, #3724), anti-Flag (Proteintech, 66008-4-Ig, 20543-1-AP), anti-GAPDH (Proteintech, HRP-60004), anti-MEK2 (Proteintech, 67410-1-Ig), anti-ERK1/2 (Proteintech, 11257-1-AP), anti-p-ERK1/2 (Cell Signaling Technology, #4370), anti-HNRNPU (Proteintech, 14599-1-AP), anti-TIA1 (Proteintech, 12133-2-AP), anti-RBM4B (Proteintech, 15412-1-AP), anti-RBM14 (Proteintech, 10196-1-AP), anti-SRSF3 (HUABIO, ET7109-47), anti-E-cadherin (Proteintech, 20874-1-AP), anti-N-cadherin (Proteintech, 22018-1-AP), anti-MMP9 (Proteintech, 10375-2-AP), anti-TIMP1 (Proteintech, 16644-1-AP). Purified GST-OTUD6B protein was produced by Novoprotein (Suzhou, China). Purified GST protein was obtained from Proteintech (Ag0040, Wuhan, China).

### Statistical analysis

Quantitative data are expressed as the mean ± standard deviation (SD). Differences between two groups were analyzed using two-tailed, independent-samples t tests, and comparisons among three or more groups were evaluated by one-way ANOVA with appropriate post hoc multiple-comparison tests. Statistical analyses were conducted using SPSS v26.0 (IBM, Armonk, NY, USA) or GraphPad Prism v9.0 (GraphPad Software, San Diego, CA, USA). A *p*-value < 0.05 was considered to indicate statistical significance. Data visualization and figure assembly were performed with GraphPad Prism v9.0 and Adobe Illustrator CC 2021.

## Supplementary information

Supplementary materials

Original Western Blot

aj-checklist

## Data Availability

All data generated or analyzed during this study are included in this published article and its supplementary information files.
