# Supplementary material for: OTUD6B-mediated K48 deubiquitination of FXR1 forms a positive feedback loop activating MEK2/ERK signaling in colorectal cancer liver metastasis
Source: Cell Death Dis. 2026 Apr 29;17(1):572. doi: 10.1038/s41419-026-08812-z (PMC13269552; doi:10.1038/s41419-026-08812-z)
Supplement: Supplementary file 1 — Supplementary materials [file 41419_2026_8812_MOESM1_ESM.docx]

## Supplementary materials

**OTUD6B-mediated K48 deubiquitination of FXR1 forms a positive feedback loop activating MEK2/ERK signaling in colorectal cancer liver metastasis**

Ying Lu^1, *^, Ji Liu^2, 3, *^, Ya-Nan Li^1^, Lin xiang^1^, Guo-Bin Song^1^, Tian Peng^1^, Zhen Wang^1^, Xue Yang^1^, Hou-Qun Ying^1, #^, Xue-Xin Cheng^1, #^

*^1^Jiangxi Province Key Laboratory of Immunology and Inflammation, Jiangxi Provincial Clinical Research Center for Laboratory Medicine, Department of Clinical Laboratory, The Second Affiliated Hospital, Jiangxi Medical College, Nanchang University, Nanchang, Jiangxi, China.*

*^2^Department of Transplantation, Jiangxi Provincial People’s Hospital, The First Affiliated Hospital of Nanchang Medical College, Nanchang, Jiangxi, China.*

*^3^Jiangxi Provincial Key Laboratory of Urinary System Diseases, Department of Urology, The First Affiliated Hospital, Jiangxi Medical College, Nanchang University, Nanchang, Jiangxi, China.*

*^*^These authors contributed equally to this work：Ying Lu, Ji Liu.*

*^#^Correspondence:* *Xue-Xin Cheng, Email: [cxxncu@163.com](mailto:cxxncu@163.com); Hou-Qun Ying, Email: [yinghouqun2013@163.com](mailto:yinghouqun2013@163.com).*

**Supplementary Figures, Supplementary Tables, Supplementary Methods**

## Supplementary Figures


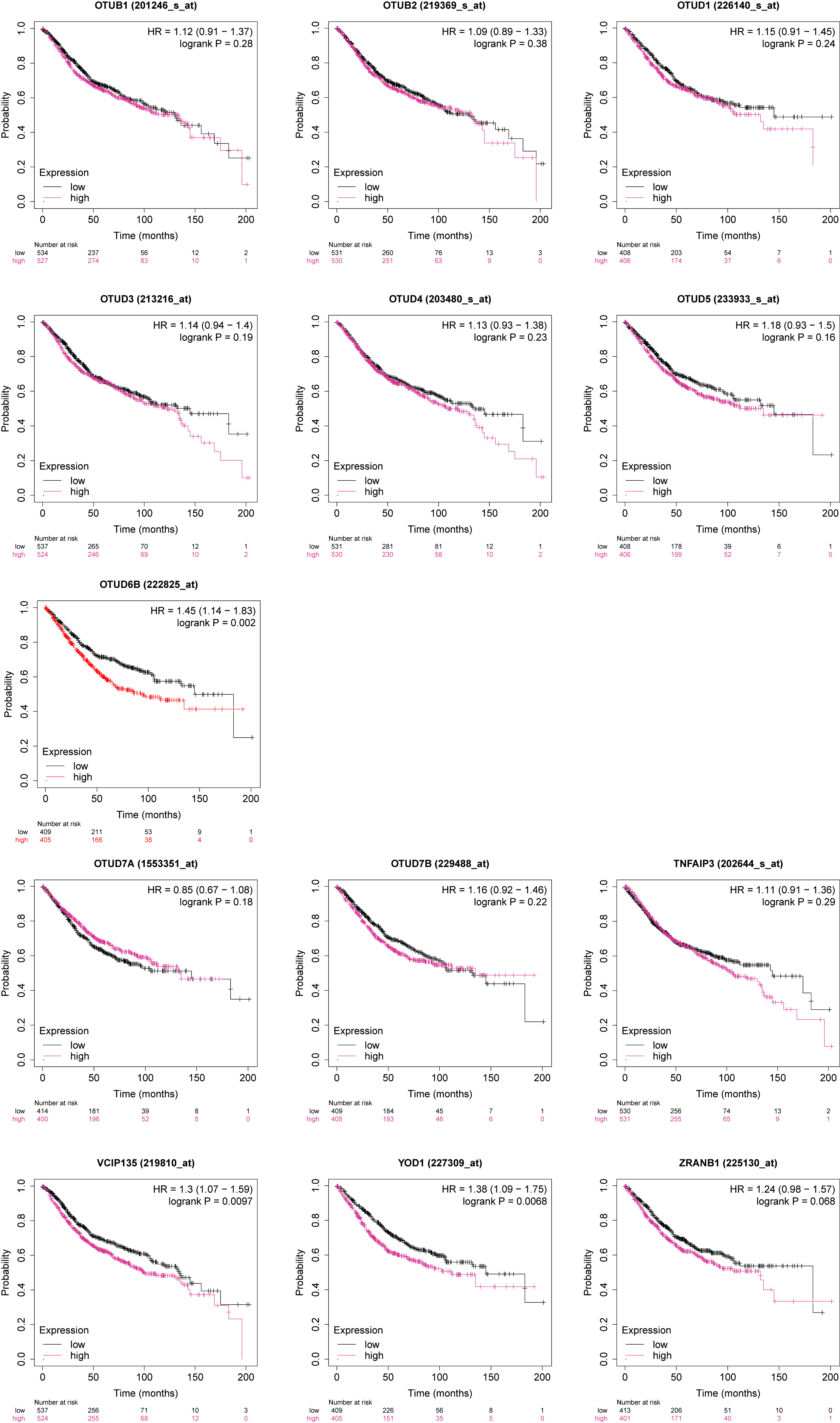


**Fig. S1 The prognostic roles of OTU family members in CRC are analyzed using the Kaplan–Meier Plotter database.**


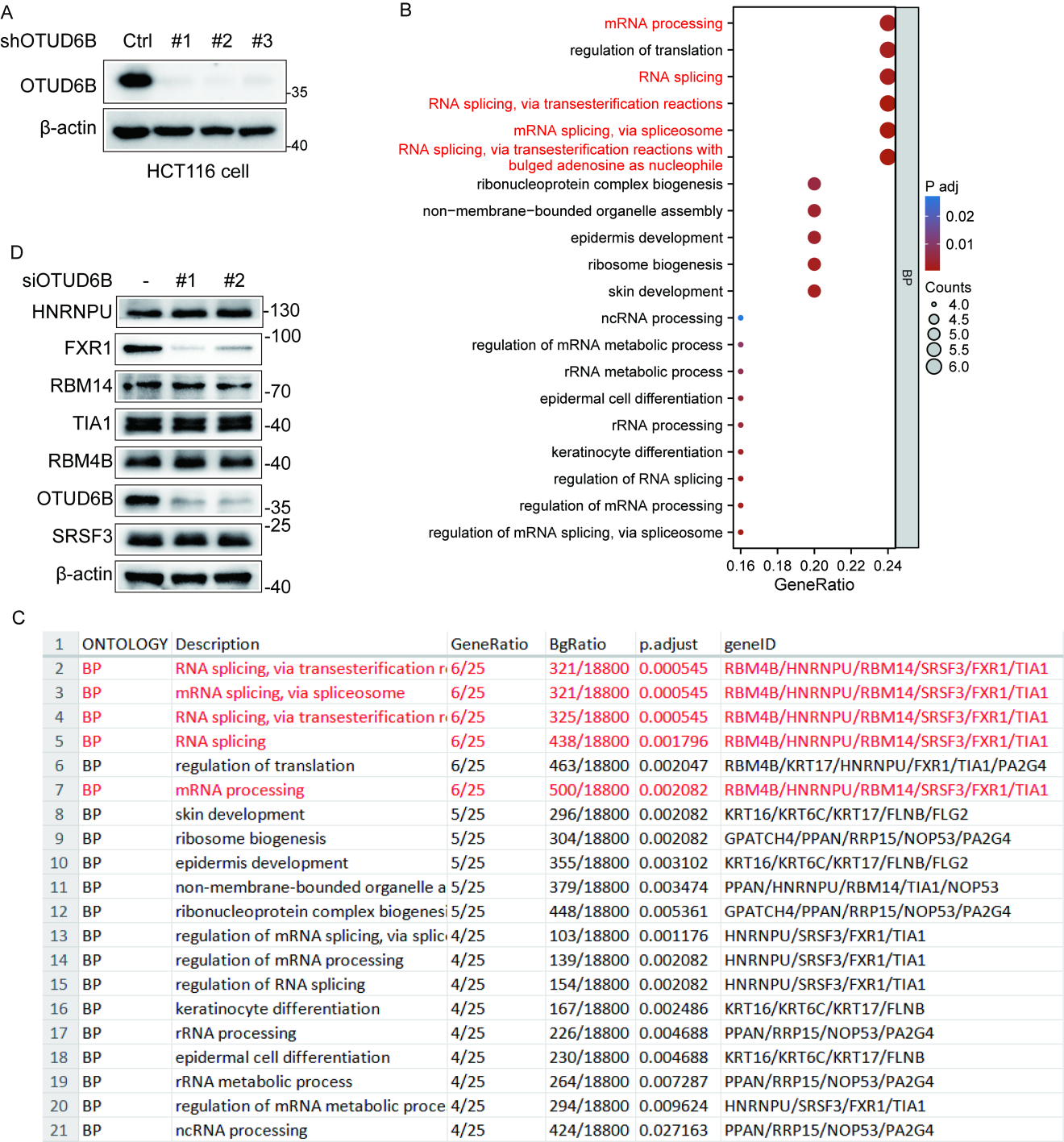


**Fig. S2 A.** Establishment and validation of OTUD6B stably knockdown cell line; **B, C** Gene Ontology (GO) enrichment analysis of OTUD6B-interacting proteins (B); The top 20 enriched GO terms are shown (C); **D** Protein levels of HNRNPU, FXR1, RBM14, TIA1, RBM4B, and SRSF3 were measured in HCT116 cells following OTUD6B knockdown.

**
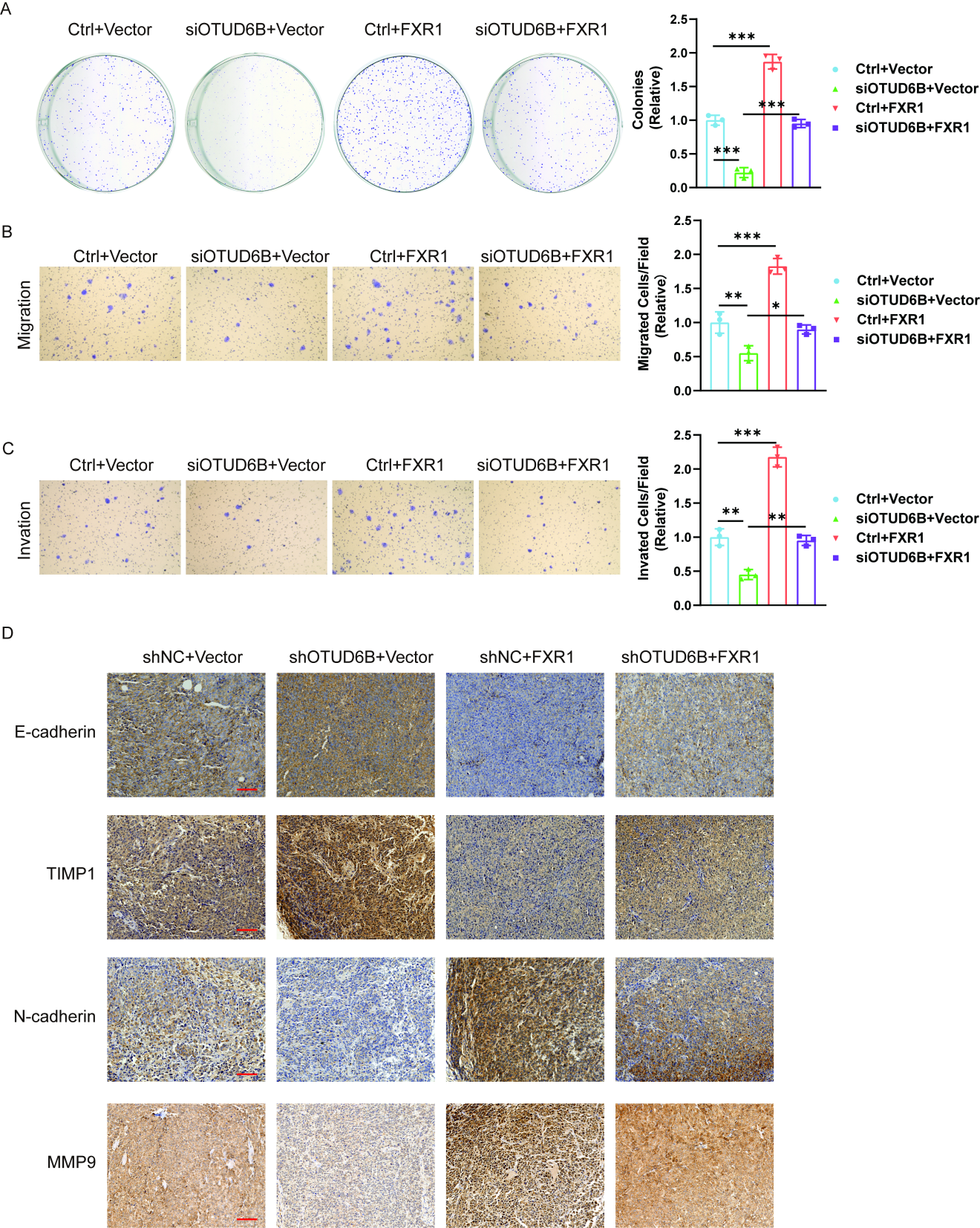
Fig. S3 A–C** HT29 cells were transfected with OTUD6B siRNAs or FXR1 plasmids (with or without cotransfection of OTUD6B siRNAs), followed by colony formation assays (A) and Transwell migration (B) and invasion assays (C); **D** Detection of EMT markers (E-cadherin and N-cadherin) and metastasis-associated markers (MMP9 and TIMP1) in tumor tissues from subcutaneous xenograft models.

**
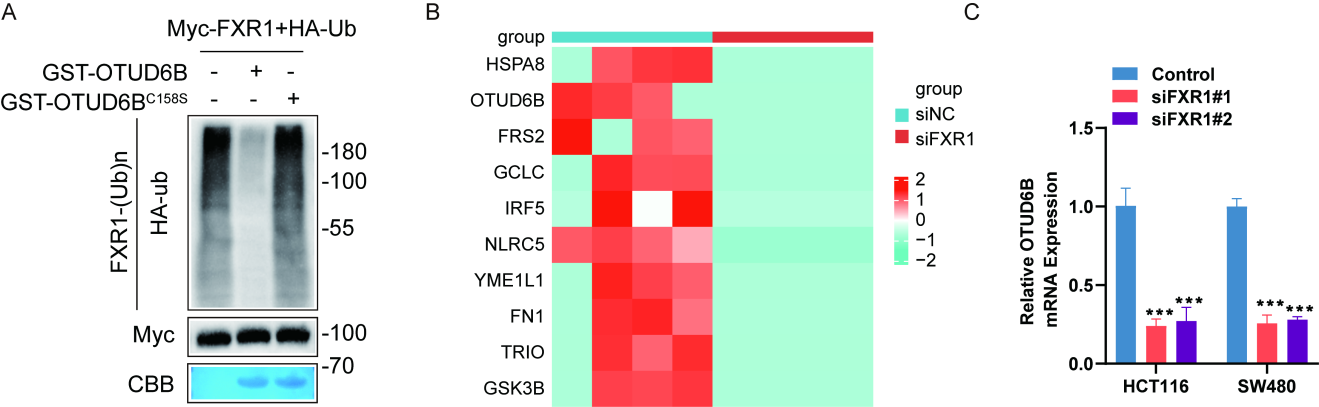
Fig. S4 A** Ubiquitinated Myc-FXR1 was immunoprecipitated from MG132-treated HEK-293T cells co-transfected with Myc-FXR1 and HA-Ub. The immunoprecipitates were then incubated with purified GST-OTUD6B or the catalytically inactive mutant GST-OTUD6B^C158S^ in deubiquitination buffer, and FXR1 ubiquitination was analyzed by Western blotting using an anti-HA antibody; **B** Top10 downregulated genes following FXR1 knockdown in HCT116 cells; **C** Detection of relative OTUD6B mRNA levels in CRC cells after FXR1 knockdown by qRT–PCR. ****p* < 0.001.

## Supplementary Tables

**Table S1.** **Transcriptomic profiling of OTU family genes in CRC as determined by whole**–**transcriptome sequencing**

| **Gene** | **Log_2_Fold Change** | **adj.P.Val** |
| --- | --- | --- |
| OTUB2 | 1.490977179 | 0.025426551 |
| OTUD6B | 1.431288333 | 0.001005146 |
| OTUD7A | -1.379329375 | 0.028628494 |
| TNFAIP3 | -0.766391173 | 0.105570241 |
| ZRANB1 | -0.693390253 | 0.111878523 |
| OTUD7B | -0.472466579 | 0.272518274 |
| OTUB1 | 0.412049041 | 0.236084278 |
| OTUD1 | -0.364043134 | 0.272518274 |
| YOD1 | 0.344108271 | 0.272518274 |
| VCPIP1 | 0.329932325 | 0.272940093 |
| OTUD5 | -0.227276322 | 0.413486473 |
| OTUD3 | -0.13958553 | 0.729982991 |
| OTUD4 | -0.008310437 | 0.976580604 |

**Table S2. Primers used in this study**

| **Gene name** | **Forward Primer** | **Reverse Primer** |
| --- | --- | --- |
| GAPDH | CAAGGCTGTGGGCAAGGTCATC | GTGTCGCTGTTGAAGTCAGAGGAG |
| FXR1 | CTGCGACAGATTGGTTCTAGG | TGTACCATAACCGGAGGTGTAA |
| OTUD6B | CTGCTGAGAAGGCATCGCAAAG | GCCACATCTTCGGTGAGTTGCT |
| MEK2 | ACATCGTGGGCTTCTACGG | TTGGCCTCTTTCAGCACCT |

**Table S3. Sequences of siRNAs and shRNAs used in this study**

| **siRNA** | | **sense（5'-3'）** | **antisense（5'-3'）** |
| --- | --- | --- | --- |
| siOTUD6B#1 | GAGACAUGCAUAUGGCUUATT | | UAAGCCAUAUGCAUGUCUCTT |
| siOTUD6B#2 | CACGGUUGGUAAACAUAGUTT | | ACUAUGUUUACCAACCGUGTT |
| siFXR1#1 | GAGGUUUCUUGGAAUUUGUTT | | ACAAAUUCCAAGAAACCUCTT |
| siFXR1#2 | CCUCCGGUUAUGGUACAAATT | | UUUGUACCAUAACCGGAGGTT |

**Table S4. Sequences of shRNAs used in this study**

| **Gene** | **sequences** |
| --- | --- |
| shOTUD6B#1 | CGAGAAGAACGGATAGCTGAA |
| shOTUD6B #2 | GCTGACTACTAAGGAGAATAA |
| shOTUD6B #3 | CGATGAGACTAATGCAGTGAA |

## Supplementary methods

### Western blotting (WB)

Proteins were extracted using RIPA buffer (WB3100; NCM Biotech, Suzhou, China) containing PMSF and additional protease/phosphatase inhibitors. Concentrations were quantified by a BCA protein assay kit (PA115; TransGen Biotech, Beijing, China). Equal protein samples underwent electrophoresis using 10% SDS–PAGE gels and were subsequently transferred onto PVDF membranes. The membranes were blocked in 5% skim milk at room temperature for 1 h, then incubated overnight at 4 °C with specific primary antibodies. After washing three times (5 min each) with TBST (Tris-buffered saline, 0.1% Tween-20), membranes were incubated with HRP-conjugated secondary antibodies (1:5000 dilution; Proteintech, Wuhan, China) for 2 h at room temperature. Following another set of TBST washes, protein bands were detected using enhanced chemiluminescence (ECL) reagent (P10060; NCM Biotech, Suzhou, China) and visualized through a chemiluminescent imaging system.

### Immunohistochemistry (IHC)

Paraffin-embedded tissue sections were dewaxed, and endogenous peroxidase activity was blocked with 3% hydrogen peroxide for 10 min. Antigen retrieval was performed in 10 mM sodium citrate buffer (pH 6.0), followed by overnight incubation at 4 °C with primary antibodies. Sections were then incubated with biotinylated secondary antibodies at 37 °C for 30 min, and signals were visualized by chromogenic detection. Immunostaining was independently evaluated by two pathologists. Staining intensity was graded as 0 (negative), 1 (weak), 2 (moderate), or 3 (strong), and the percentage of positive tumor cells was scored as 1 (≤25%), 2 (26–50%), 3 (51–75%), or 4 (>75%). The final IHC score was obtained by multiplying these two parameters, yielding a range from 0 to 12.

### Quantitative real–time PCR

Total RNA was extracted with a commercial kit (19231ES50; Yeasen, Shanghai, China) according to the manufacturer’s protocol (chloroform phase separation, isopropanol precipitation, 75% ethanol wash, air-drying, and resuspension in RNase-free water). RNA yield and purity were assessed using a NanoDrop 2000 spectrophotometer (A260/A280). First-strand cDNA was synthesized from 1 μg of total RNA using a reverse transcription kit (KR106; TIANGEN, Beijing, China). Quantitative PCR was performed with a SYBR Green qPCR kit (11184ES08; Yeasen) and gene-specific primers (Sangon Biotech, Shanghai, China) according to the manufacturers’ instructions. Transcript levels were normalized to GAPDH. Primer sequences are provided in **Table S2**.

### Plasmid and small–interfering RNA transfection

Cells were seeded into six-well plates and cultured overnight to reach 80–90% confluence. For plasmid transfection, 2 µg plasmid DNA and 4–6 µL PEI reagent were separately diluted in Opti-MEM, incubated for 5 min, combined, and incubated for an additional 20 min at room temperature. For siRNA transfection, complexes were prepared by diluting siRNA in 200 µL Opti-MEM and Lipofectamine 2000 (5–10 µL) in 200 µL Opti-MEM, incubating each for 5 min, then combining and incubating for 20 min to allow complex formation. Transfection mixtures were added dropwise and incubated with cells for 4–6 h, after which the medium was replaced with complete medium. Cells were harvested 24–48 h post-transfection for RNA extraction or 48–72 h post-transfection for protein extraction, depending on the experimental design. The corresponding plasmids were designed and synthesized by Hanbio Biotechnology (Shanghai, China). The detailed siRNAs sequences are listed in **Table S3.**

### Lentivirus–mediated RNA interference

Cells were seeded into six-well plates and transduced with a viral mixture consisting of serum-free medium, HitransG P transduction reagent, and virus volume calculated according to titer. After 16 h of incubation, the medium was replaced with fresh complete medium. Stable knockdown cells were generated by puromycin selection, and OTUD6B silencing efficiency was confirmed by Western blotting. Transduced cells were maintained under low-dose puromycin pressure to suppress non-infected cells and expanded for subsequent assays, including in vivo experiments. The detailed shRNA sequences are listed in **Table S4.**

### Co-immunoprecipitation (Co-IP) analysis mass spectrometric analyses

Cells were lysed in ice-cold IP lysis buffer and cellular debris was removed by centrifugation. Anti-Flag magnetic beads (25–50 μL) were pre-equilibrated by three washes with 500 μL lysis buffer. Clarified lysates were combined with the beads and incubated overnight at 4 °C on an end-over-end rotator to promote binding. Beads were collected on a magnetic rack and washed three times with lysis buffer to minimize nonspecific interactions. Bound proteins were eluted by adding 50 μL 1× SDS sample buffer, vortexing, and heating at 95–100 °C for 5–10 min in a metal heating block. The eluates were subsequently resolved by SDS–PAGE and visualized by Coomassie Brilliant Blue staining. To improve the specificity of mass spectrometric identification, differential gel bands that were distinctly enriched in the Flag-OTUD6B group compared with the vector control were excised under transmitted light and transferred to nuclease-free microtubes for LC-MS/MS analysis. This selective gel-based approach was adopted instead of direct LC-MS/MS of total co-IP eluates because it effectively reduces nonspecific background and avoids antibody-derived peptides that can obscure the detection of true interactors. Consequently, the gel excision workflow improved the signal-to-noise ratio and enhanced the specificity of substrate identification. For LC-MS/MS, in-gel digested peptides were analyzed using a high-resolution mass spectrometer, and raw spectra were searched with Mascot (v2.3.02) against the UniProt Homo sapiens protein database. The precursor mass tolerance was set to ±10 ppm and the fragment ion tolerance to ±0.02 Da. The false discovery rate (FDR) was controlled at 1%. To ensure high-confidence identification, the LC-MS/MS output was first filtered based on the following stringent criteria: (1) proteins exclusively detected in the Flag-OTUD6B group but absent in the Flag-Vector control; (2) protein FDR confidence classified as “High”; and (3) a sum PEP score ≥ 5, indicating robust peptide evidence. Resulting protein lists were used for downstream interpretation.

### RNA–seq analysis

RNA sequencing of FXR1-silenced samples was performed by Novogene (Beijing, China). Total RNA was extracted; purity was assessed by NanoDrop (A260/A280), and integrity and quantity were determined with an Agilent 2100 Bioanalyzer. Poly(A)+ mRNA was enriched using oligo(dT) magnetic beads, chemically fragmented in the presence of divalent cations, and used for first-strand cDNA synthesis with random primers, followed by second-strand synthesis to generate double-stranded cDNA. Libraries underwent end repair, A-tailing, adapter ligation, size selection to obtain ~200–300 bp inserts, high-fidelity PCR amplification, and purification. Library concentrations were quantified by qPCR, and only libraries with concentrations ≥ 2 nM were advanced. Qualified libraries were pooled according to target yield and sequenced on an Illumina high-throughput platform to generate 150 bp paired-end reads for downstream analysis.
 Raw reads were quality-checked using FastQC and trimmed with Trimmomatic to remove adaptor sequences and low-quality bases. Clean reads were aligned to the human reference genome (GRCh38) using HISAT2 with default parameters. Read counts per gene were generated using featureCounts. Differential expression analysis was carried out using DESeq2, and genes with |log2(fold change)| ≥ 1 and adjusted p-value (Benjamini–Hochberg FDR) < 0.05 were considered significantly differentially expressed. For functional enrichment, ClusterProfiler was employed to perform Gene Ontology (GO) and Kyoto Encyclopedia of Genes and Genomes (KEGG) pathway analyses, using an FDR < 0.05 as the significance threshold. Visualization of heatmaps and enrichment results was performed in R using the packages ggplot2 and pheatmap.

### Glutathione s–transferase (GST) pull–down assays

HEK293T cells were transfected with a Myc-FXR1 expression plasmid, and clarified lysates were prepared 48 h later. Purified GST or GST-OTUD6B (2 μg each) was incubated with equal aliquots of Myc-FXR1-containing lysate for 4 h at 4 °C on a rotating mixer. Glutathione magnetic beads (30 μL) were washed three times with IP wash buffer and added to the reactions for a further 2 h incubation at 4 °C to capture complexes. Beads were collected on a magnetic rack, washed three times with IP wash buffer to reduce nonspecific binding, and bound proteins were eluted in 1× SDS sample buffer by heating at 95–100 °C for 5–10 min. Eluates were analyzed by SDS–PAGE and immunoblotting for Myc to detect FXR1 associated with GST-OTUD6B

### Immunofluorescence staining

Cells were seeded on chambered coverslips and cultured for 24 h to allow adhesion. Cells were rinsed with PBS, fixed with 4% paraformaldehyde for 10–20 min at room temperature (RT), and permeabilized in 0.5% Triton X-100 in PBS for 20 min. Nonspecific binding was blocked with goat serum for 30 min at RT after three PBS washes. Primary antibodies against OTUD6B and FXR1 (from different host species) were diluted in blocking buffer and incubated overnight at 4 °C in the dark. The next day, cells were washed and incubated with species-appropriate, fluorophore-conjugated secondary antibodies for 1 h at 37 °C protected from light. Nuclei were counterstained with DAPI for 5–10 min, followed by PBS washes. Coverslips were mounted with antifade medium, stored at 4 °C protected from light, and imaged on a confocal fluorescence microscope.

### *In vivo* deubiquitination assay

At 72 h after transfection with the indicated plasmids, cells were lysed in ice-cold immunoprecipitation (IP) buffer, and lysates were clarified by centrifugation. For immunocapture, 2 mg of total protein was incubated with the indicated primary antibody at 4 °C overnight. Protein A/G magnetic beads were then added and incubated for 2 h at 4 °C to isolate immune complexes. Beads were collected on a magnetic rack, washed three times with IP wash buffer to reduce nonspecific binding, and bound proteins were eluted by boiling in 1× SDS sample buffer at 95–100 °C for 5–10 min. Eluates were resolved by SDS–PAGE and analyzed by immunoblotting with the indicated antibodies.

### Ubiquitin Mutant Assay Design

To determine the ubiquitin linkage specificity of OTUD6B, a panel of lysine-mutant ubiquitin constructs (K6, K11, K27, K29, K33, K48, and K63) was used. In each mutant, only one lysine residue was retained, while all other lysine residues were substituted with arginine (R). For example, in the K6-Ub construct, only the lysine at position 6 remains intact, whereas all other lysines (K11, K27, K29, K33, K48, and K63) are mutated to arginine. This design ensures that polyubiquitin chains formed in cells are exclusively linked through the specified lysine residue, thereby allowing precise identification of the linkage type recognized or cleaved by OTUD6B. HEK-293T cells were co-transfected with Myc-FXR1, Flag-OTUD6B, and each ubiquitin mutant plasmid, followed by treatment with 25 μM MG132 for 6 h. Co-immunoprecipitation was performed using anti-Myc antibody, and ubiquitination patterns were analyzed by immunoblotting. The comparative analysis of ubiquitination among different lysine mutants enabled the determination of the specific ubiquitin linkage preference of OTUD6B.

### *In vitro* deubiquitination assay

HEK293T cells were co-transfected with Myc-FXR1 and HA-Ub expression plasmids for 48 h. Cells were then treated with 25 µM MG132 for 6 h to accumulate ubiquitinated FXR1. Subsequently, cells were lysed and ubiquitinated Myc-FXR1 was immunoprecipitated using anti-Myc affinity beads. The immunoprecipitates were washed three times with ubiquitination wash buffer and eluted in BC100 buffer with 3×Myc peptide. The eluted ubiquitinated FXR1 was incubated with 200 ng of recombinant GST-OTUD6B or the catalytically inactive mutant GST-OTUD6B^C158S^ in deubiquitination buffer (50 mM Tris-HCl, pH 8.0; 50 mM NaCl; 1 mM EDTA; 10 mM DTT; 5% glycerol) for 2 h at 37 °C. The ubiquitination status of FXR1 was then analyzed by Western blotting.

### RNA immunoprecipitation (RIP) assays

RIP assays were conducted using the Magna RIP Kit (17-701; EMD Millipore, Billerica, MA, USA) to assess FXR1-associated RNA complexes in HCT116 cells. Cells at 80–90% confluence were lysed in RIP buffer supplemented with RNase inhibitor and PMSF, and lysates were incubated overnight at 4 °C with protein A/G magnetic beads conjugated to either anti-FXR1 antibody or IgG control. After washing, RNA–protein complexes were digested with proteinase K, and total RNA was extracted using TRIzol followed by ethanol precipitation. Purified RNA was subjected to transcriptome sequencing on an Illumina NextSeq 500 platform (Aksomics, Shanghai, China) and validated by qPCR.

### Assays for colony formation

Colony-formation assays were performed to evaluate the proliferative capacity of CRC cells following genetic or pharmacologic perturbation. HCT116 and SW480 cells were harvested, enumerated, and seeded in six-well plates at 1 × 10^3^ and 1.5 × 10^3^ cells per well, respectively. Cells were maintained in complete medium with medium changes every 3 days for 7–10 days until discrete colonies were visible. Colonies were rinsed three times with PBS, fixed with 4% paraformaldehyde for 20 min, and stained with crystal violet for 20 min at room temperature. Excess dye was removed with distilled water, plates were air-dried, and colonies were imaged for quantitative analysis.

### Transwell assay

Cells subjected to genetic or pharmacologic perturbations were harvested, resuspended in serum-free medium, and enumerated. Transwell inserts were placed in 24–well plates with 600 μL medium containing 20% FBS in the lower chamber. For the upper chamber, 300 μL of cell suspension was added at 1 × 10^5^ HCT116 cells or 2 × 10^5^ SW480 cells per insert. For invasion assays, inserts were pre-coated on the upper surface with 30 μL Matrigel and allowed to polymerize for 2 h before seeding. After 48 h of incubation, inserts were rinsed with PBS, fixed with 4% paraformaldehyde for 20 min, and stained with crystal violet for 20 min. Nonmigrated/noninvaded cells on the upper membrane surface were gently removed with a cotton swab. Membranes were rinsed with water, air-dried, and imaged by light microscopy; migrated/invaded cells were quantified from five random fields per insert for statistical analysis.

### mRNA stability assay

Cells were seeded into six–well plates and cultured until reaching 70–80% confluence. Transcription was inhibited by adding actinomycin D (HY-17559, MCE) at a final concentration of 5 µg/mL, and cells were collected at 0, 2, 4, and 6 h. Total RNA was extracted, and mRNA abundance was quantified by RT–qPCR using gene-specific primers. GAPDH served as the internal control for normalization of transcript levels.

**Protein stability assay**

Cells were seeded into six–well plates until reaching 70–80% confluence. Protein synthesis was inhibited by adding cycloheximide (CHX, C112766, Aladdin Biotech, Shanghai, China) to a final concentration of 20 µM, and cells were harvested at 0, 2, 4, and 6 h. Total protein was extracted at each time point and subjected to Western blot analysis to assess protein stability.
