# Supplementary material for: OTUD6B-mediated K48 deubiquitination of FXR1 forms a positive feedback loop activating MEK2/ERK signaling in colorectal cancer liver metastasis
Source: Cell Death Dis. 2026 Apr 29;17(1):572. doi: 10.1038/s41419-026-08812-z (PMC13269552; doi:10.1038/s41419-026-08812-z)

Figure 1

1F OTUD6B_#1-#6

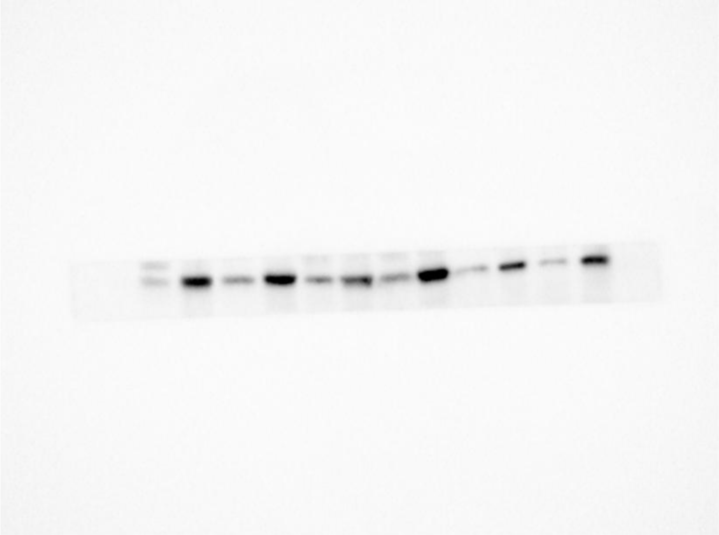

1F β-actin _#1-#6

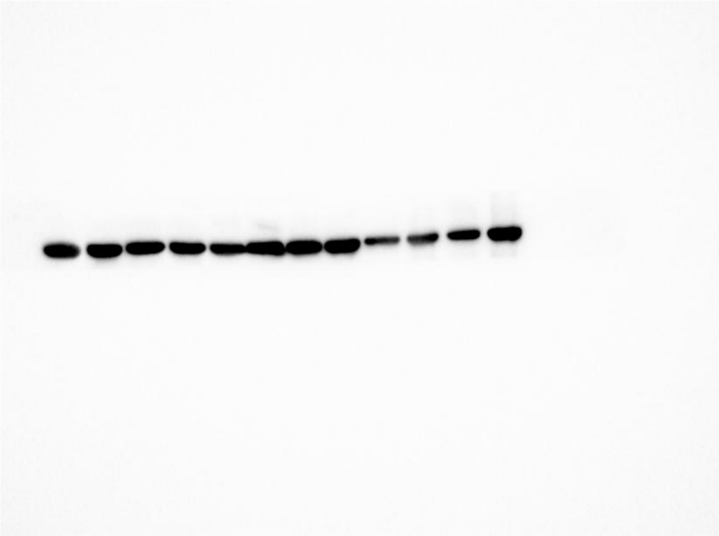

1F OTUD6B_#7-#12

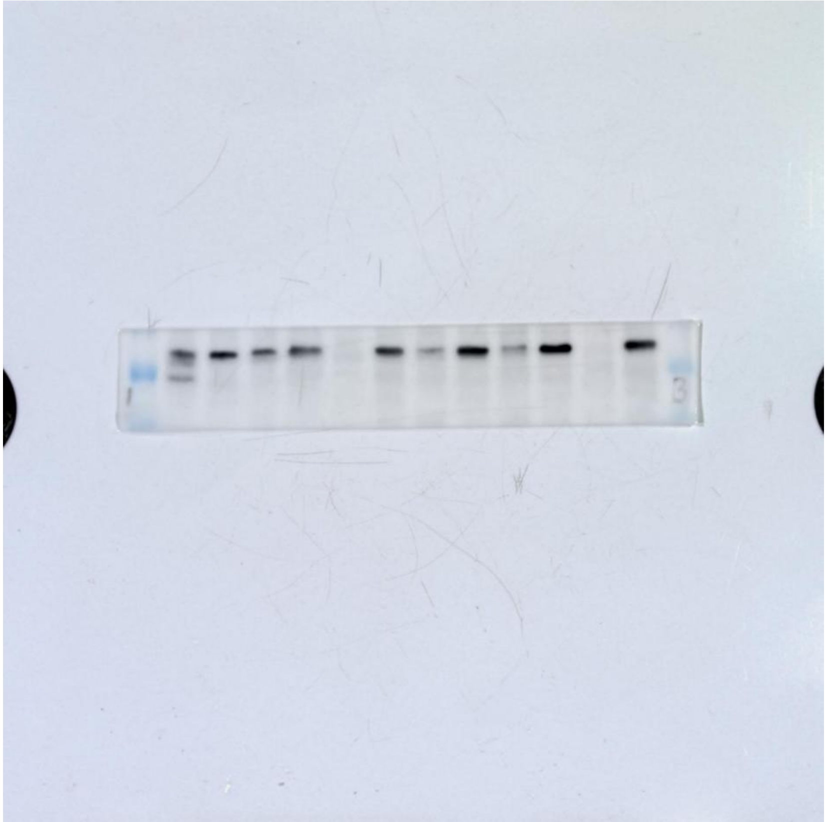

1F β-actin _#7-#12

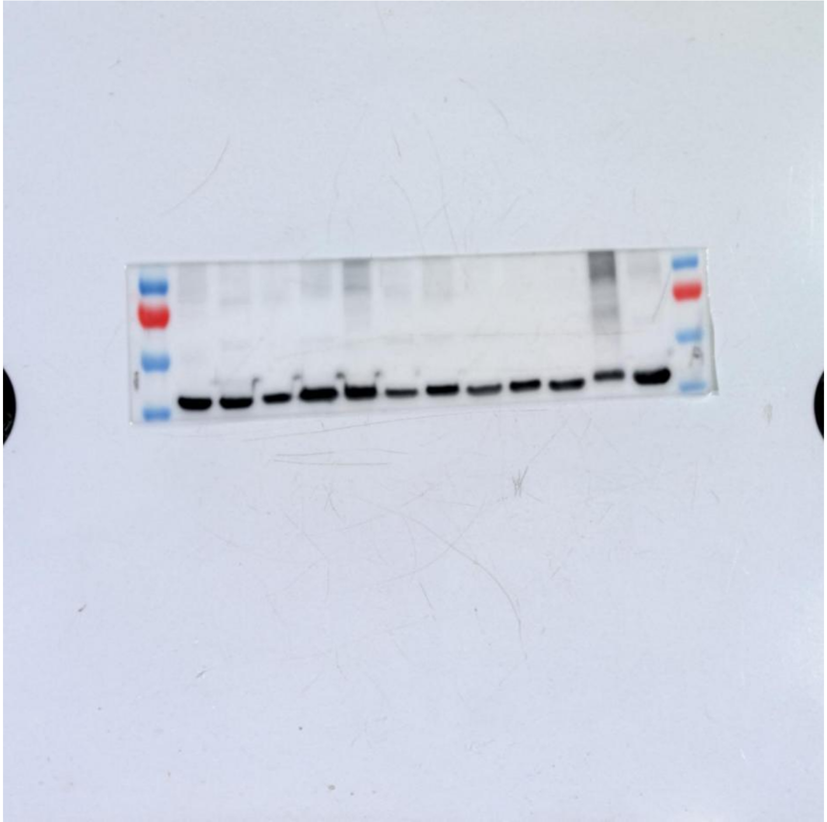

1F OTUD6B_#13-#18

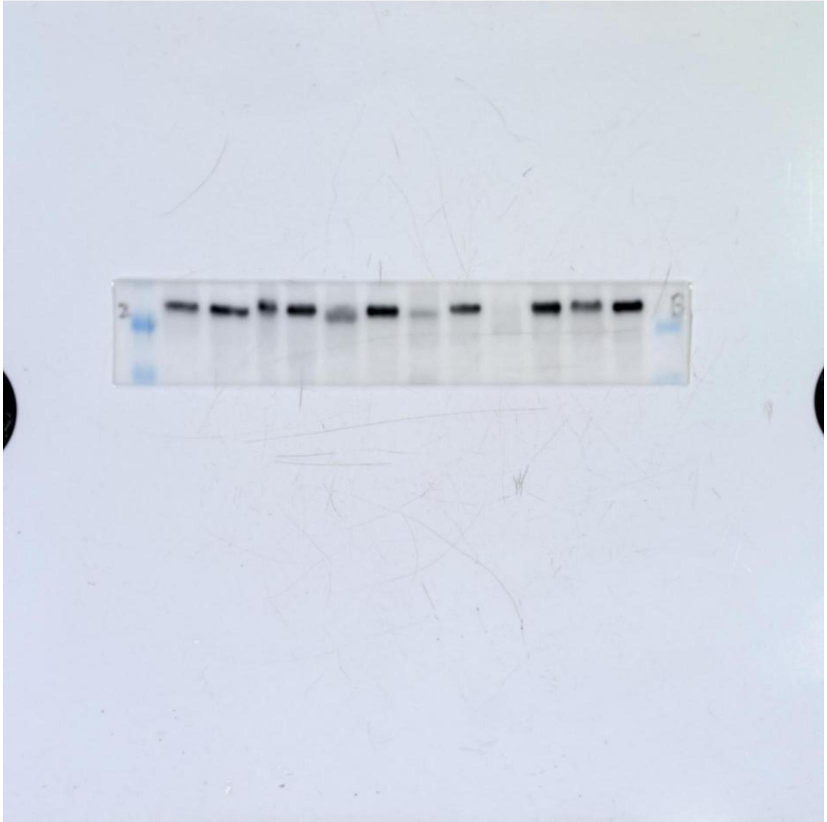

1F β-actin _#13-#18

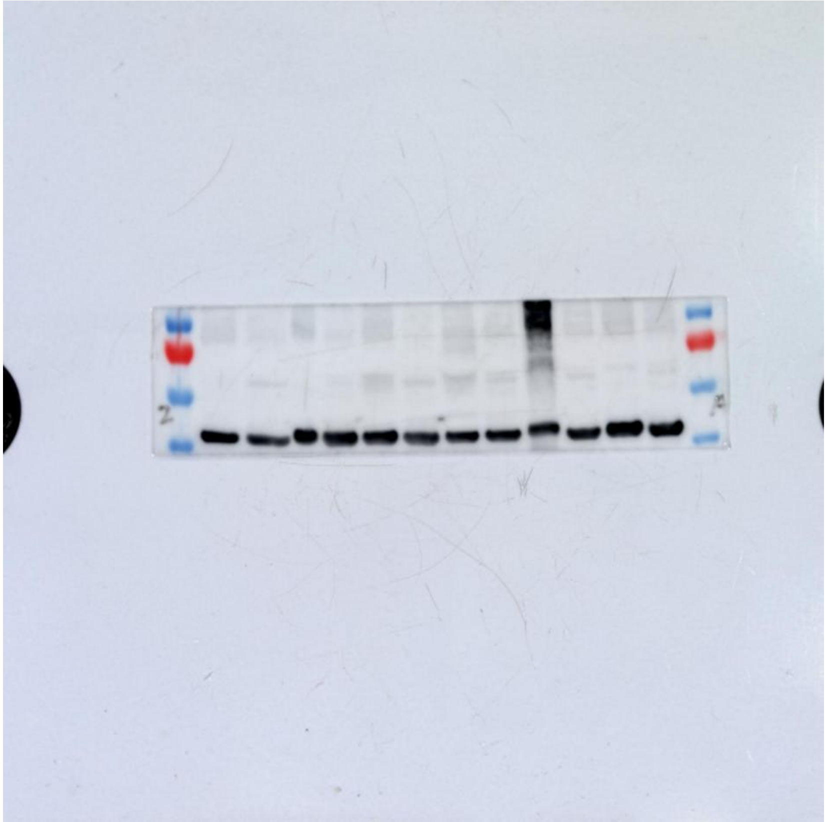

1F OTUD6B_#19-#22

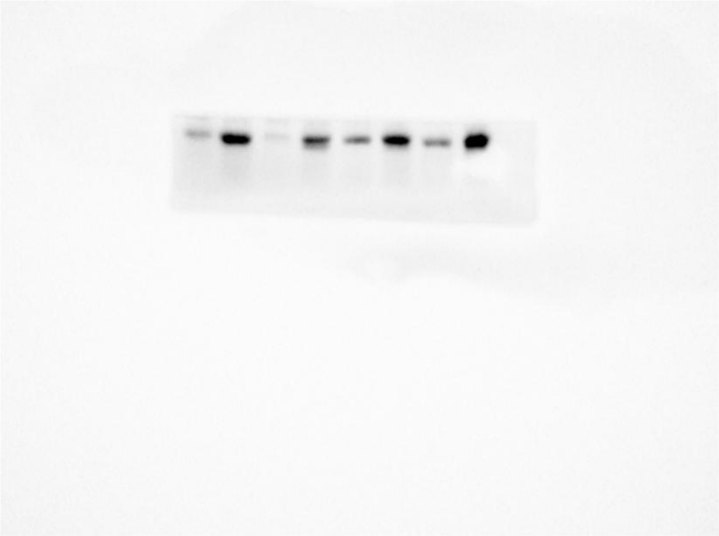

1F β-actin _#19-#22

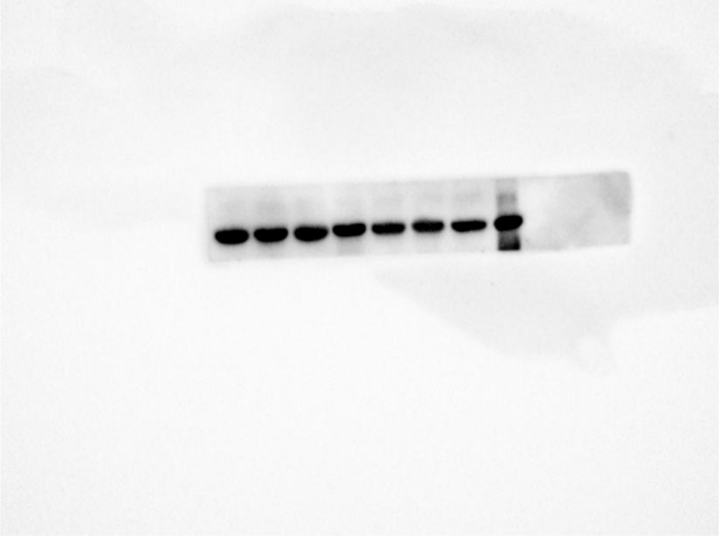

1G OTUD6B

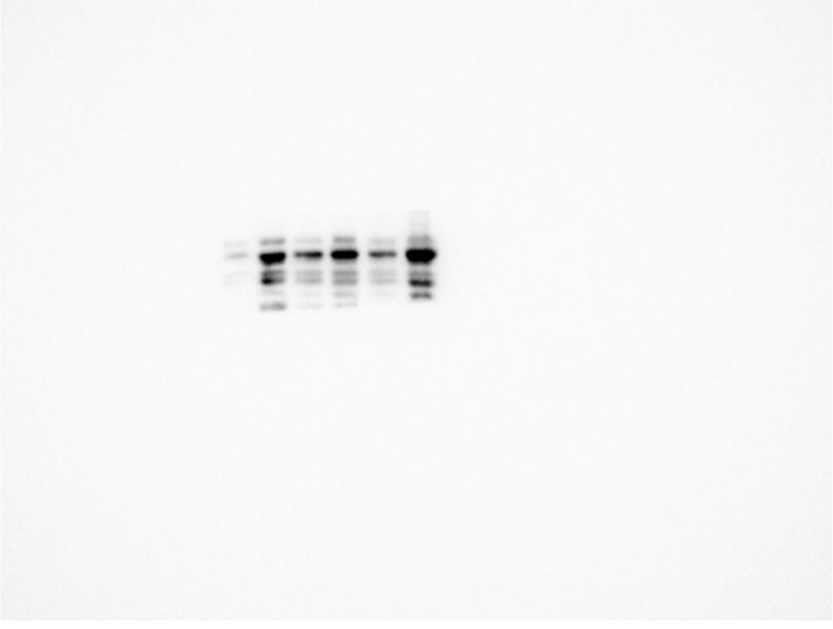

1G β-actin

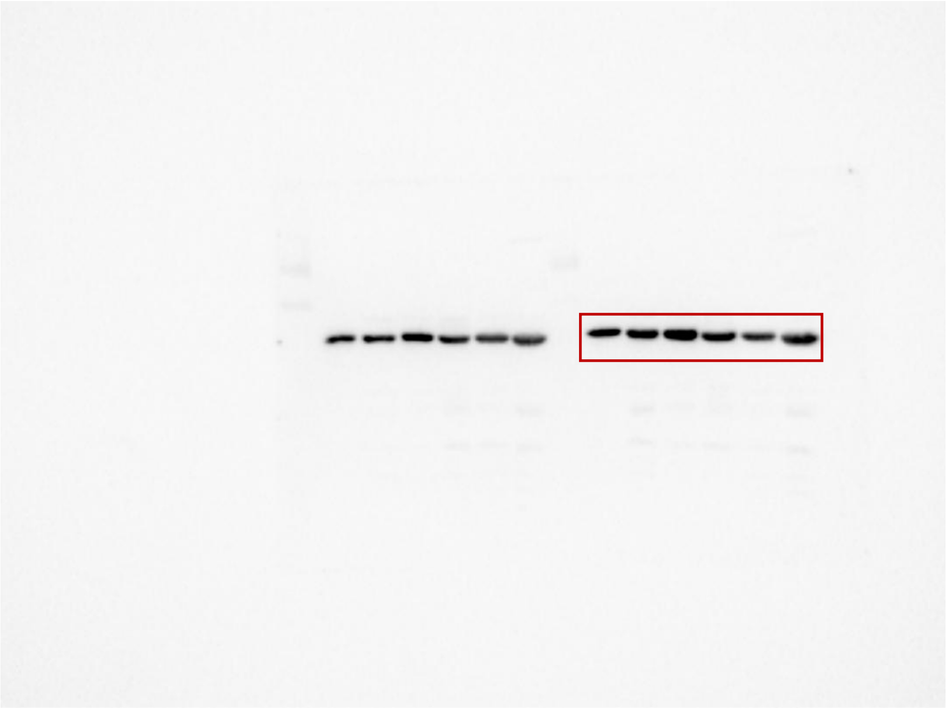

Figure 2

2A OTUD6B HCT116

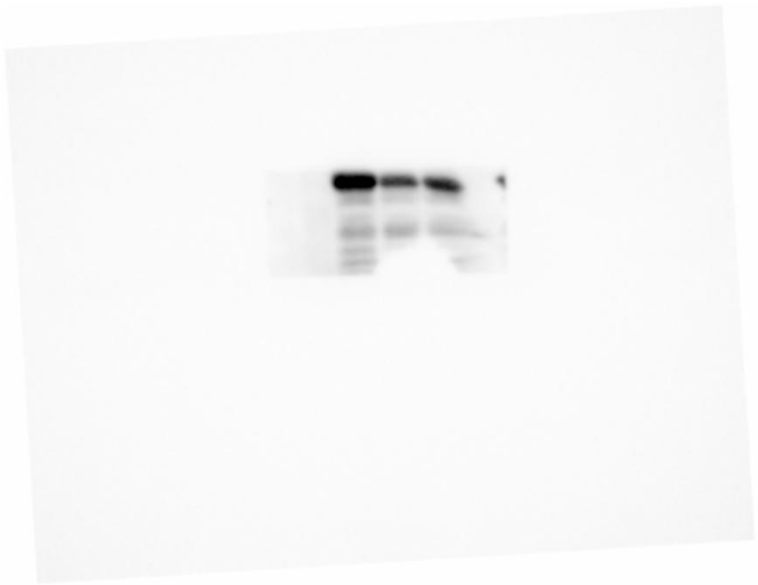

2A β-actin HCT116

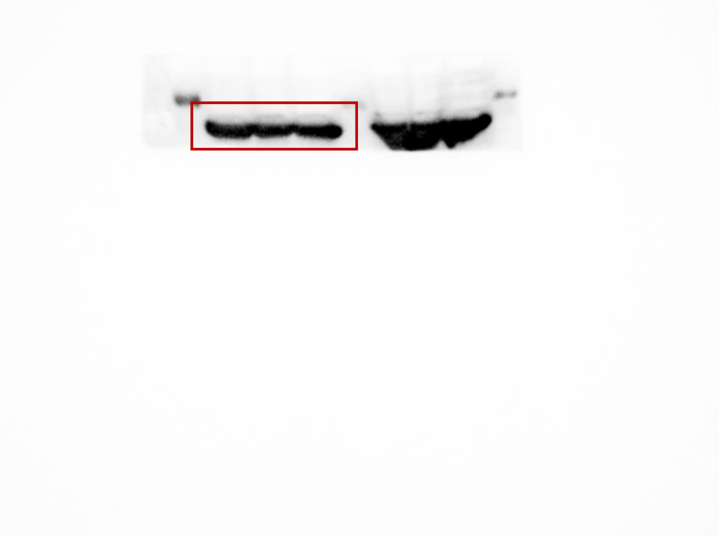

2A OTUD6B SW480

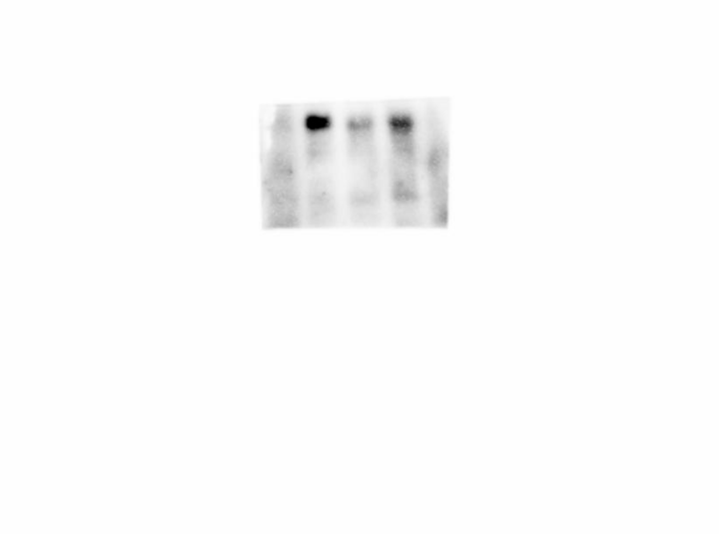

2A β-actin SW480

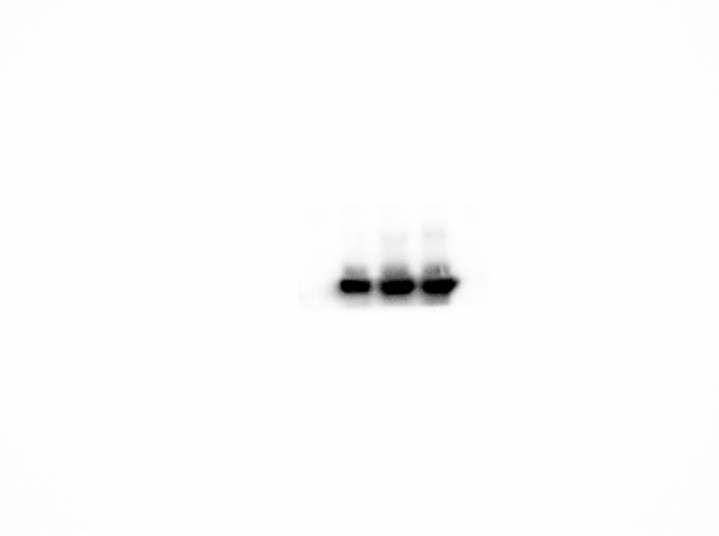

2B Flag HCT116

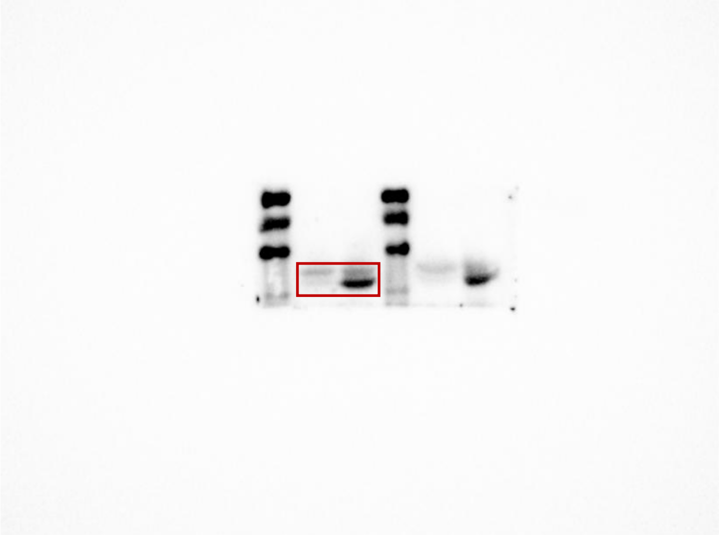

2B β-actin HCT116

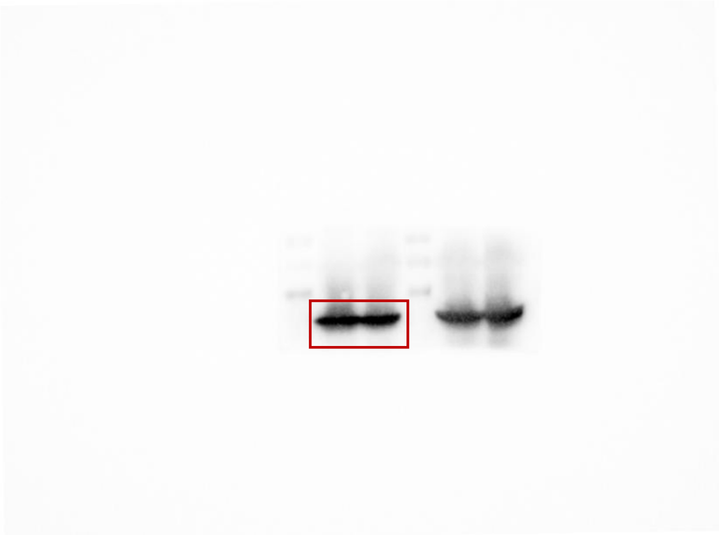

2B Flag SW480

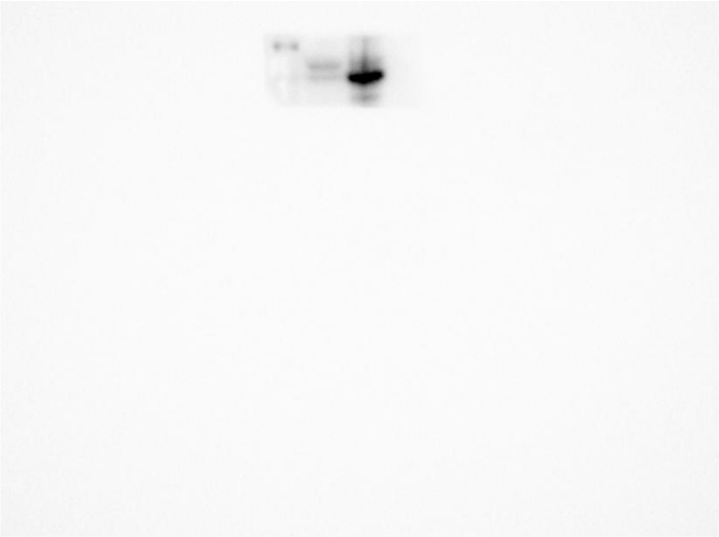

2B β-actin SW480

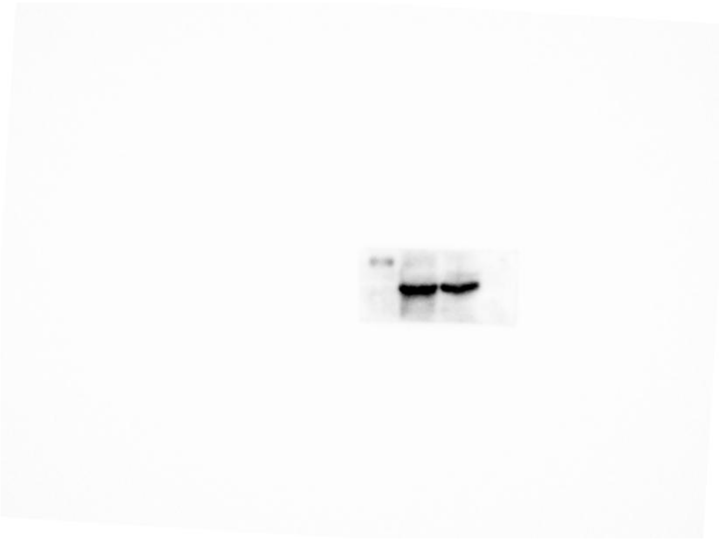

Figure 3

3D OTUD6B HCT116

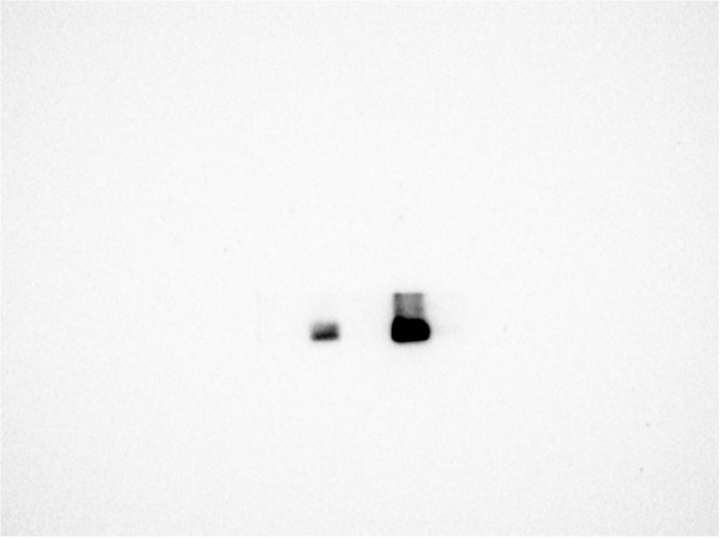

3D FXR1 HCT116

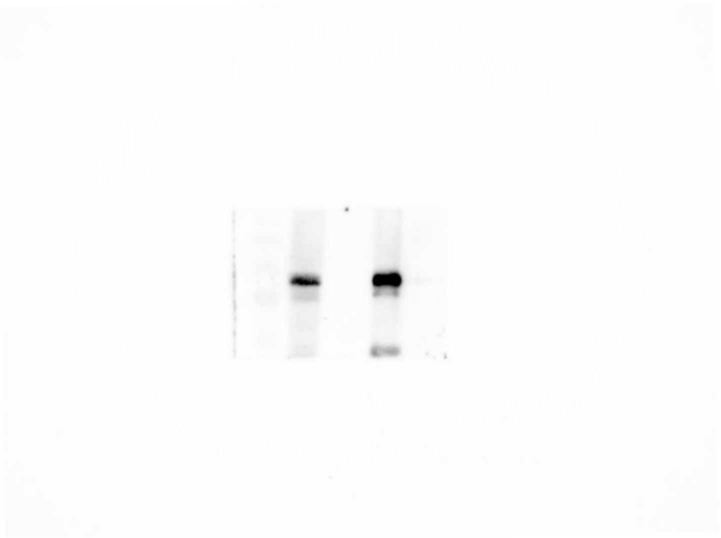

3D OTUD6B SW480

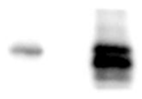

3D FXR1 SW480

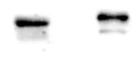

3E OTUD6B HCT116

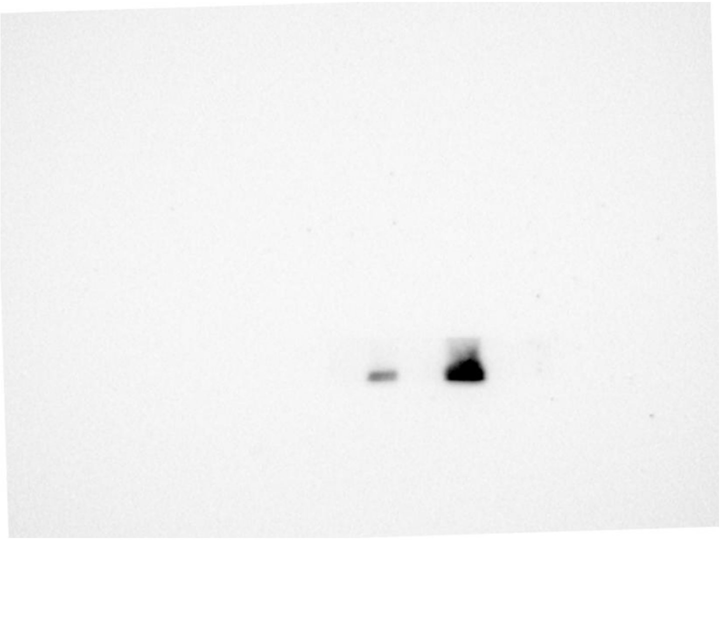

3E FXR1 HCT116

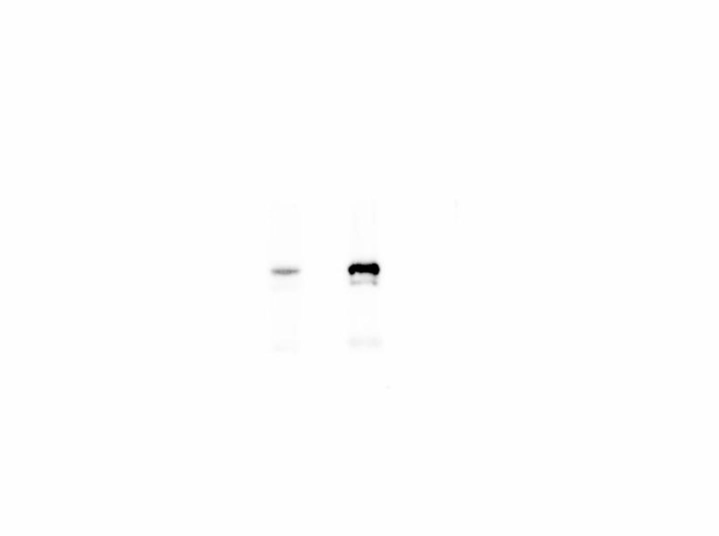

3E OTUD6B SW480

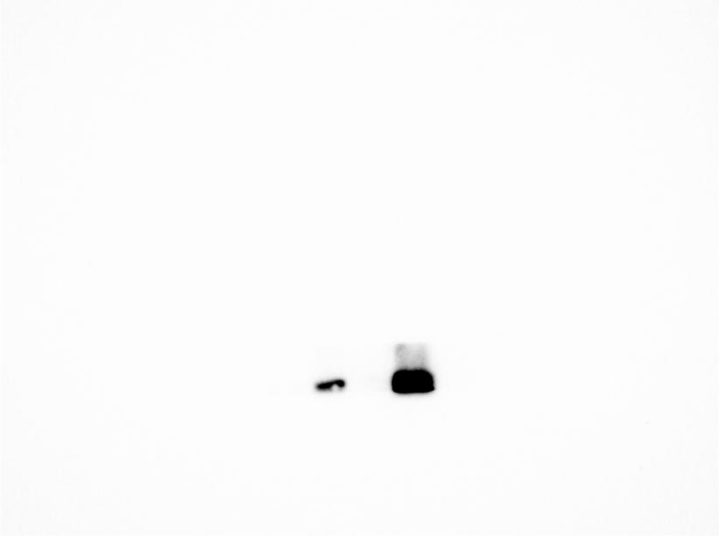

3E FXR1 SW480

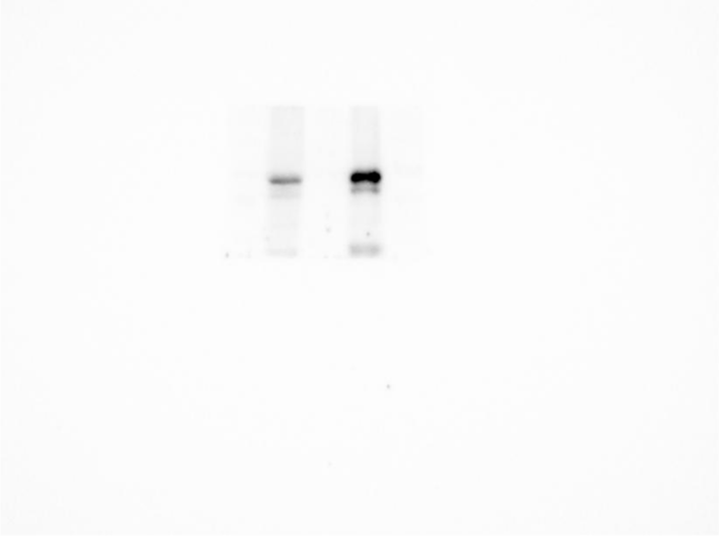

3F Flag up panel

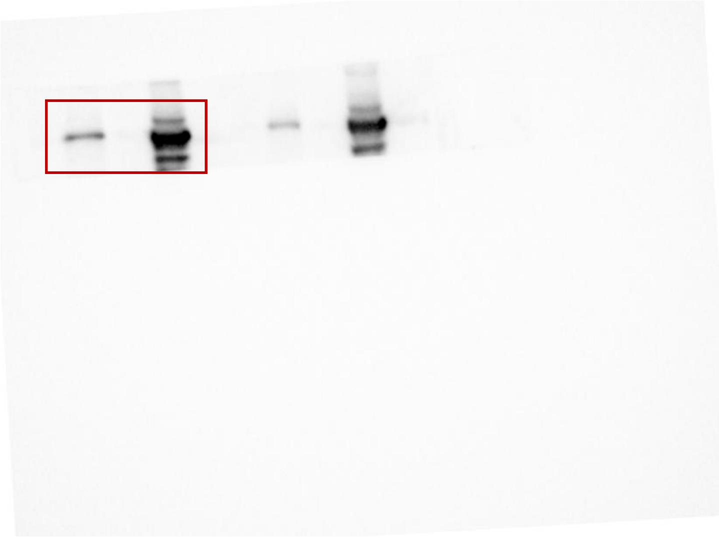

3F Myc up panel

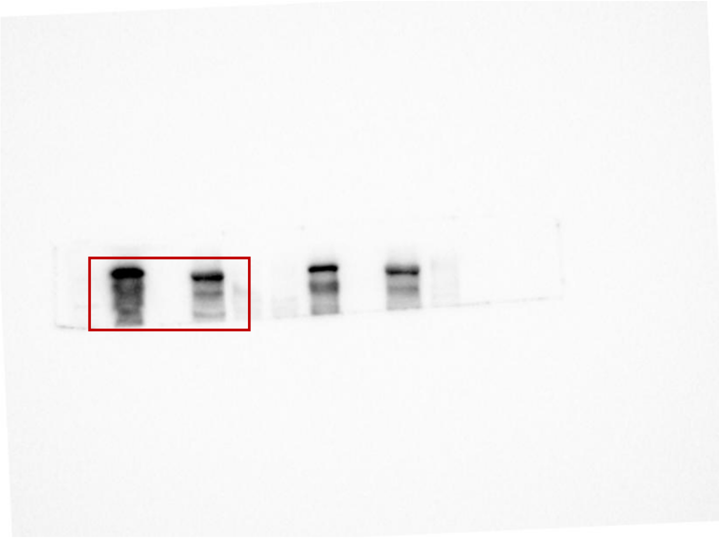

3F Flag down panel

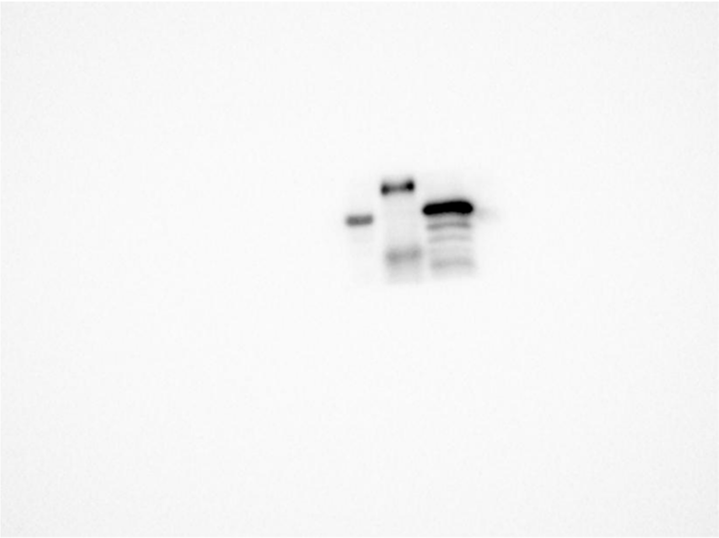

3F Myc down panel

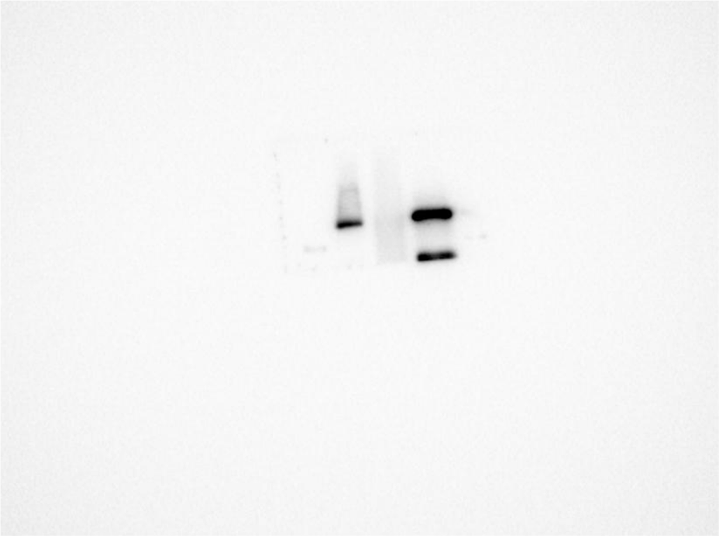

3G IP Myc

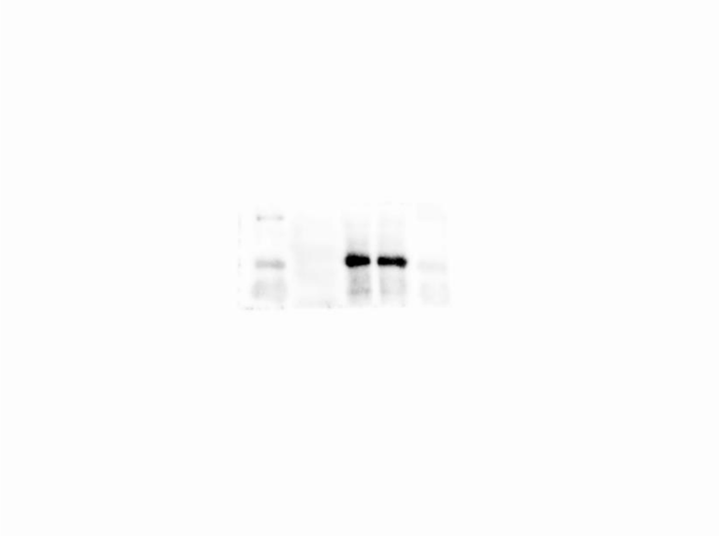

3G IP Flag

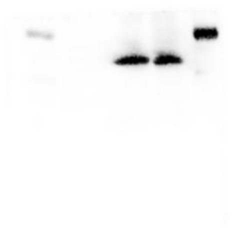

3G Input Myc

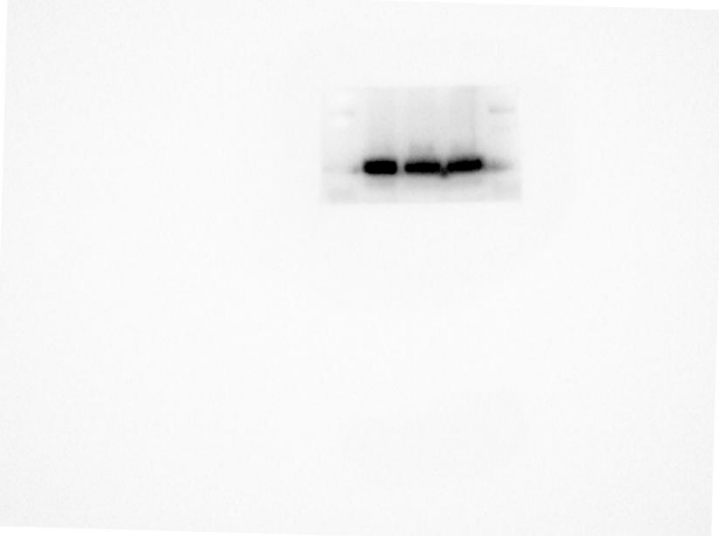

3G Input Flag

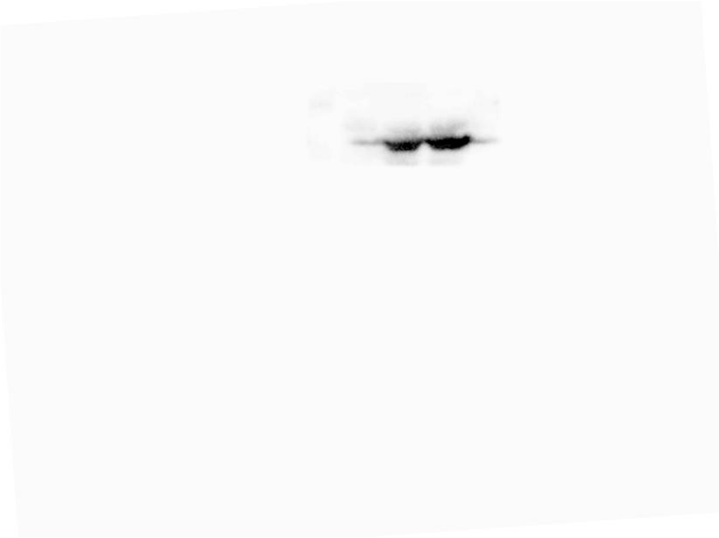

3H Myc-FXR1

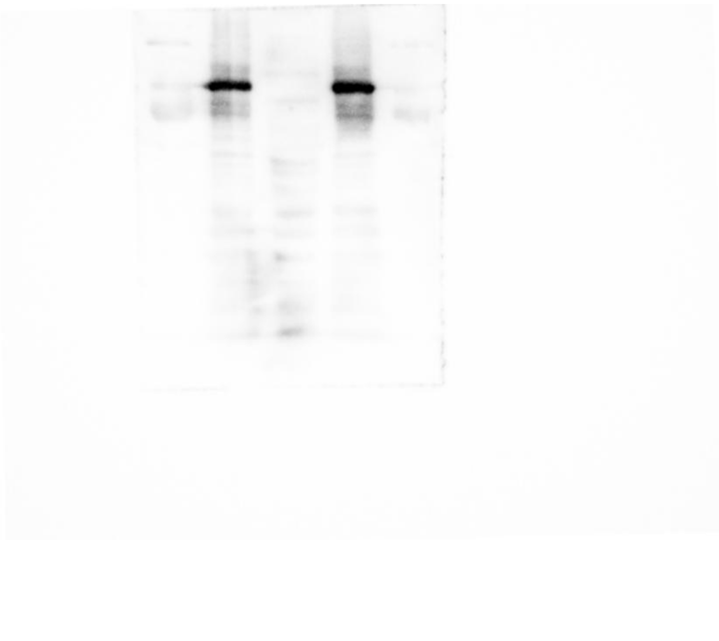

3L Myc

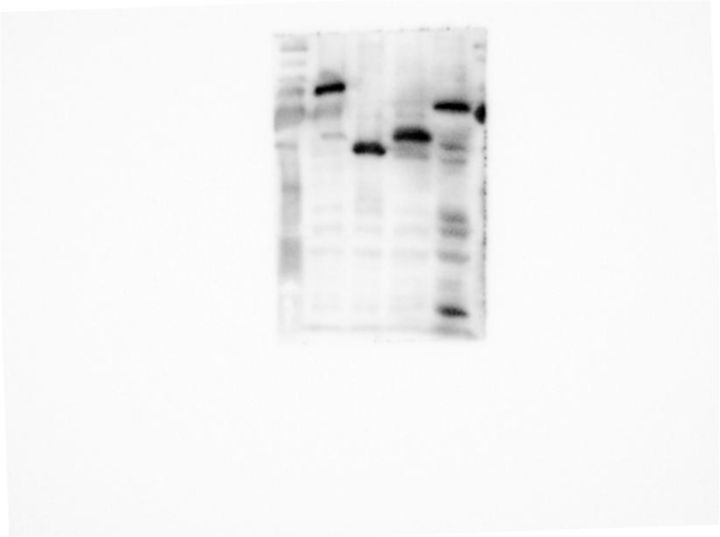

3L Flag

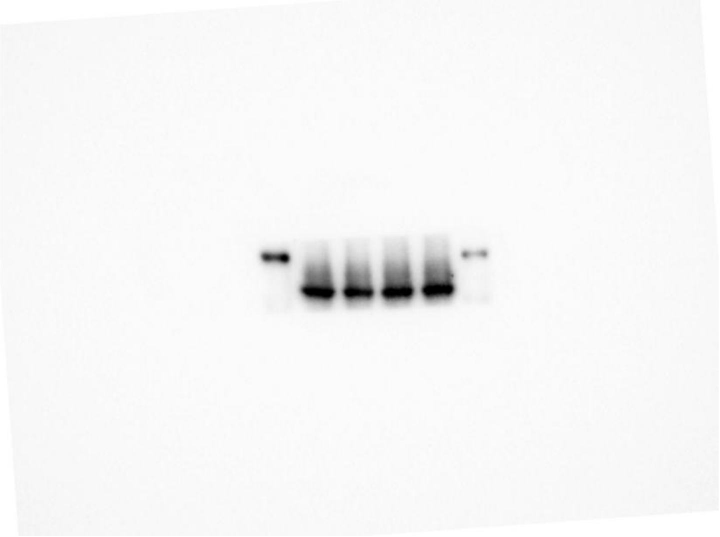

3L GAPDH

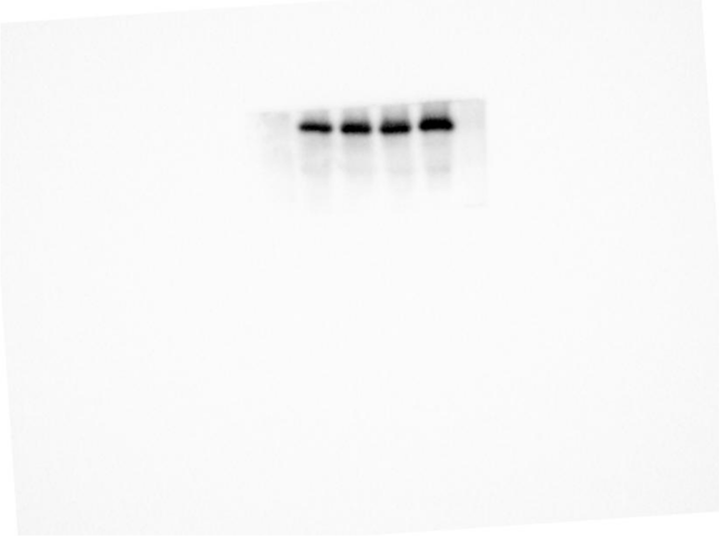

3M Flag

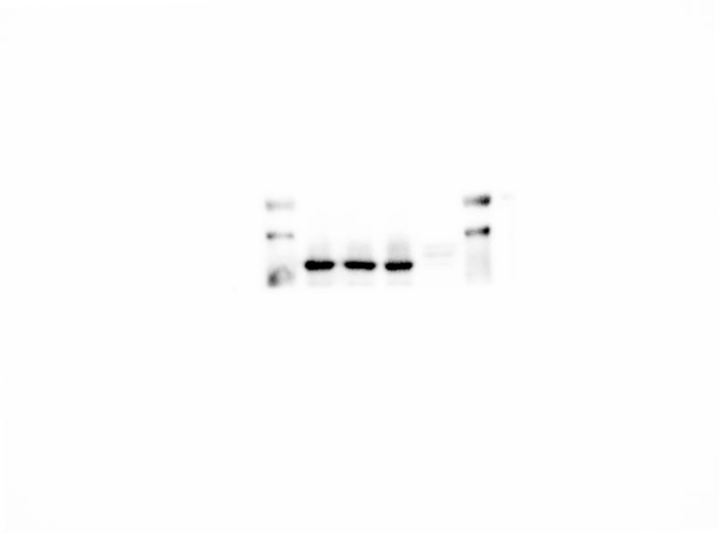

3M Myc

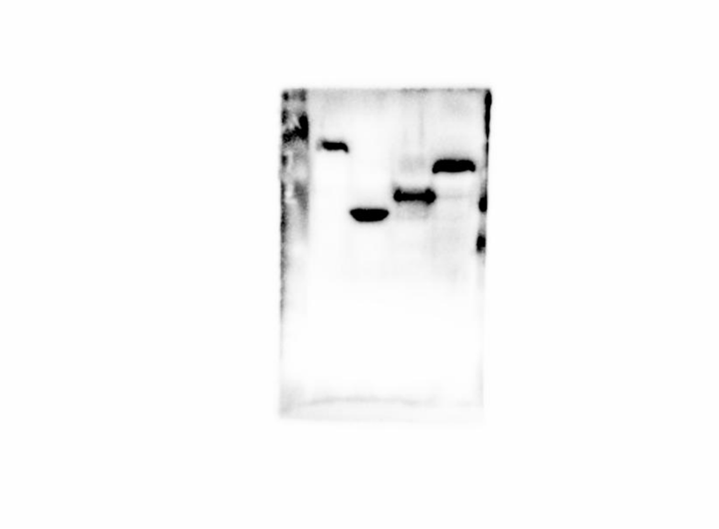

3N Myc

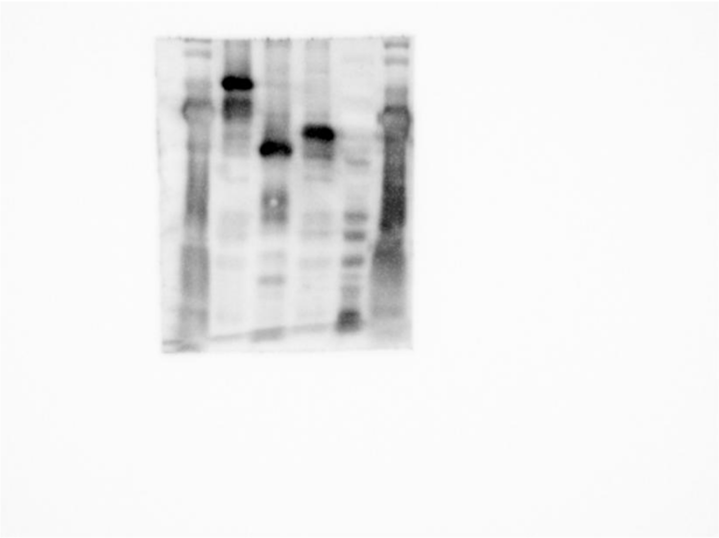

3N Flag

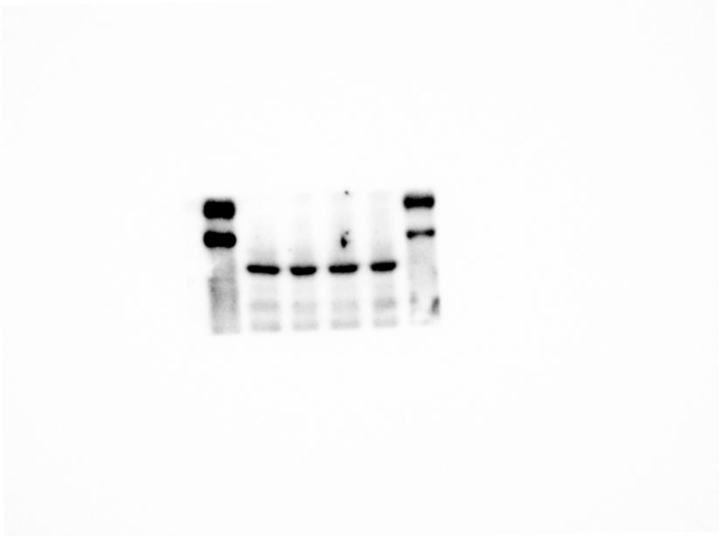

3O Flag

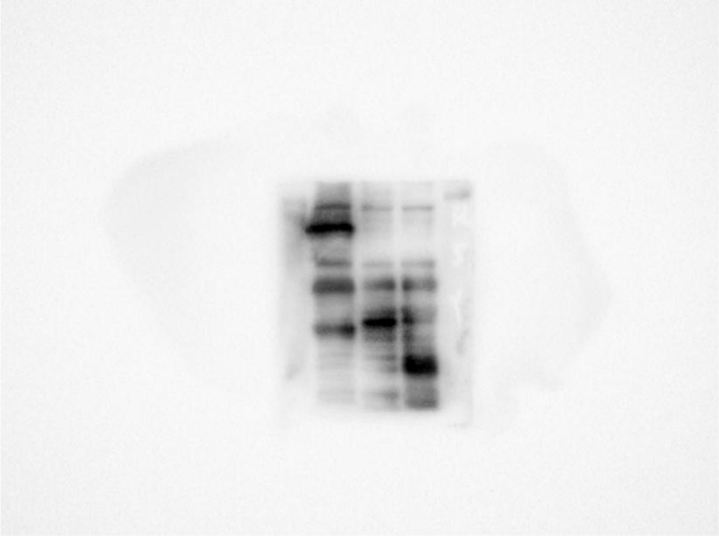

3O Myc

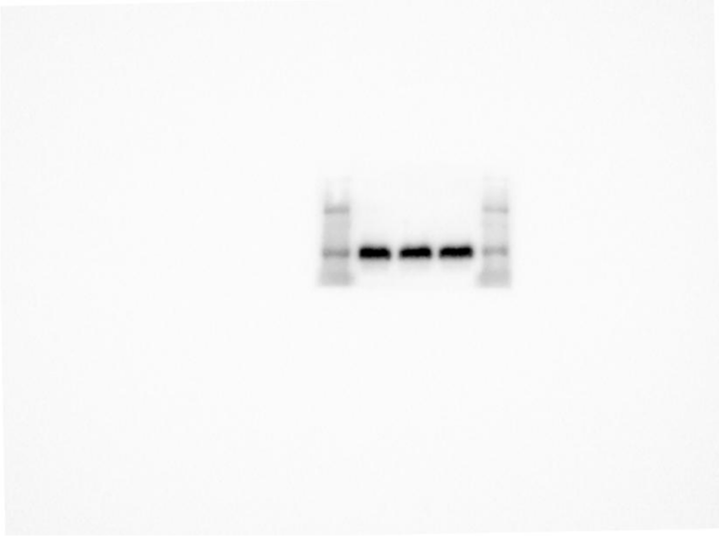

3O GAPDH

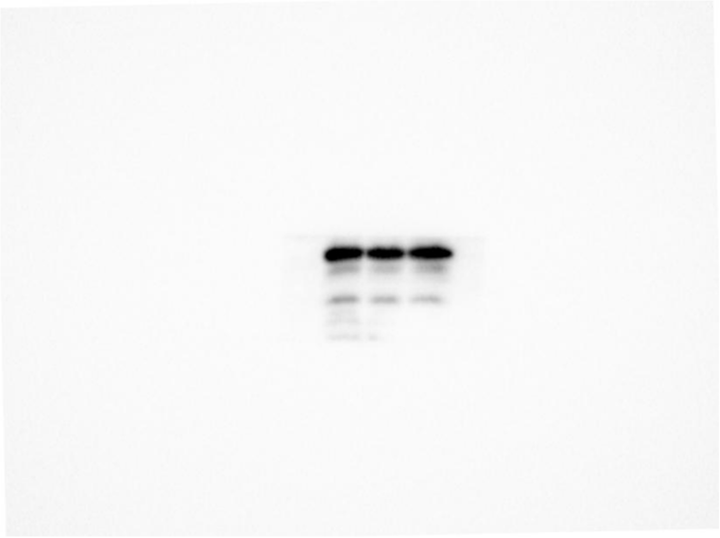

3P Flag

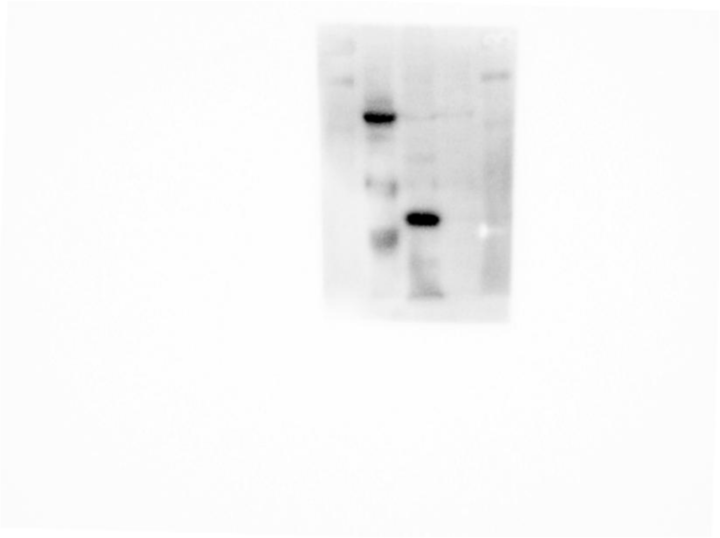

3P Myc

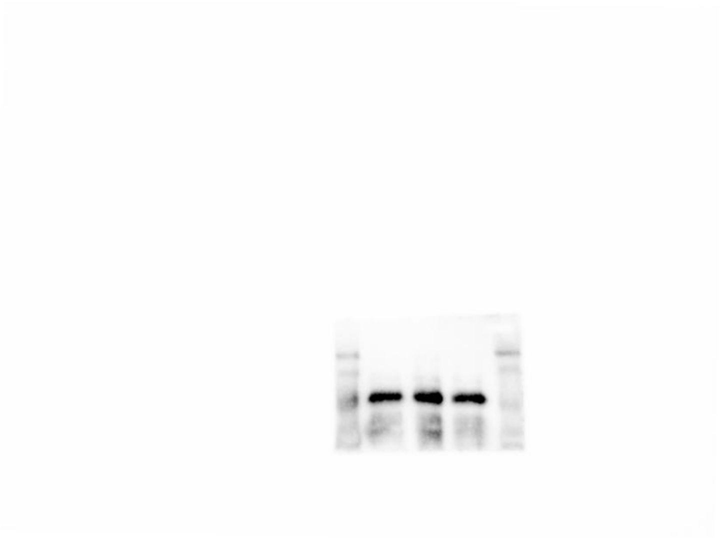

3Q Myc

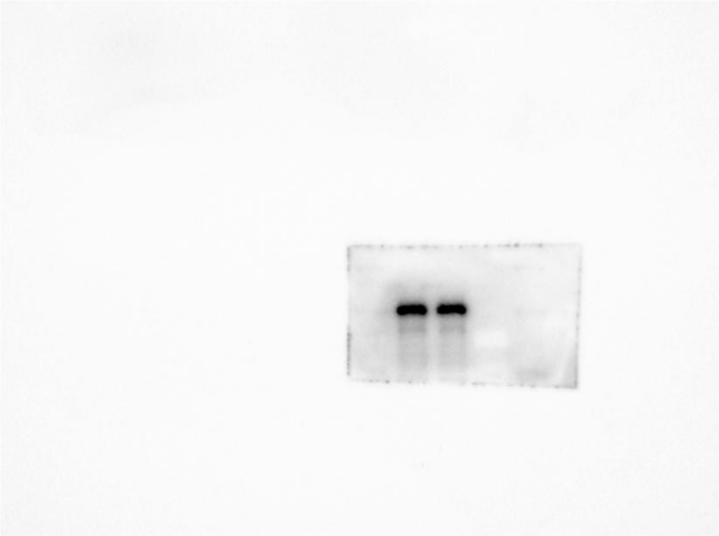

3Q Flag

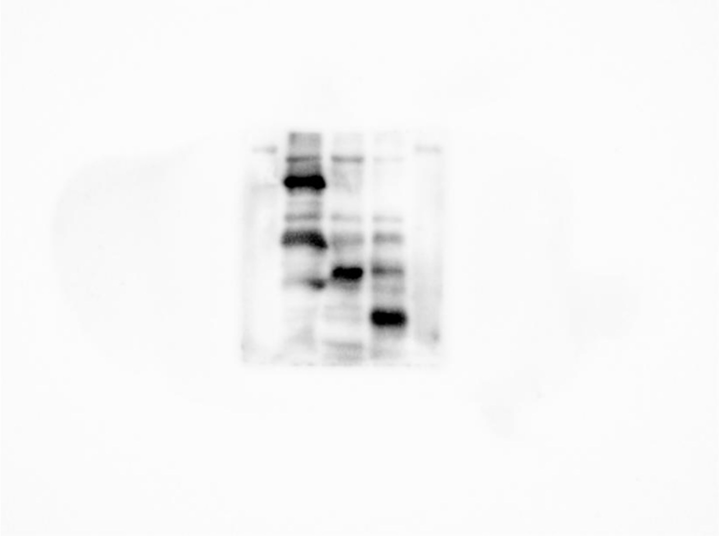

Figure 4

4A FXR1 HCT116

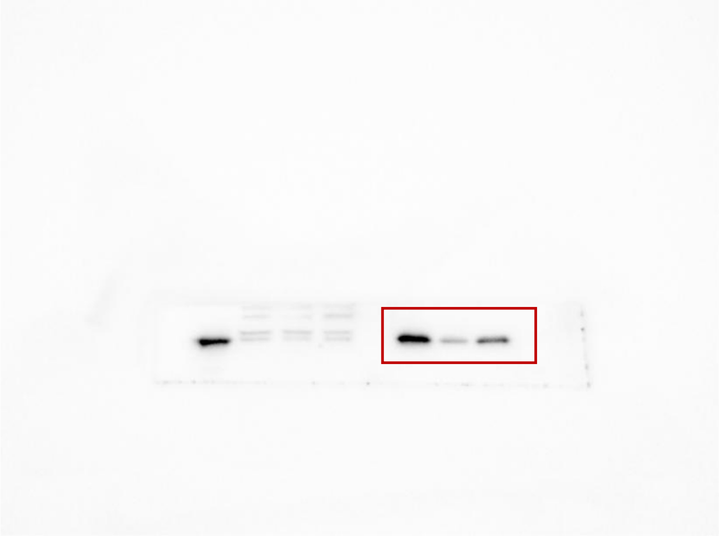

4A OTUD6B HCT116

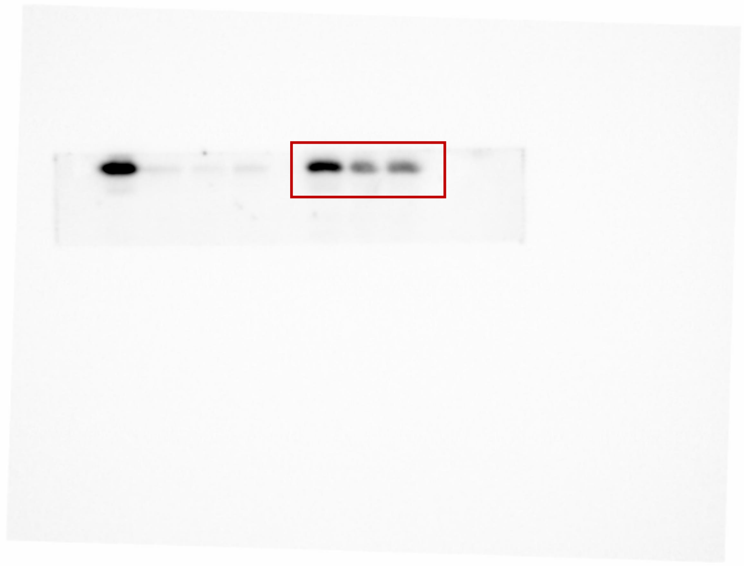

4A β-actin HCT116

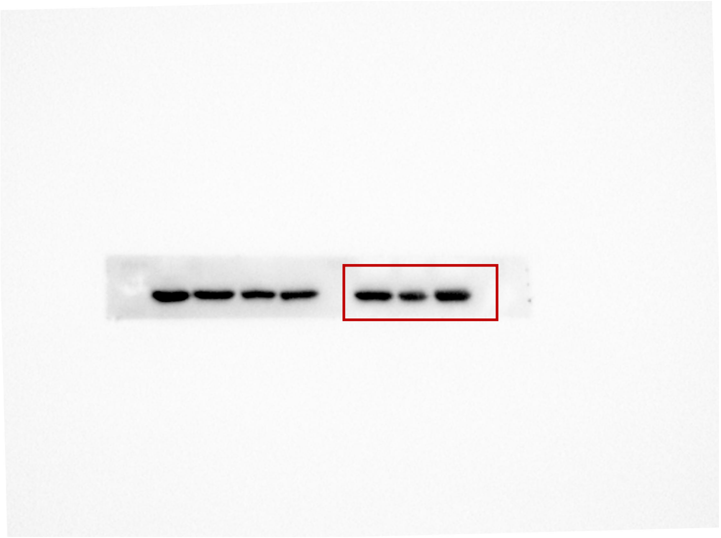

4A FXR1 SW480

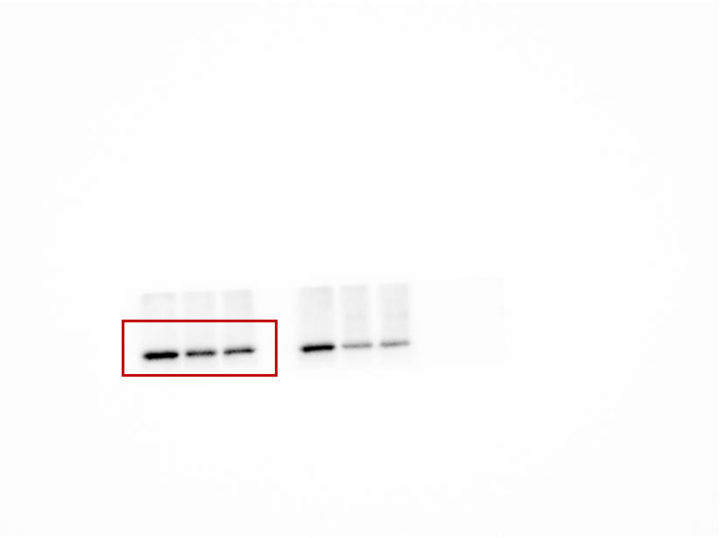

4A OTUD6B　SW480

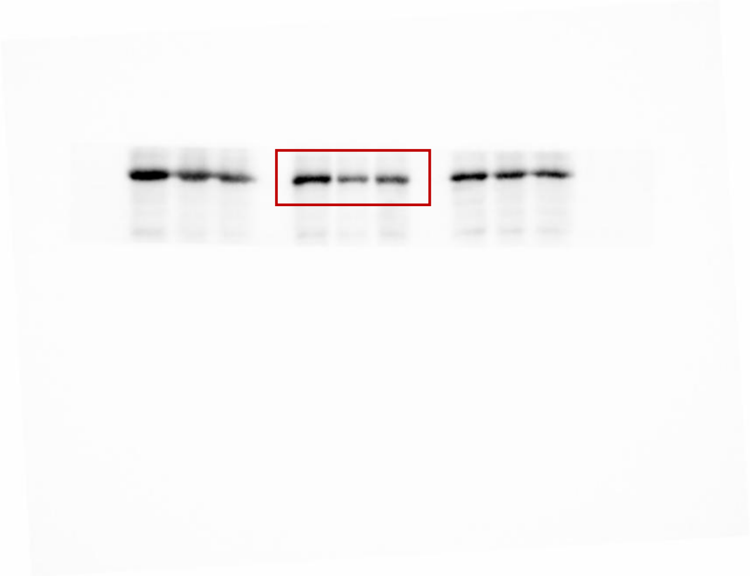

4A β-actin　SW480

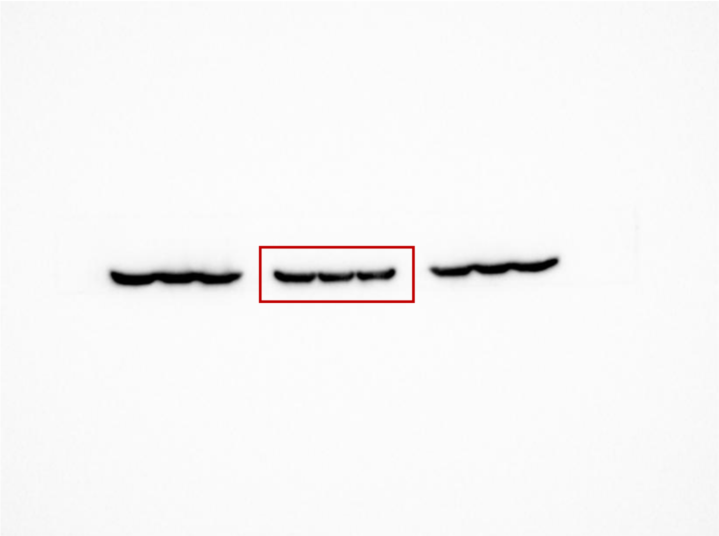

4B FXR1

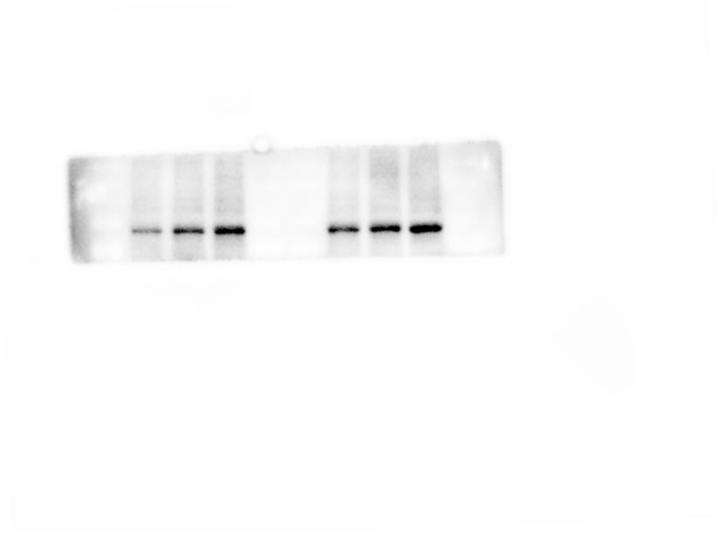

4B Flag

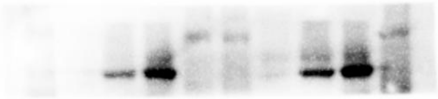

4B β-actin

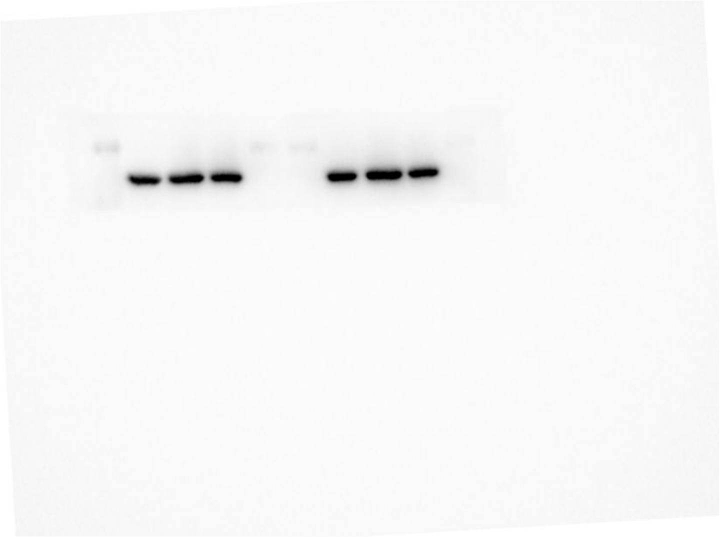

4C FXR1

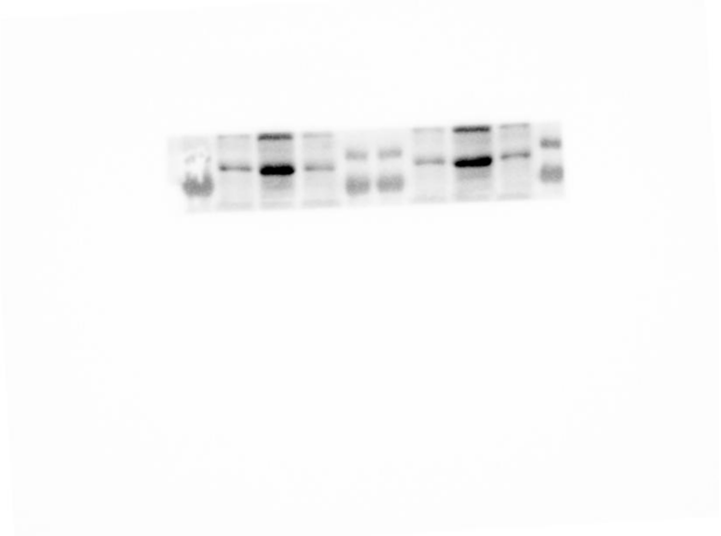

4C Flag

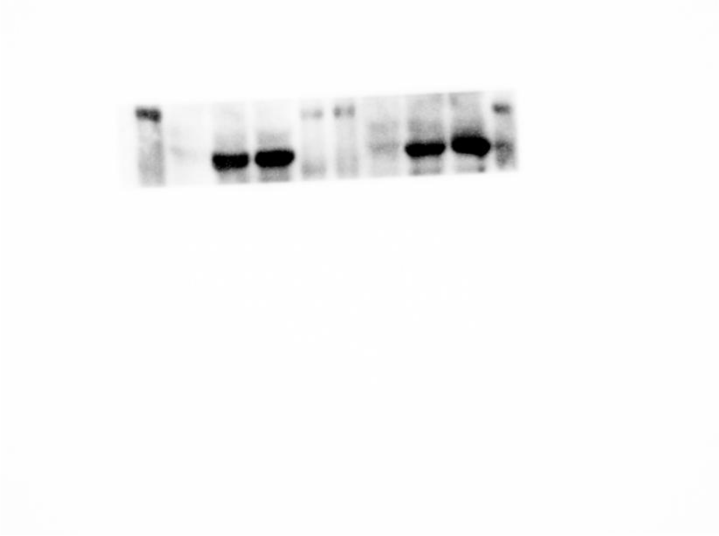

4C β-actin

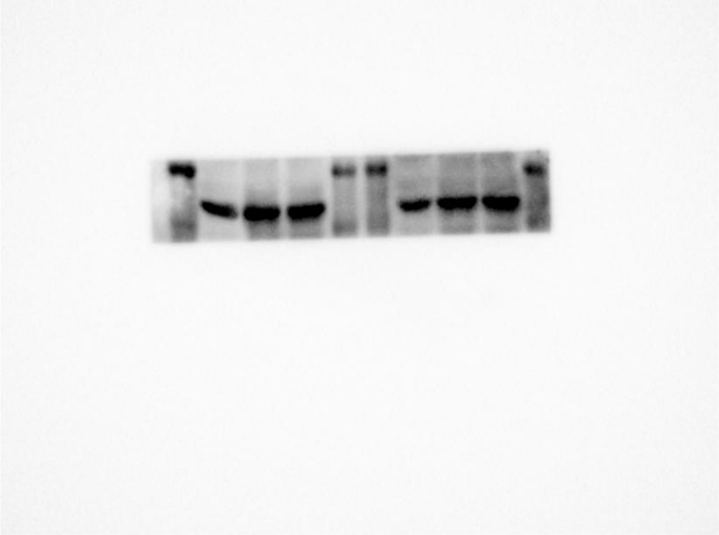

4D FXR1

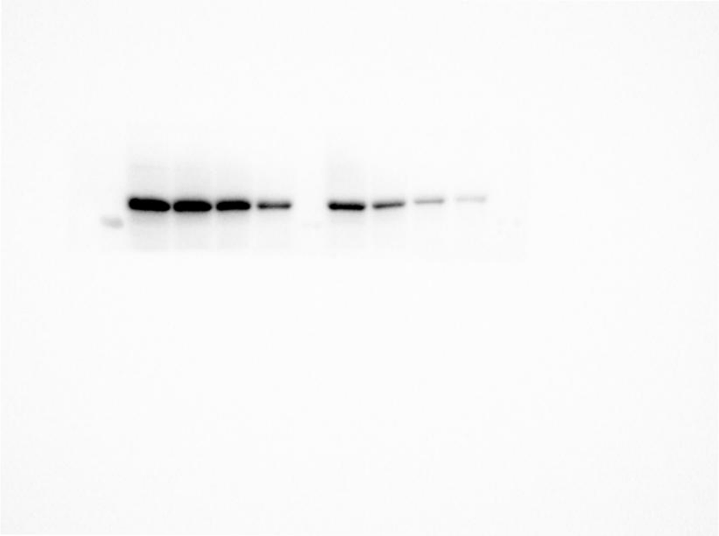

4D OTUD6B

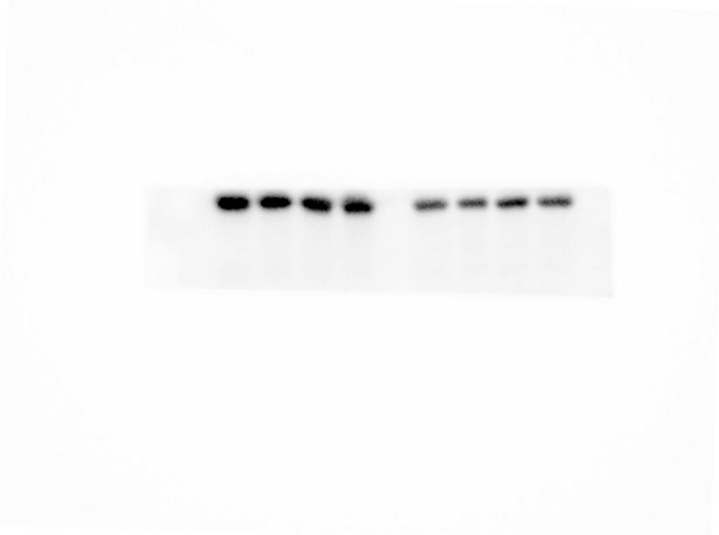

4D β-actin

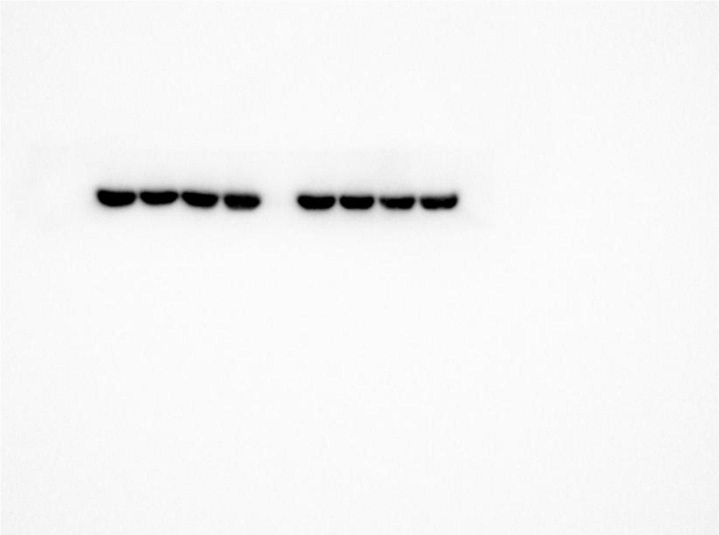

4E FXR1

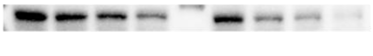

4E OTUD6B

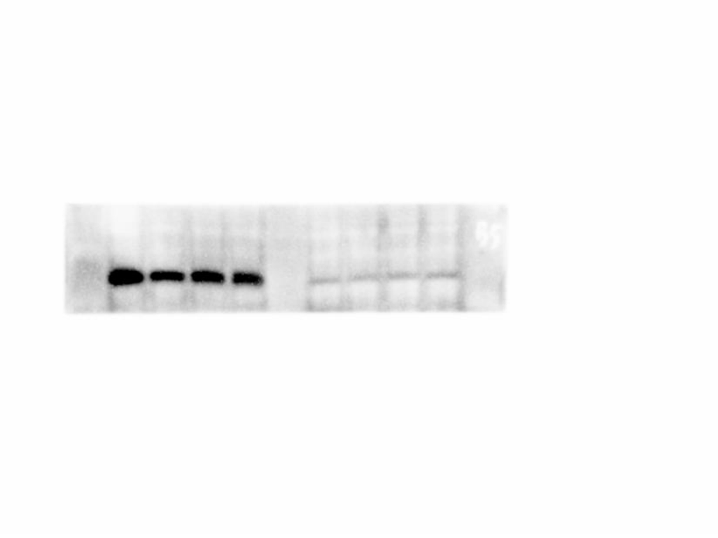

4E β-actin

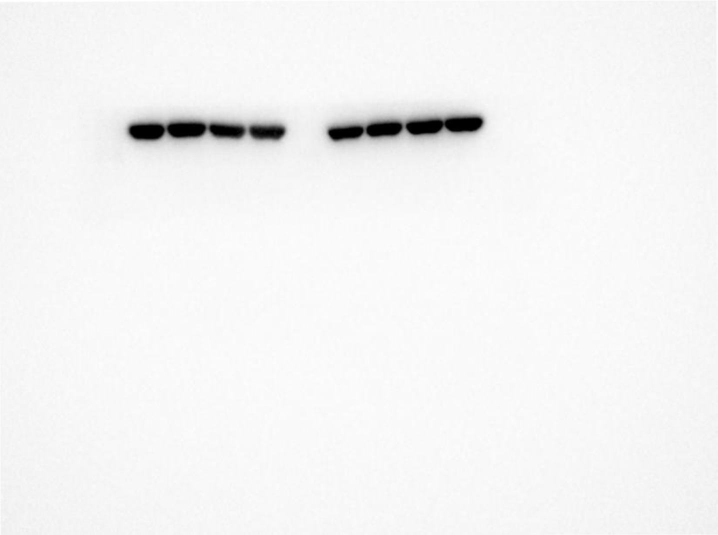

4F FXR1

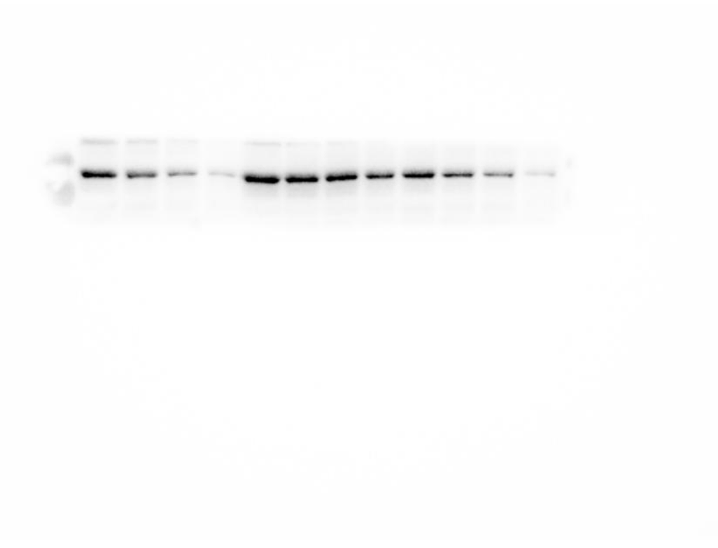

4F Flag

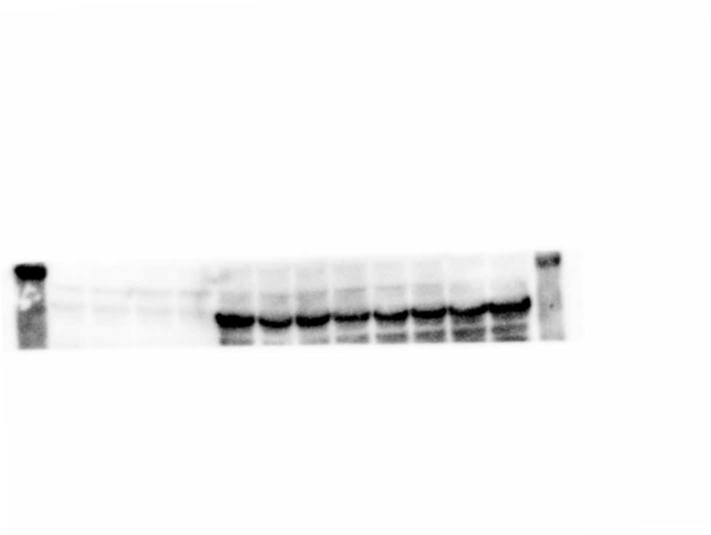

4F β-actin

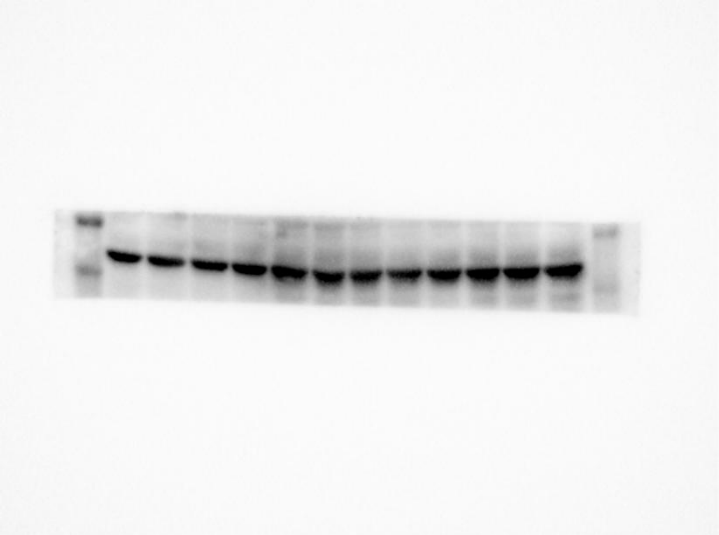

4G FXR1

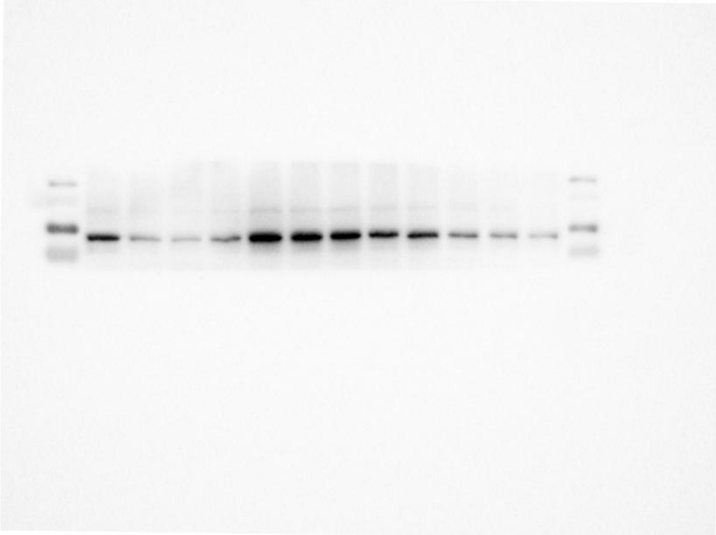

4G Flag

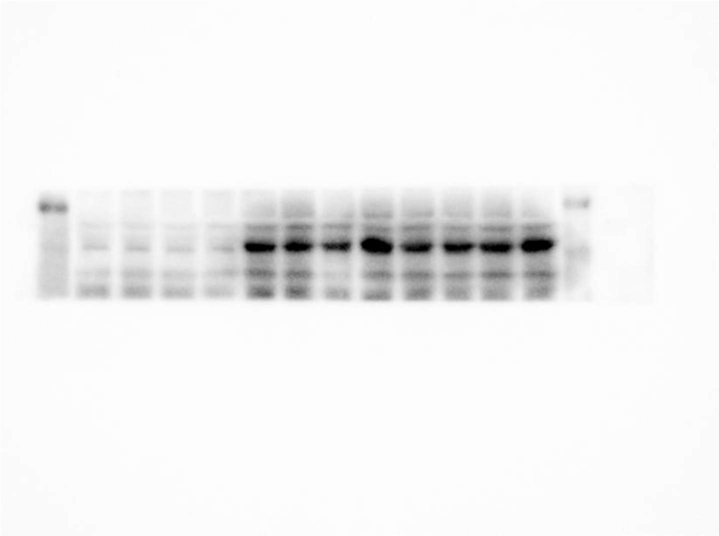

4G β-actin

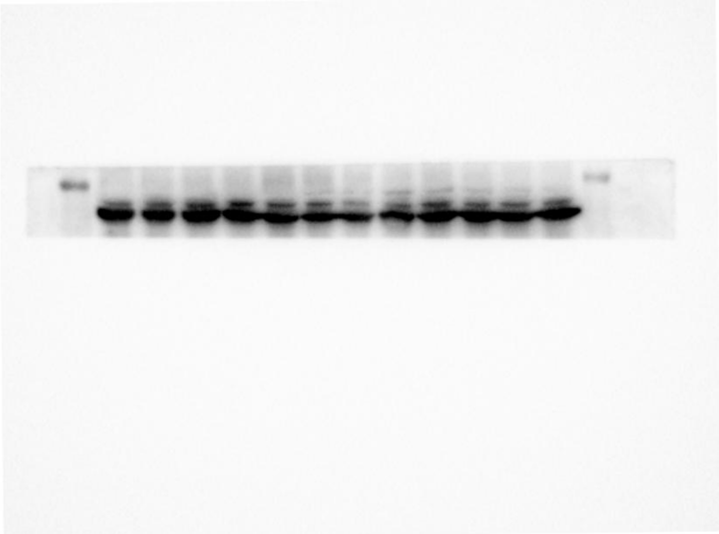

4H FXR1

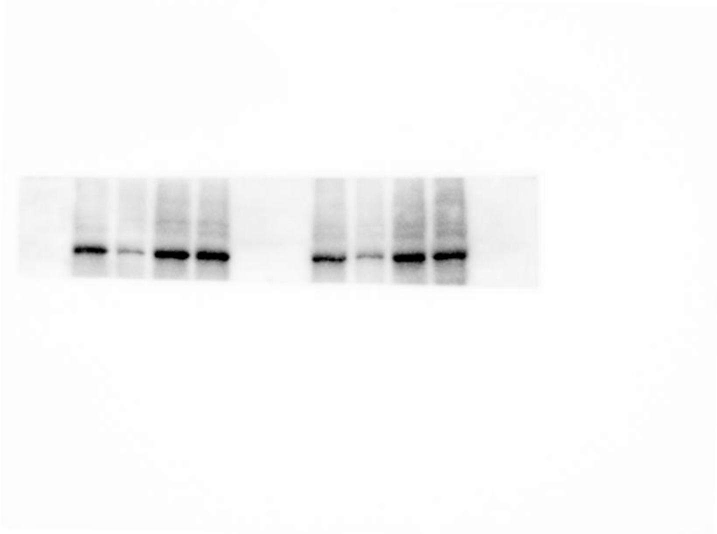

4H OTUD6B

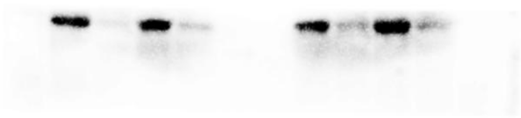

4H β-actin

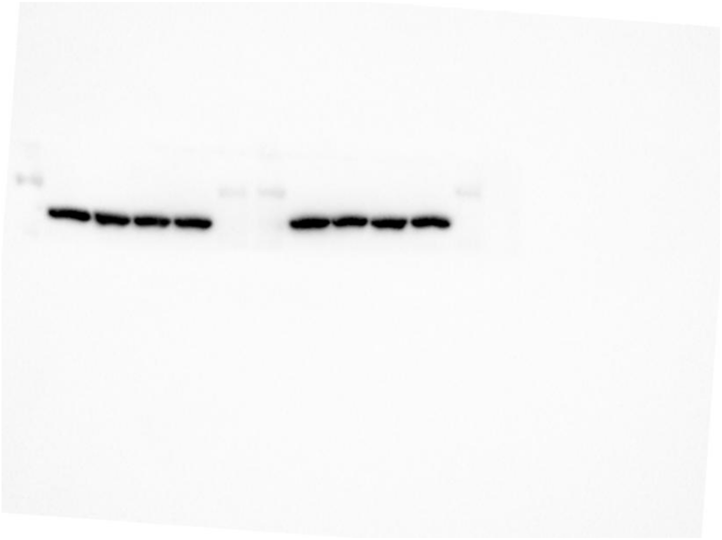

Figure 5

5A HA-ub

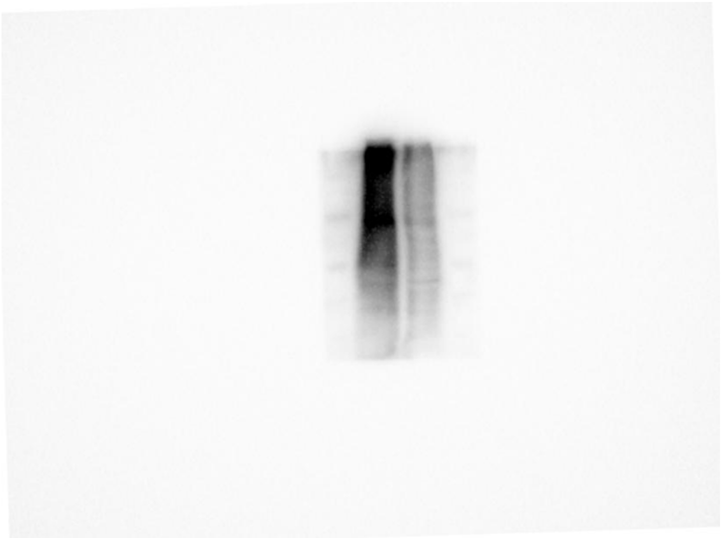

5A Myc

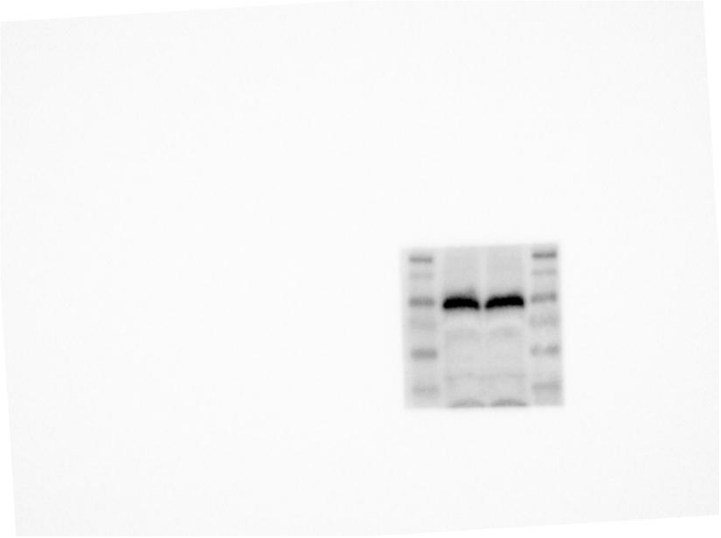

5A Flag

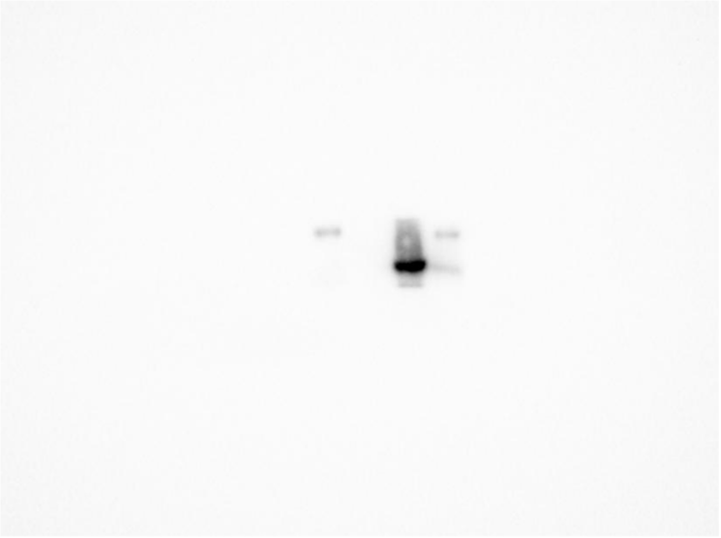

5A GAPDH

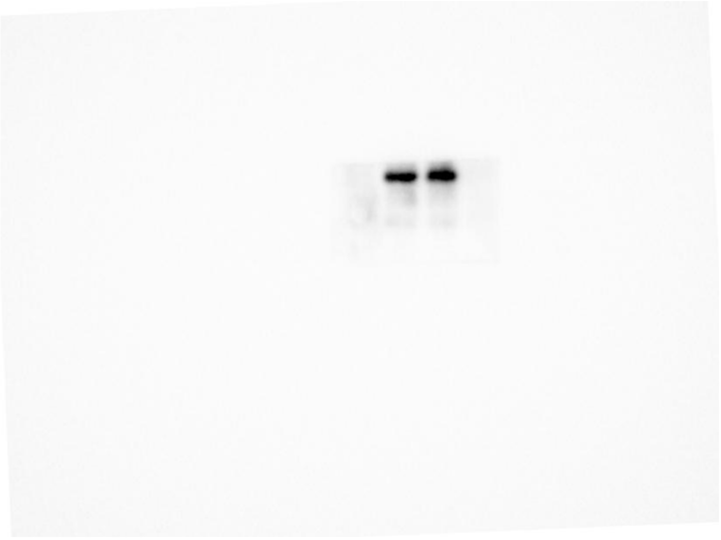

5B HA-ub

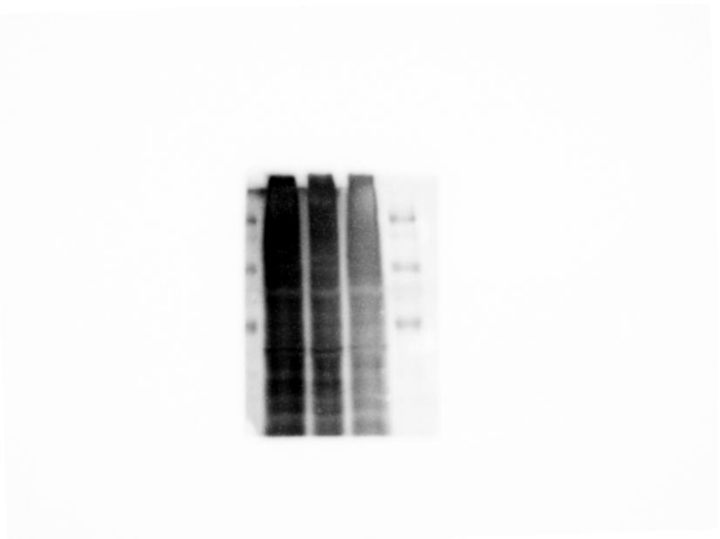

5B Myc

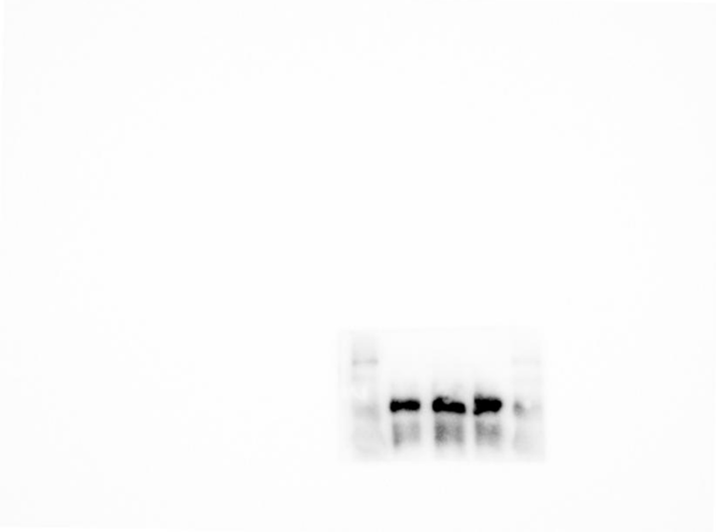

5B Flag

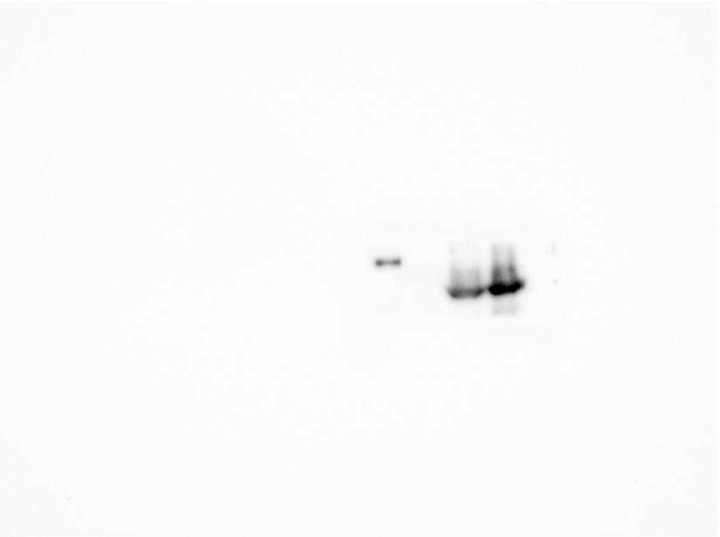

5B GAPDH

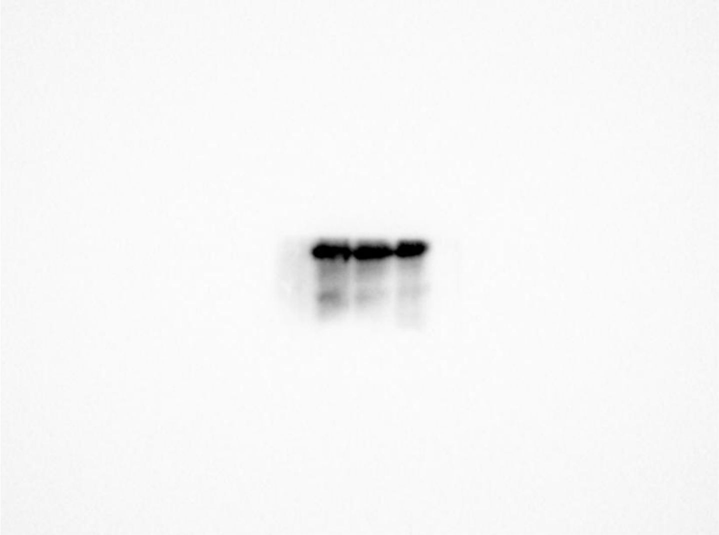

5C HA-ub

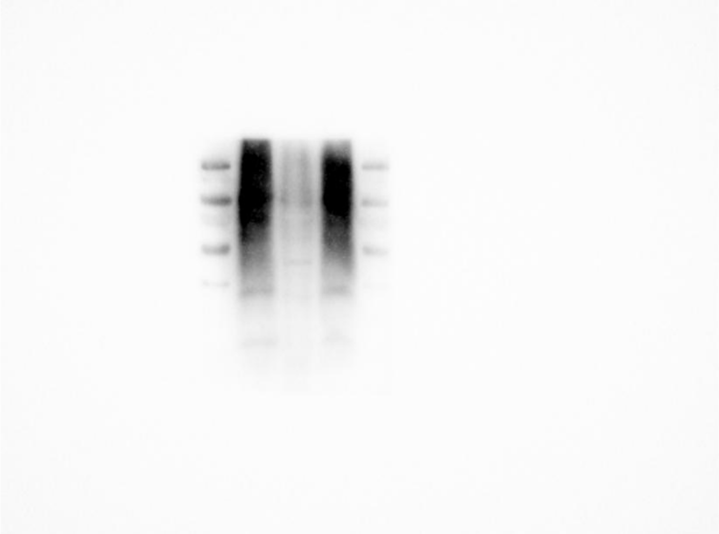

5C Myc

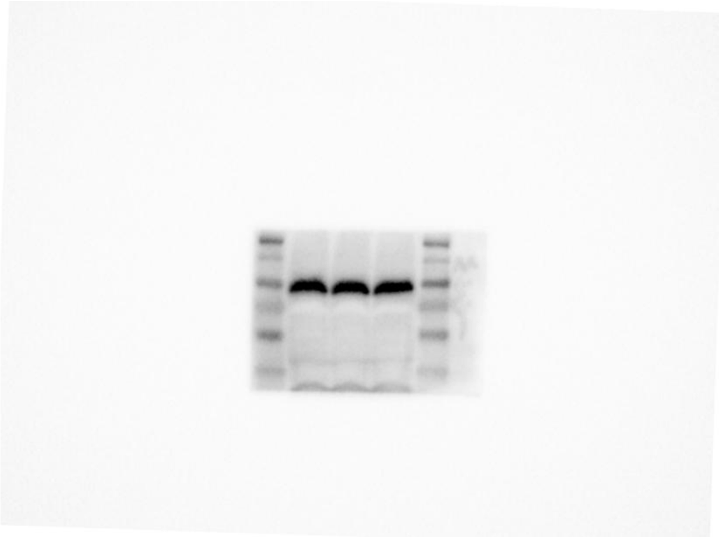

5C Flag

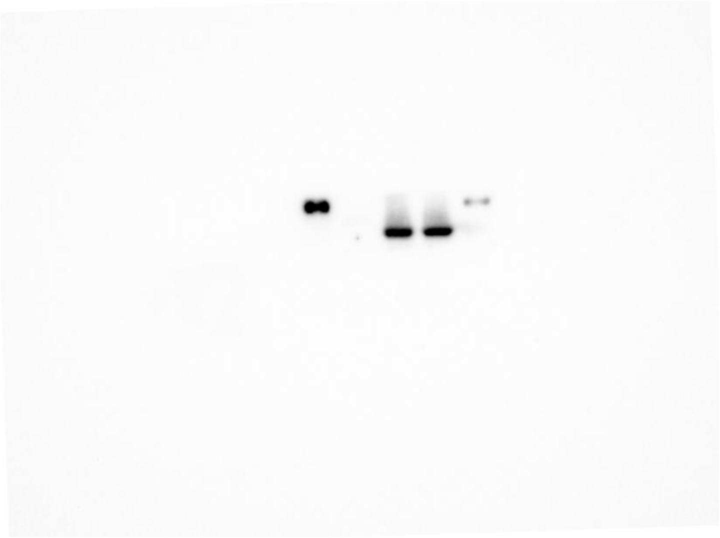

5C GAPDH

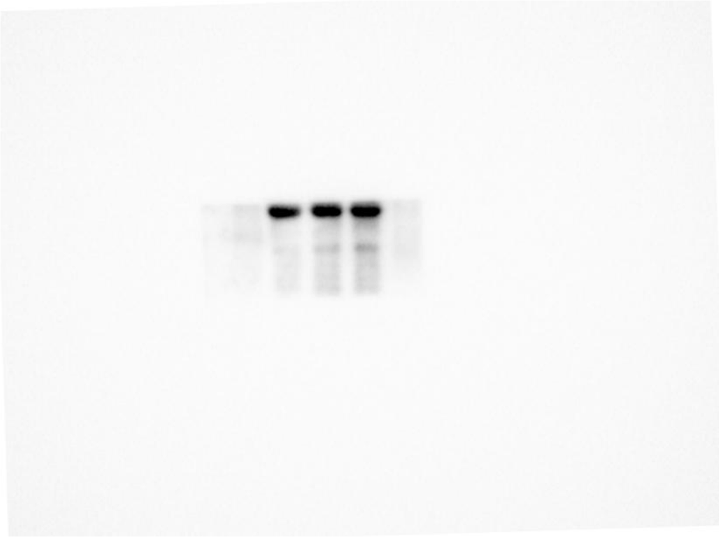

5D HA-ub

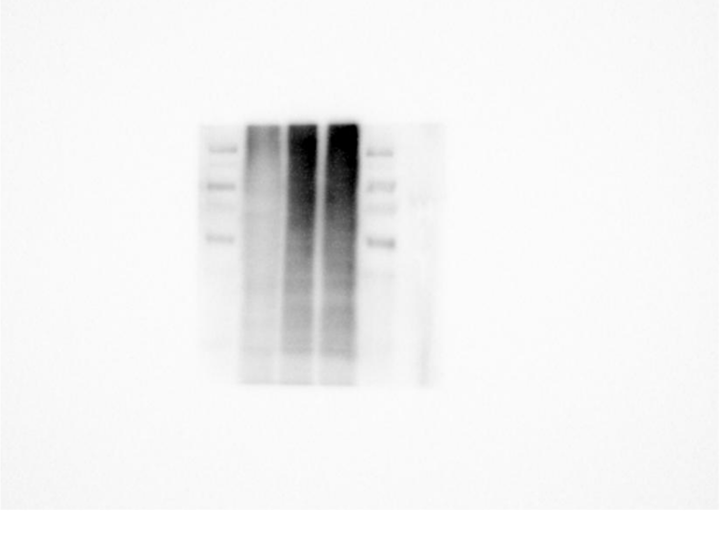

5D Myc

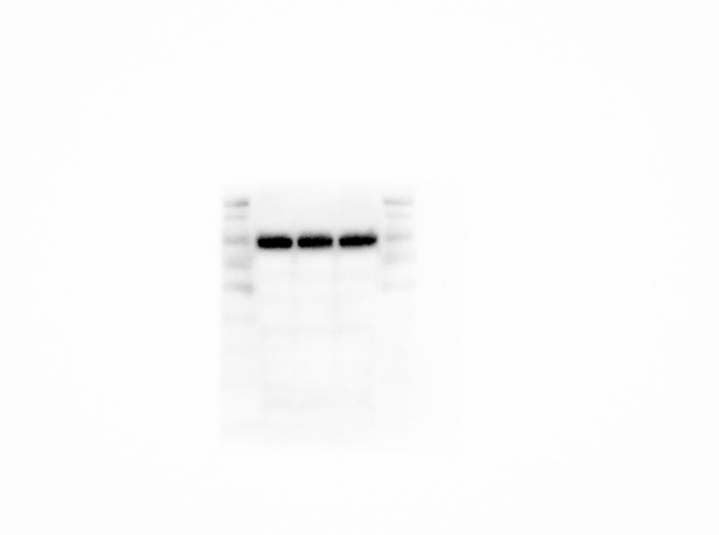

5D OTUD6B

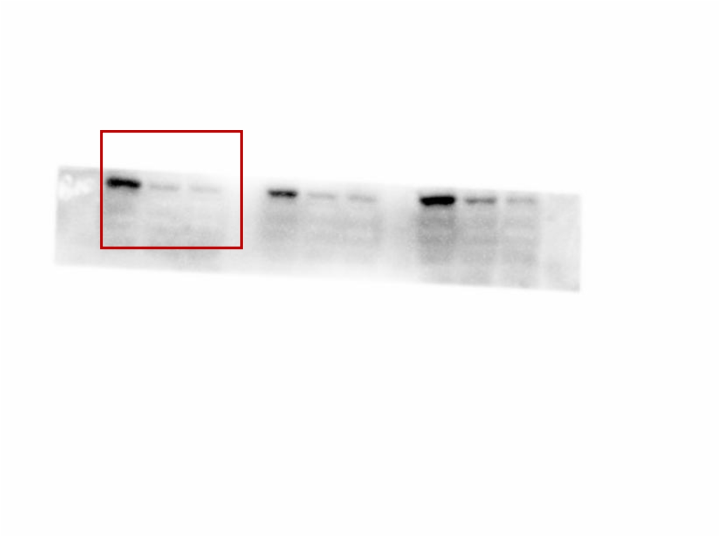

5D β-actin

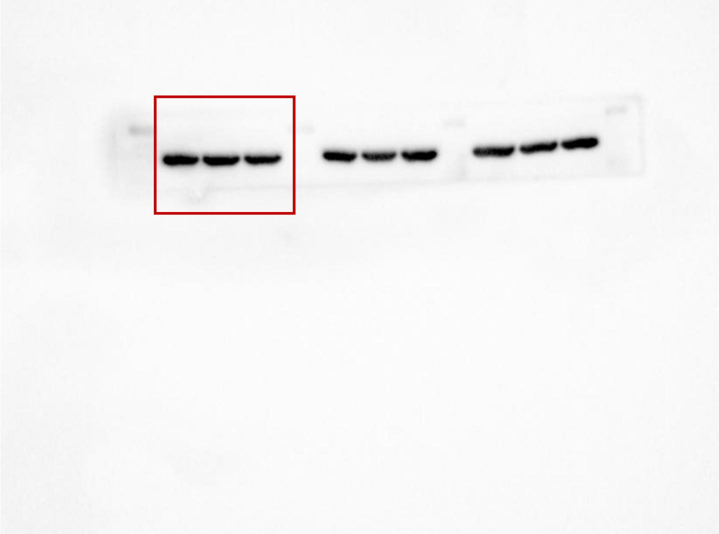

5E HA-ub

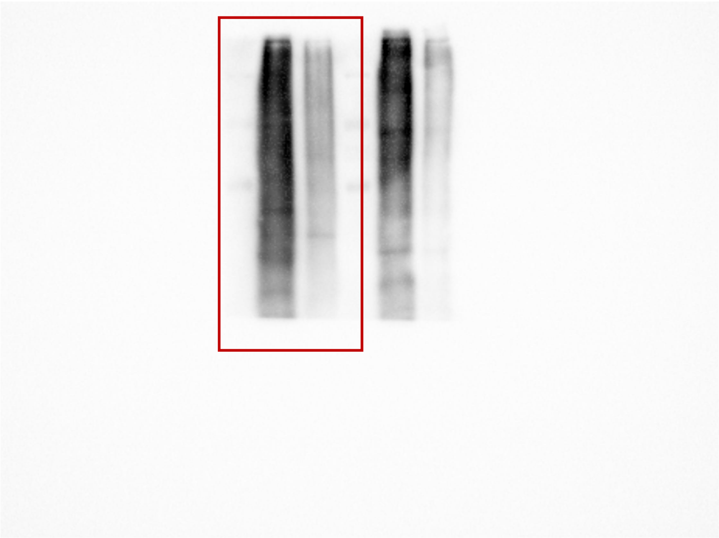

5E FXR1

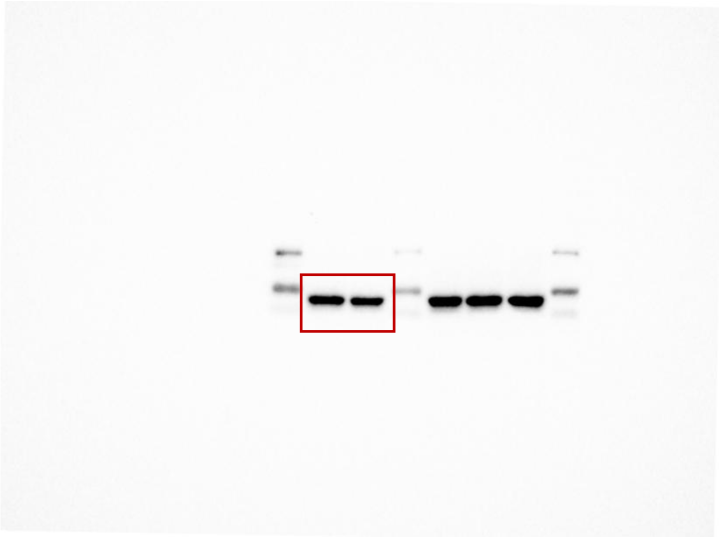

5E Flag

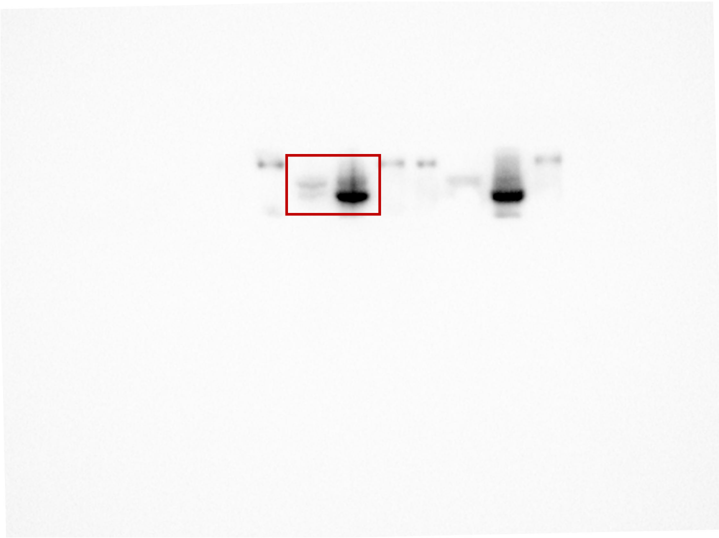

5E GAPDH

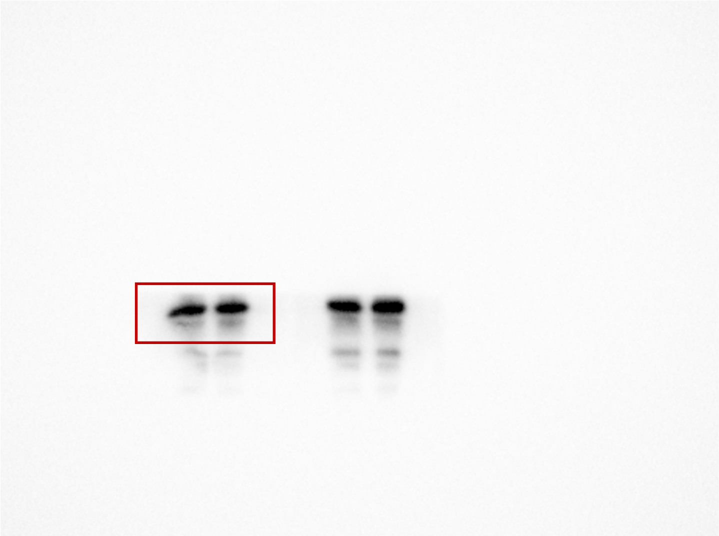

5F HA-ub

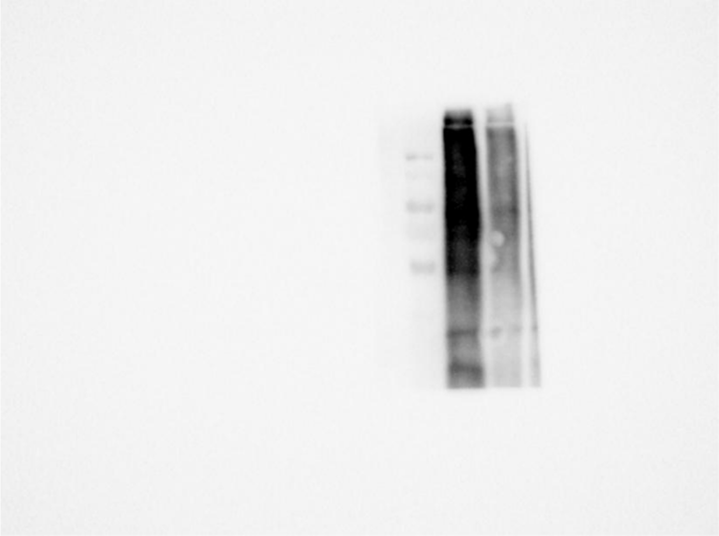

5F FXR1

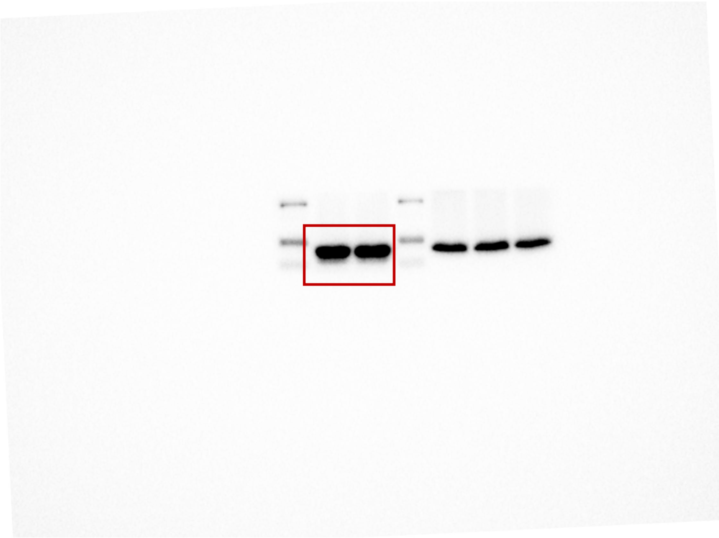

5F Flag

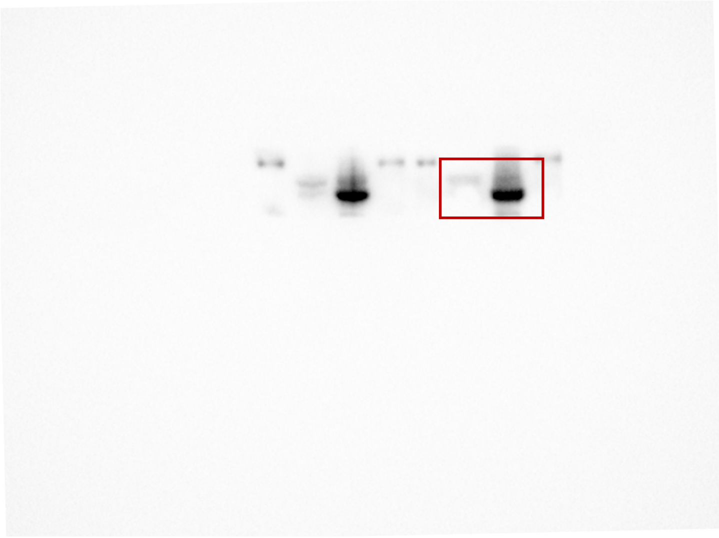

5F GAPDH

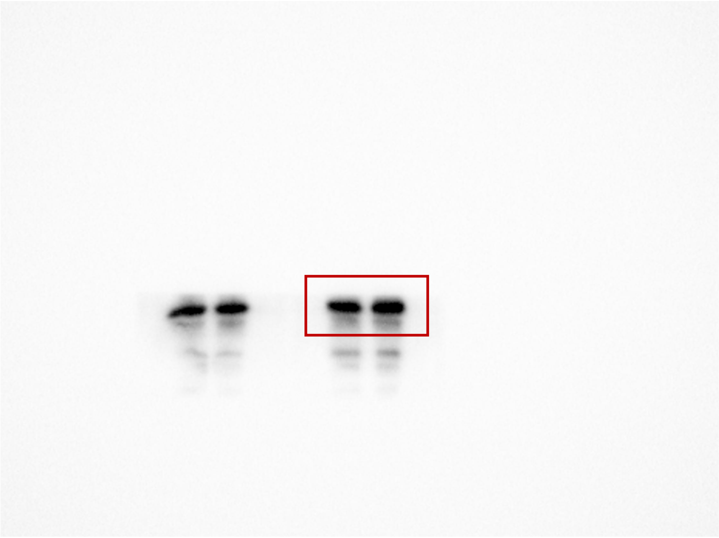

5G HA-ub

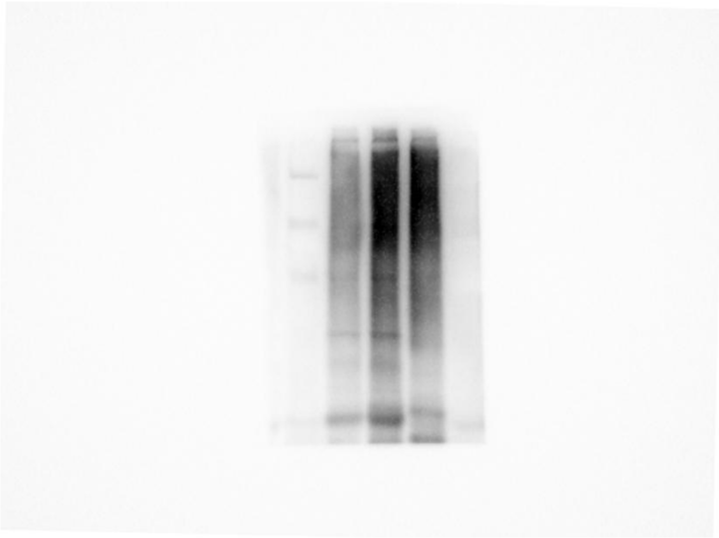

5G FXR1

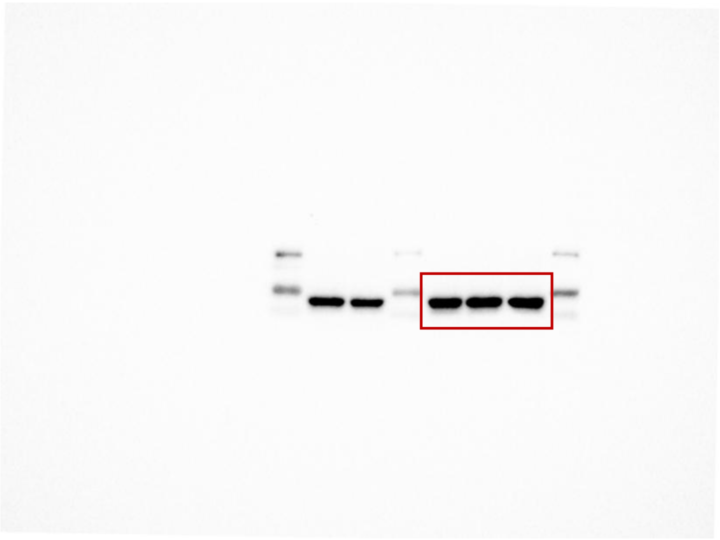

5G OTUD6B

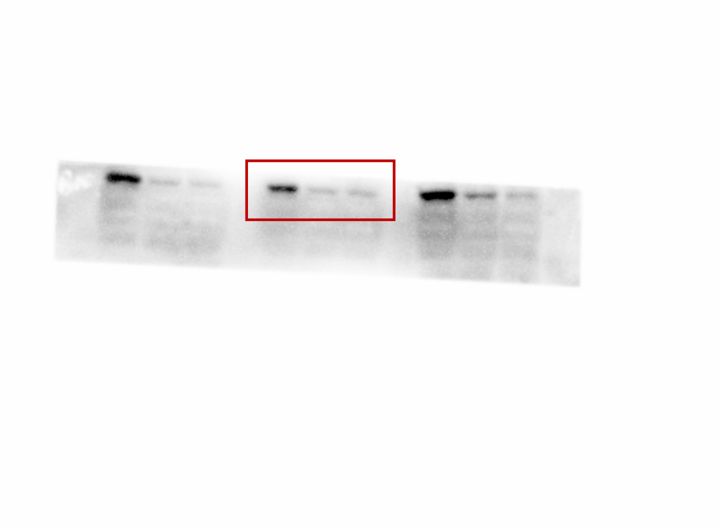

5G β-actin

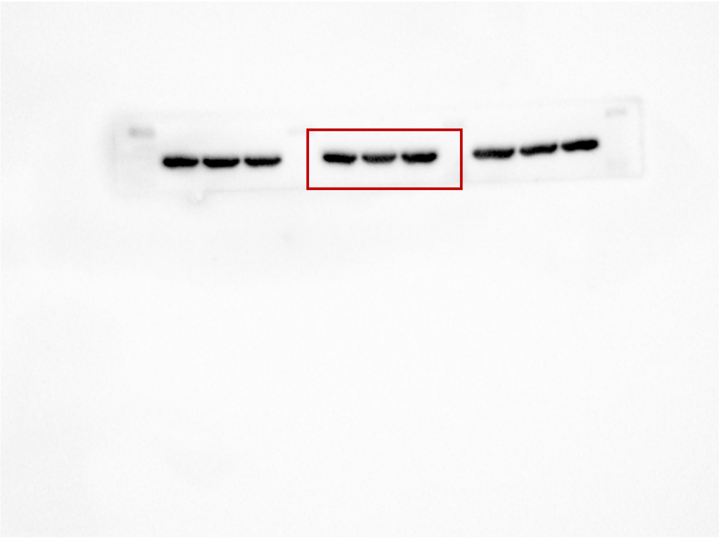

5H HA-ub

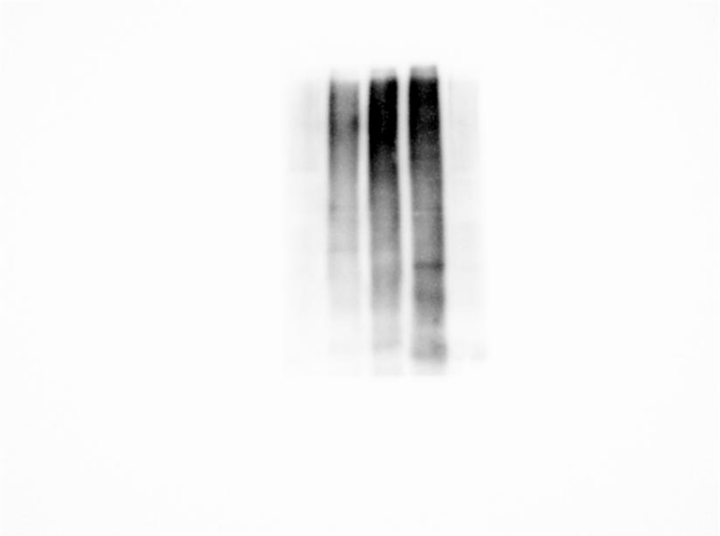

5H FXR1

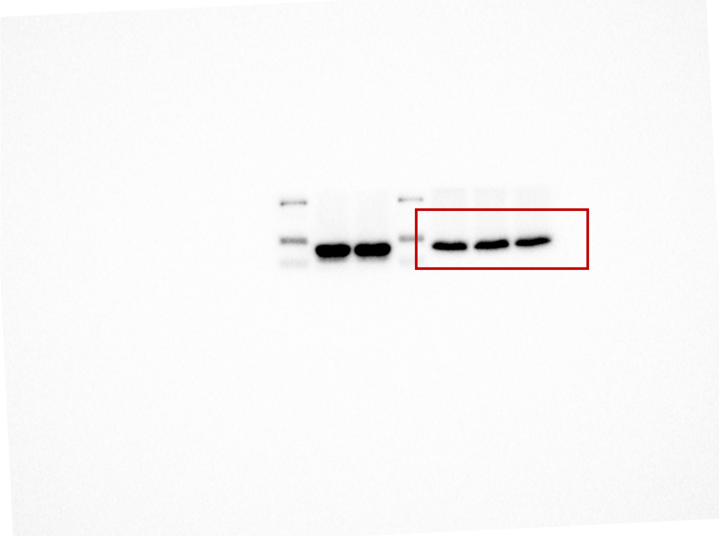

5H OTUD6B

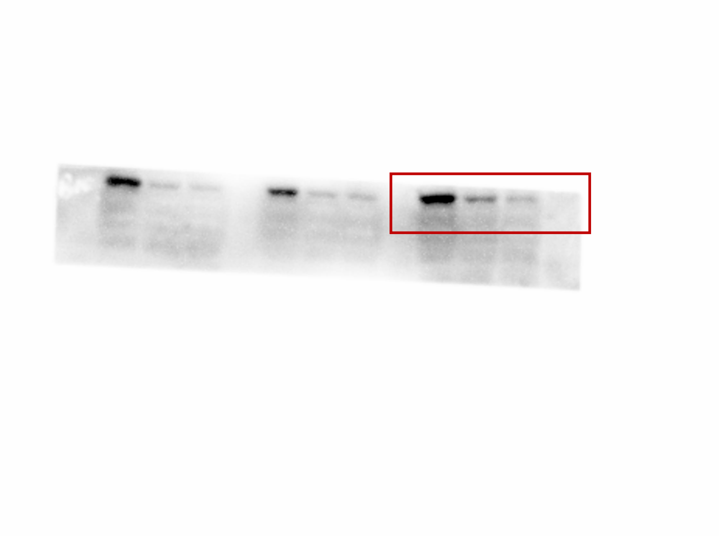

5H β-actin

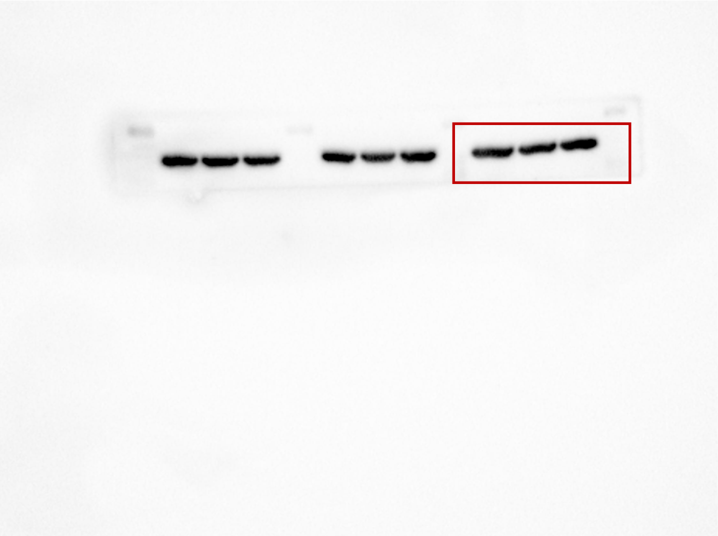

5I HA-ub

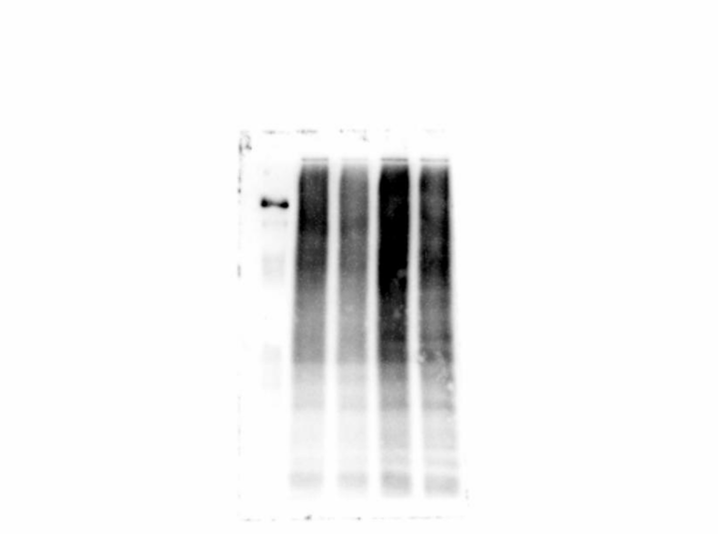

5I Myc

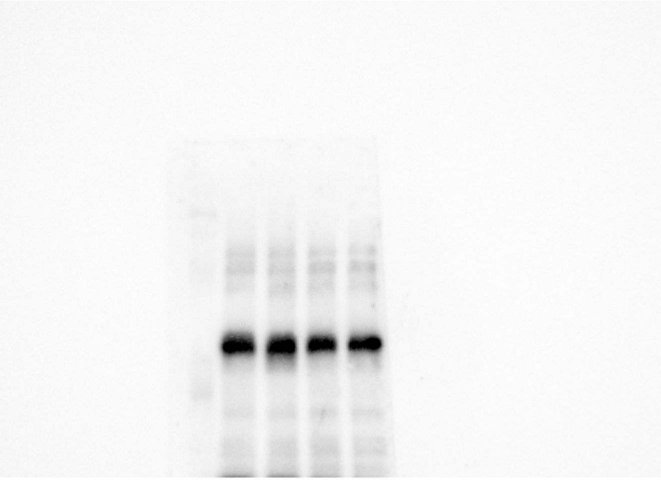

5I Flag

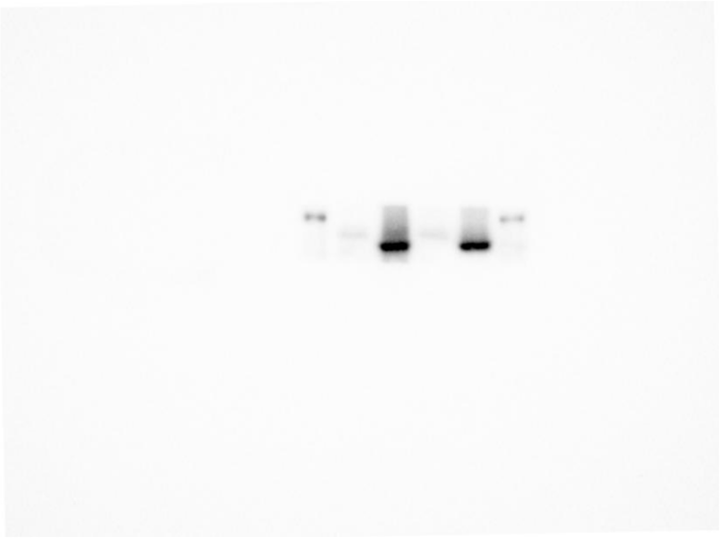

5I GAPDH

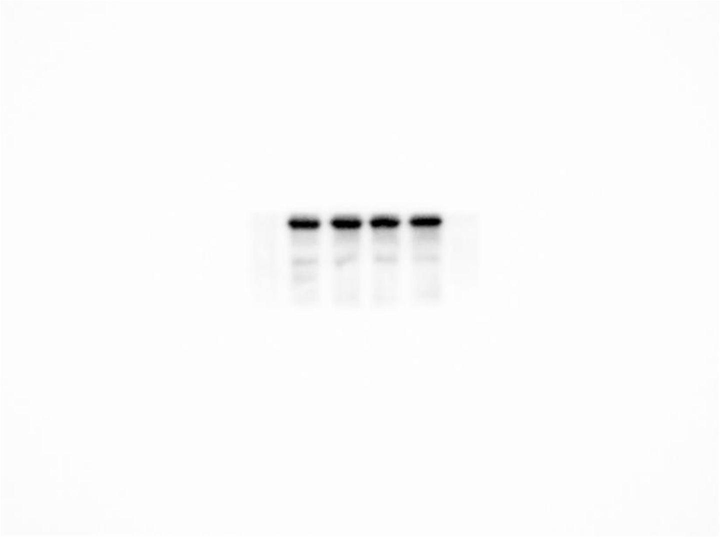

5J HA-ub

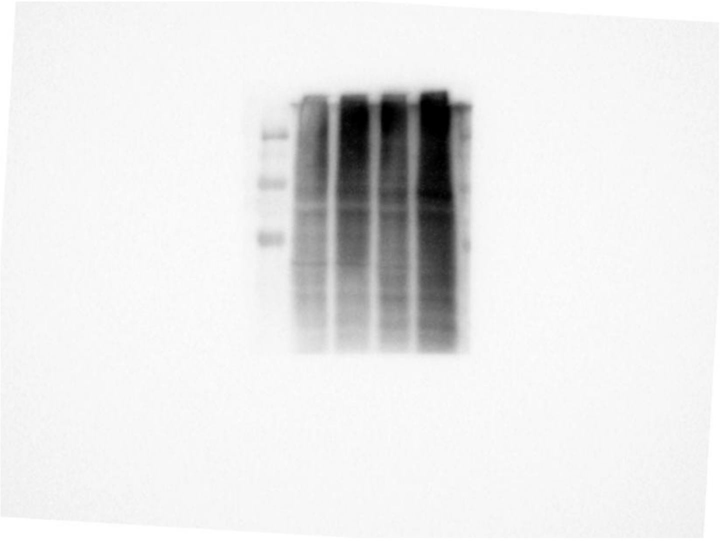

5J Myc

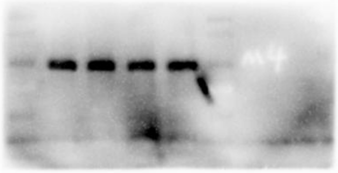

5J OTUD6B

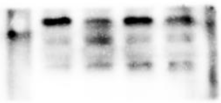

5J β-actin

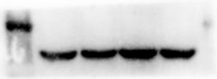

5K HA-ub

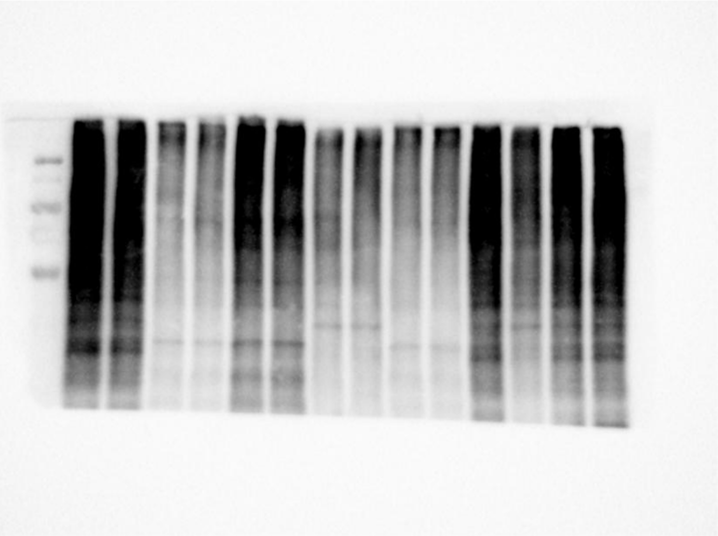

5K Myc

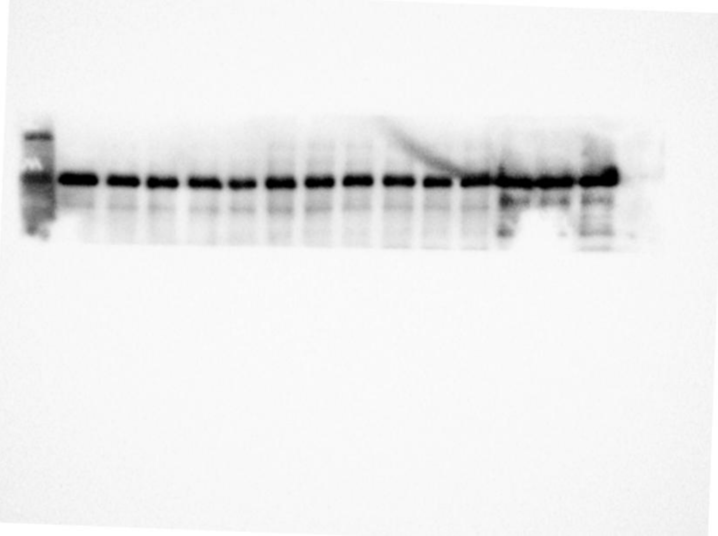

5K Flag

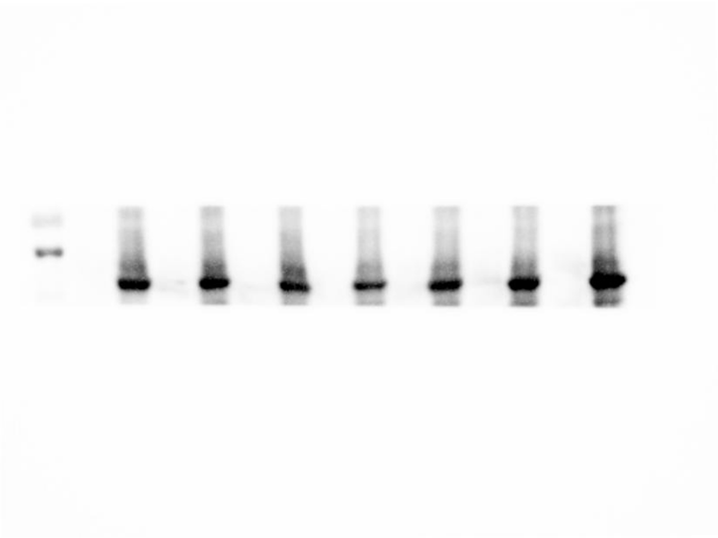

5K GAPDH

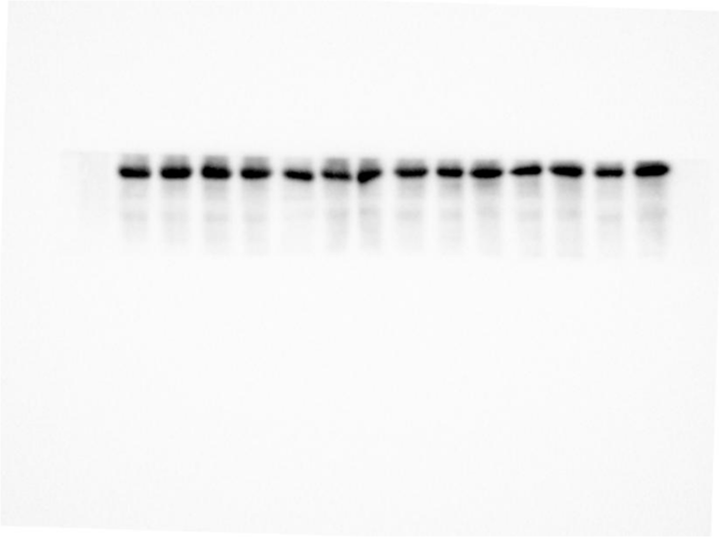

Figure 6

6F FXR1 HCT116

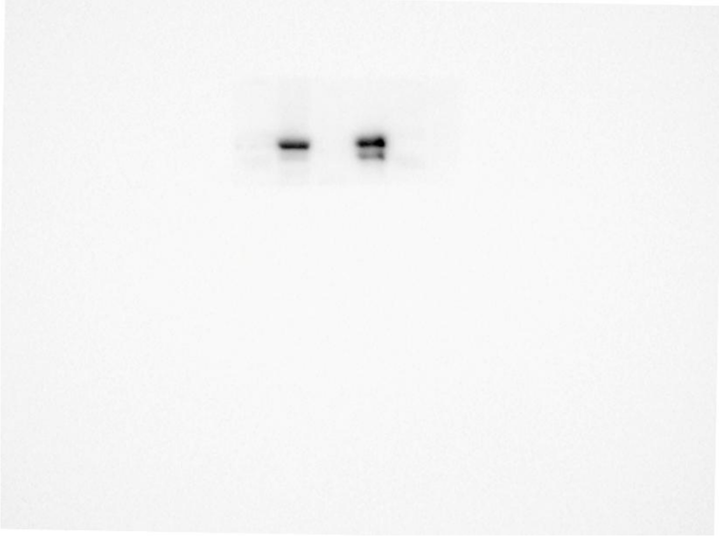

6F FXR1 SW480

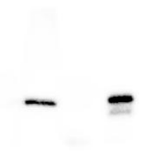

6I FXR1

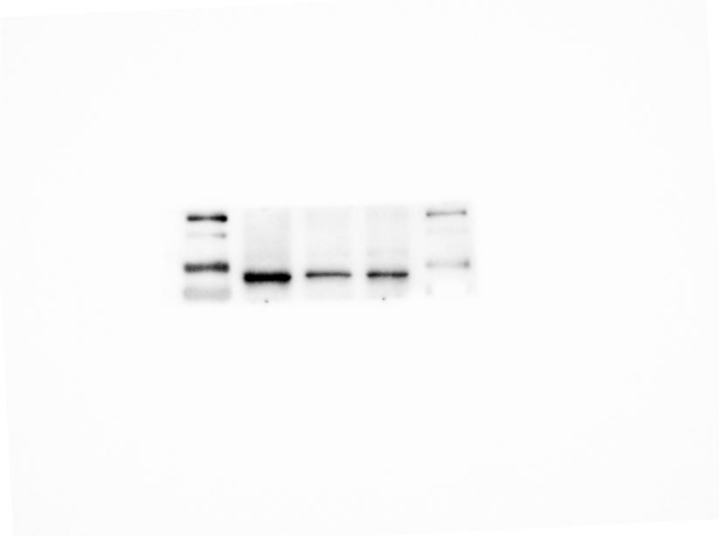

6I MEK2

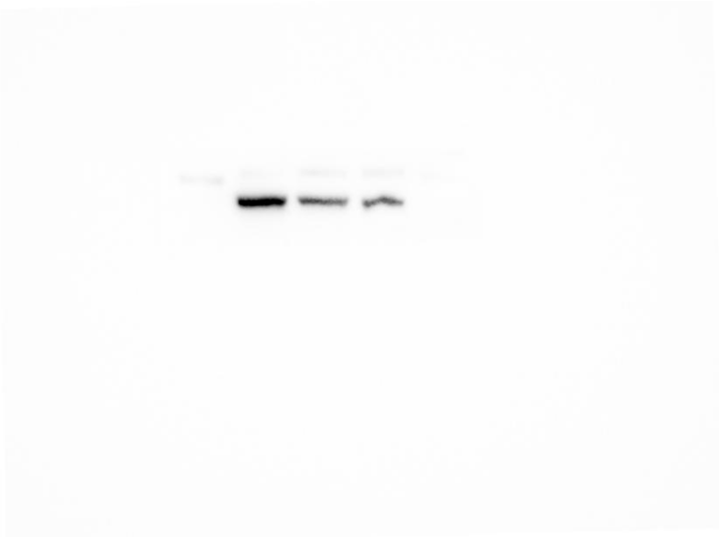

6I p-ERK1/2

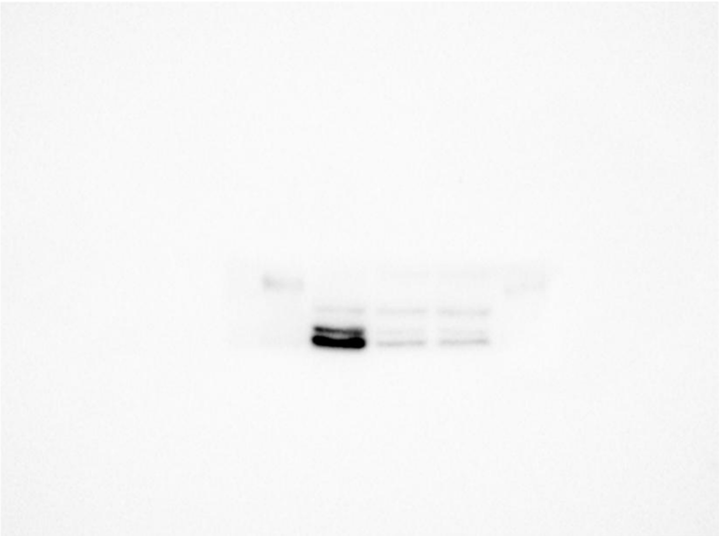

6I ERK1/2

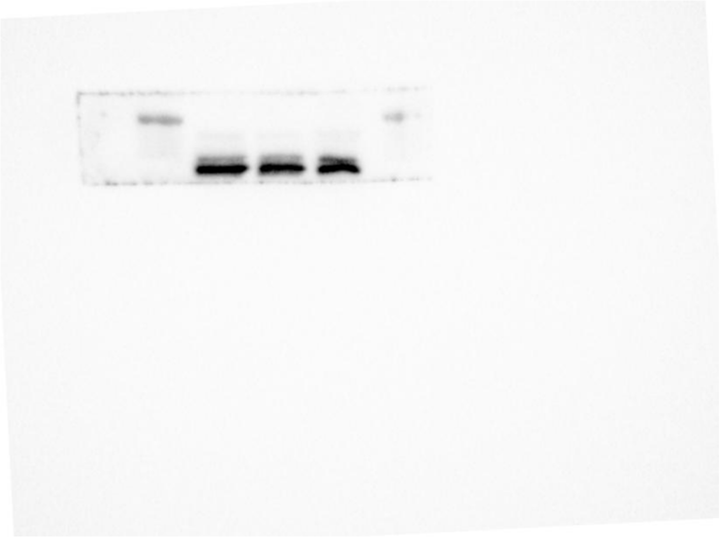

6I GAPDH

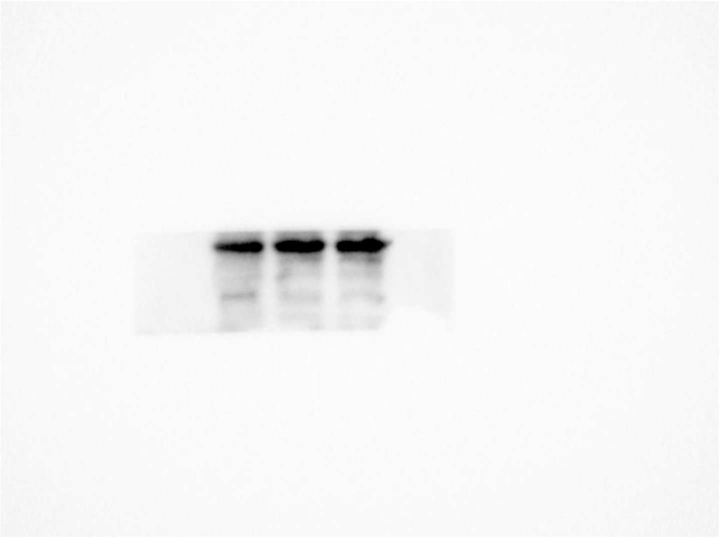

6J FXR1

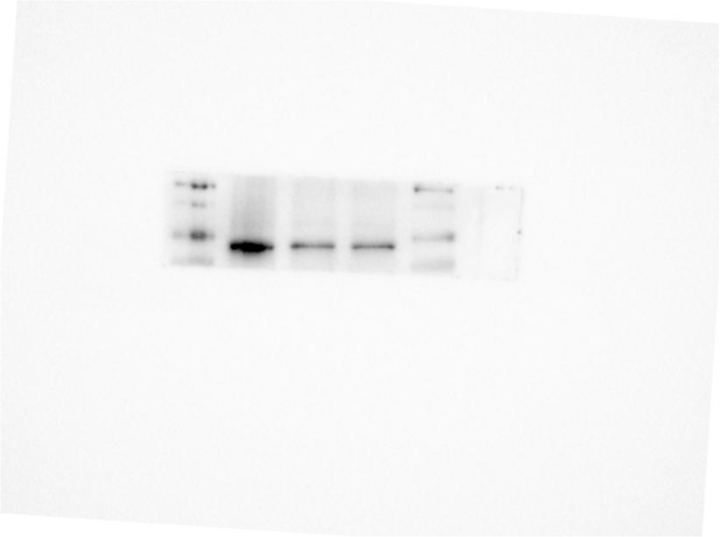

6J MEK2

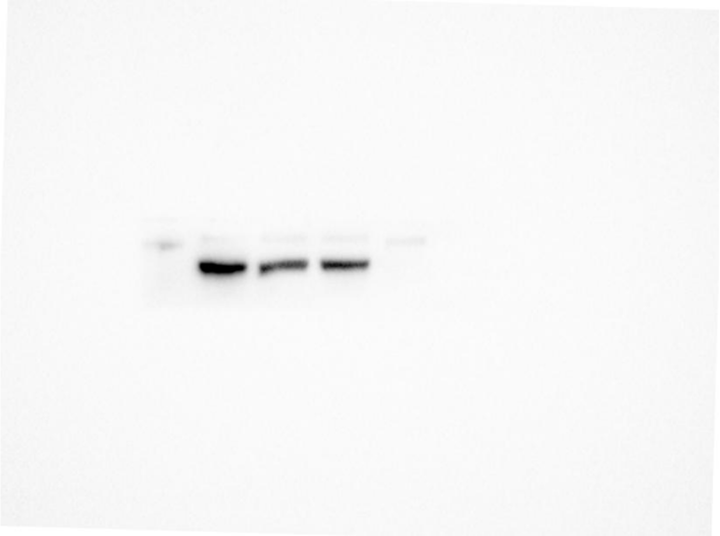

6J p-ERK1/2

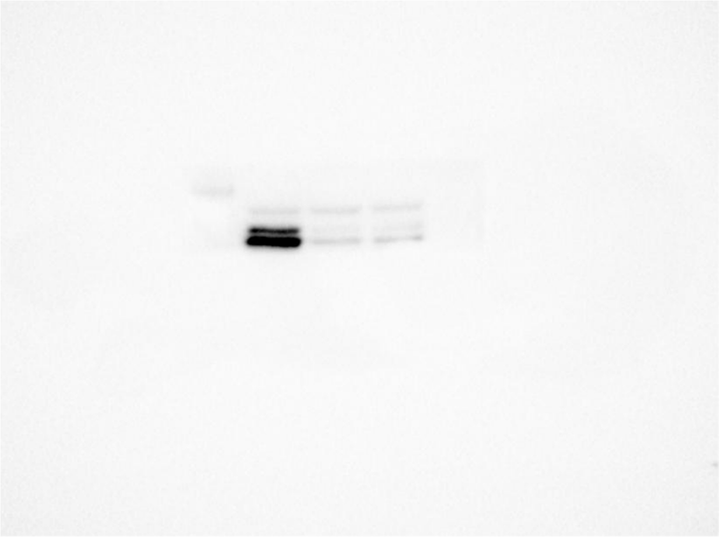

6J ERK1/2

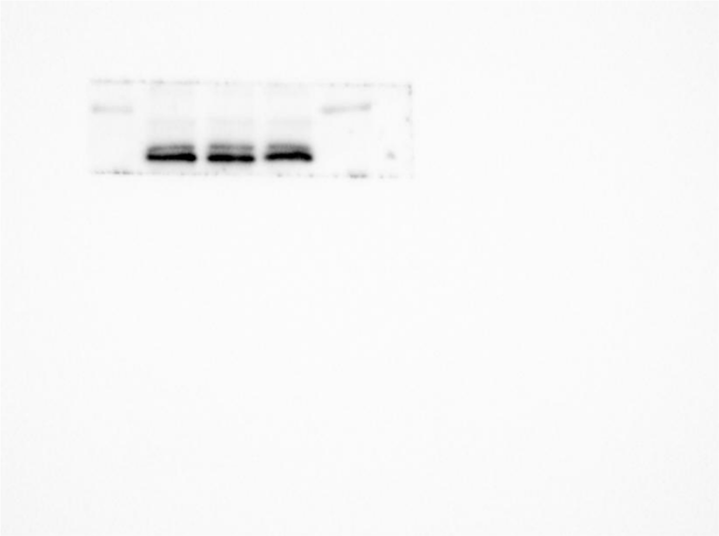

6J GAPDH

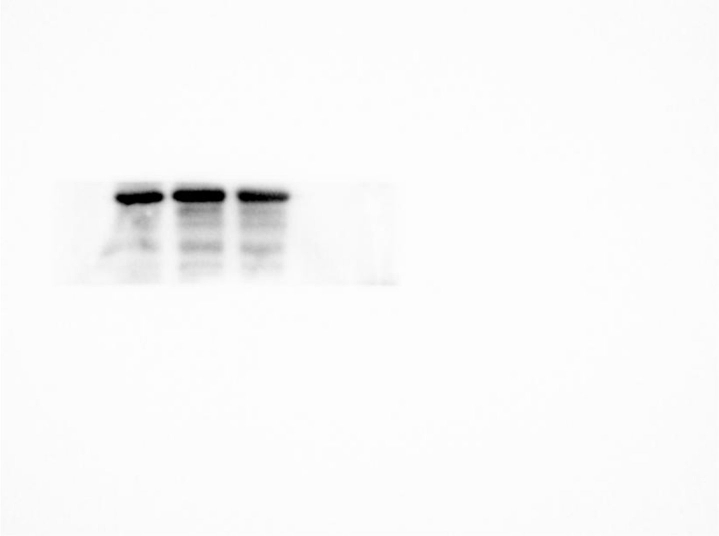

6K FXR1

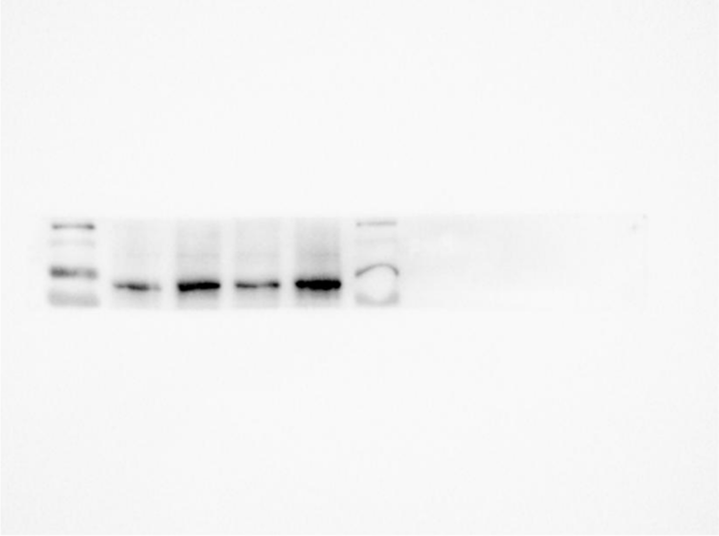

6K MEK2

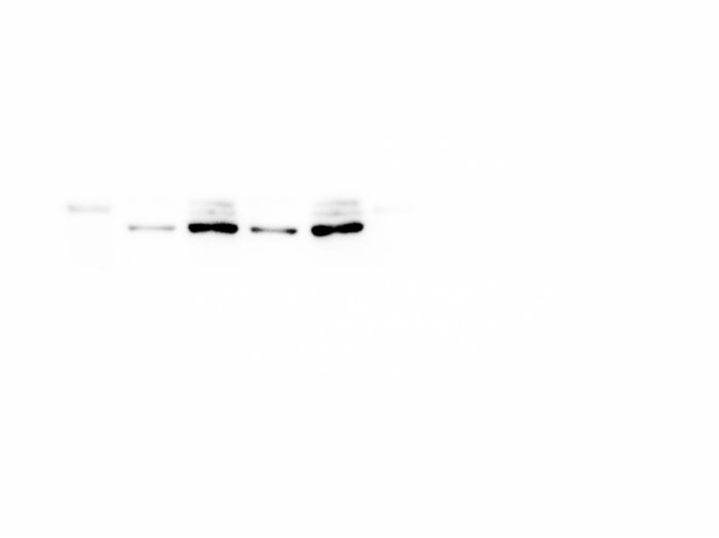

6K p-ERK1/2

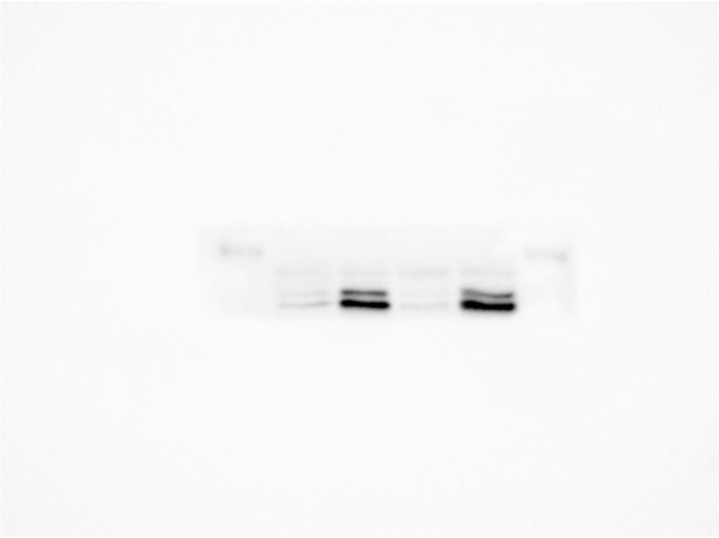

6K ERK1/2

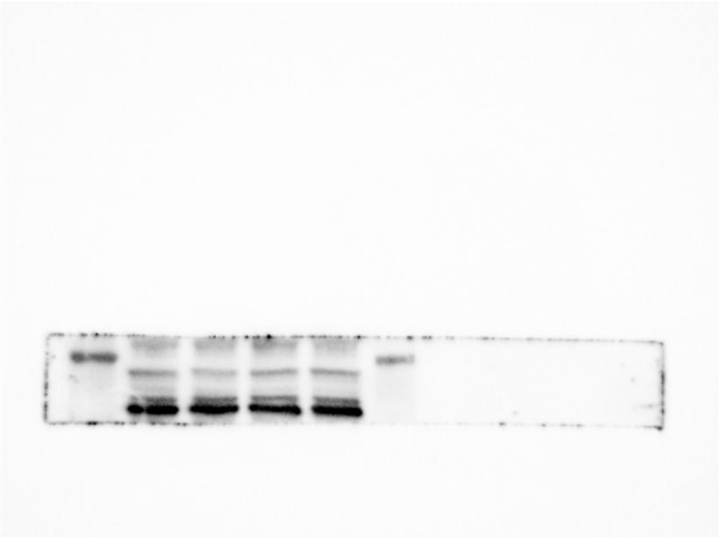

6K GAPDH

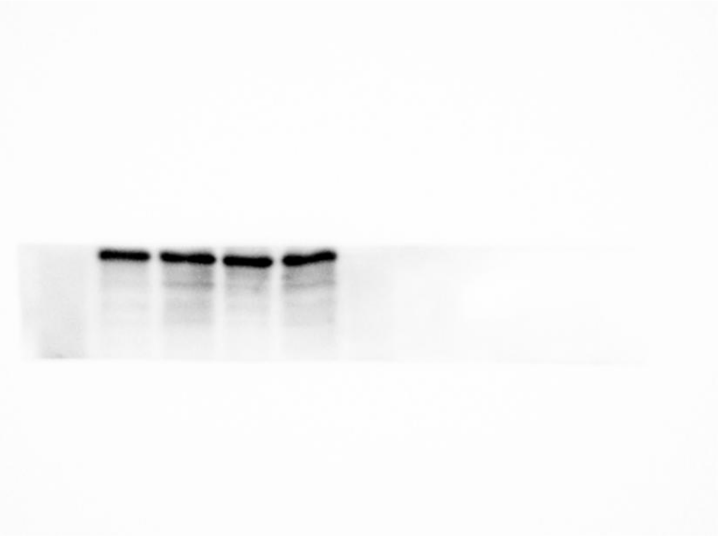

6L FXR1

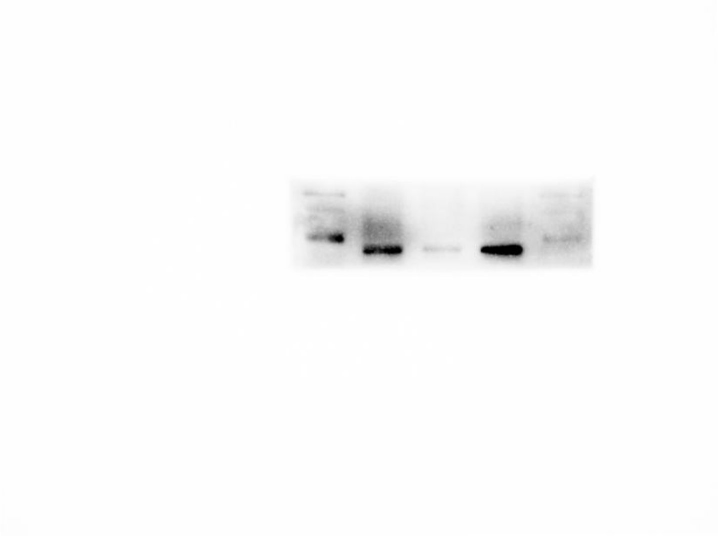

6L MEK2

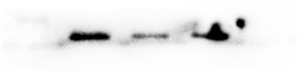

6L p-ERK1/2

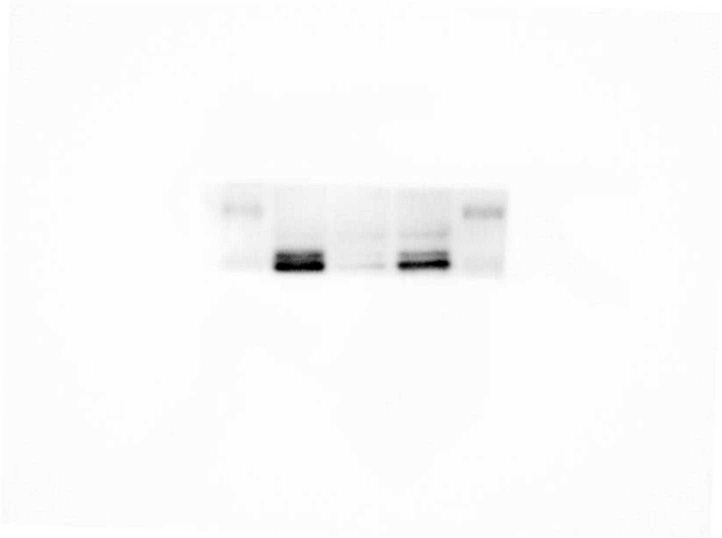

6L ERK1/2

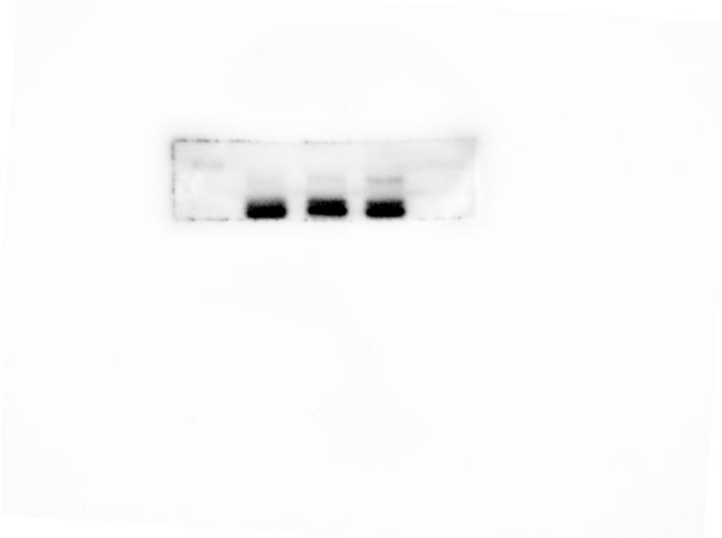

6L OTUD6B

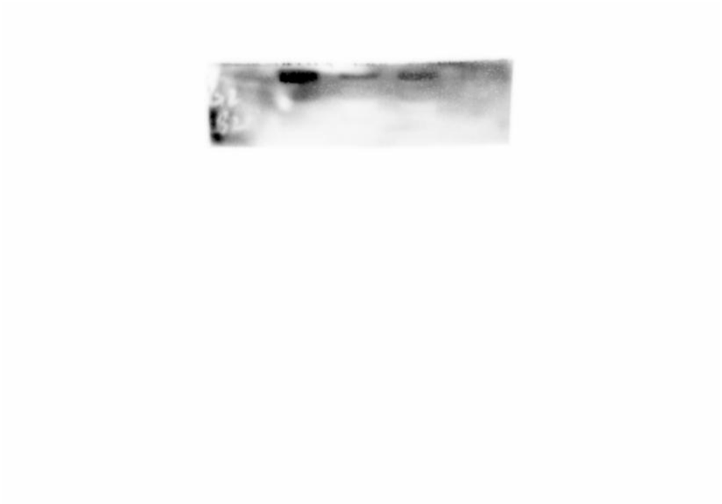

6L GAPDH

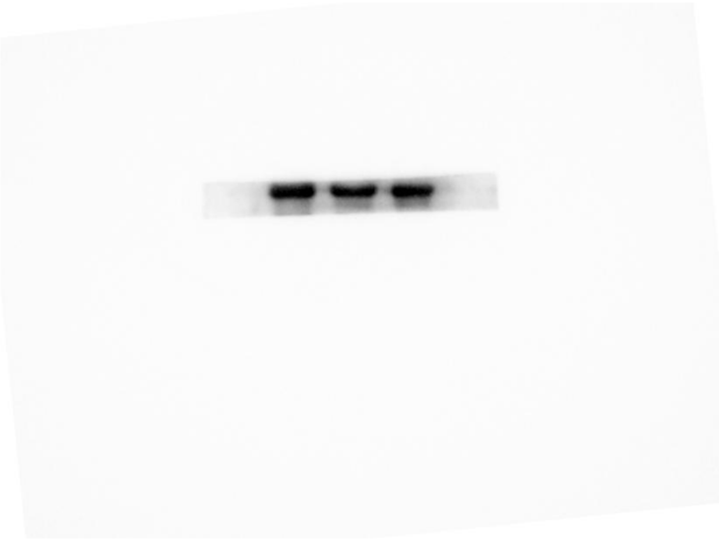

6M FXR1

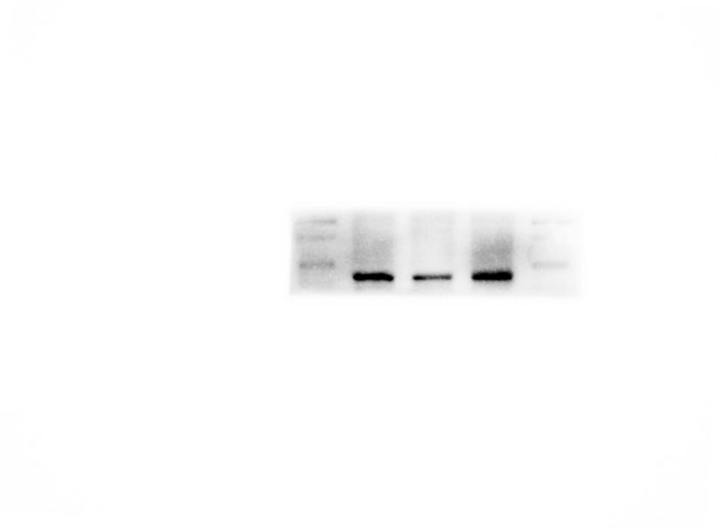

6M MEK2

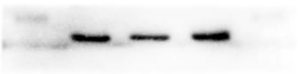

6M p-ERK1/2

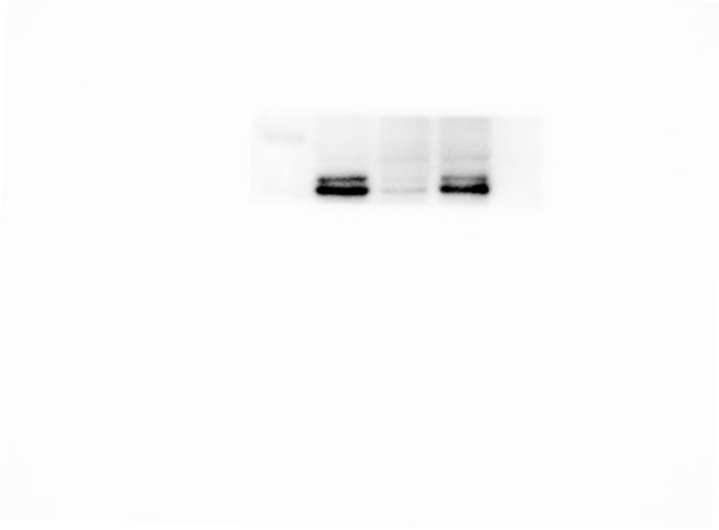

6M ERK1/2

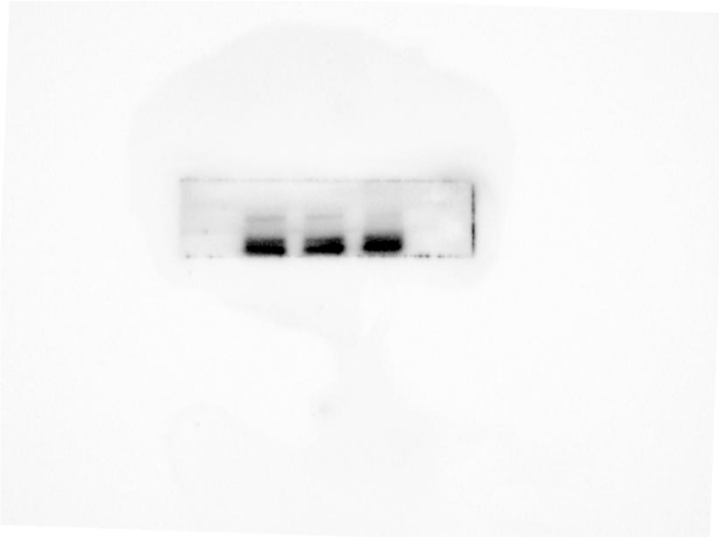

6M OTUD6B

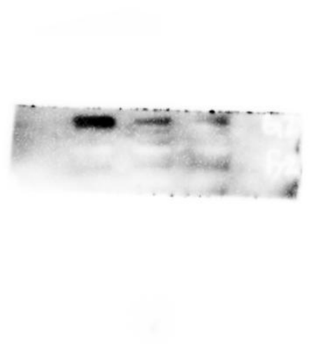

6M GAPDH

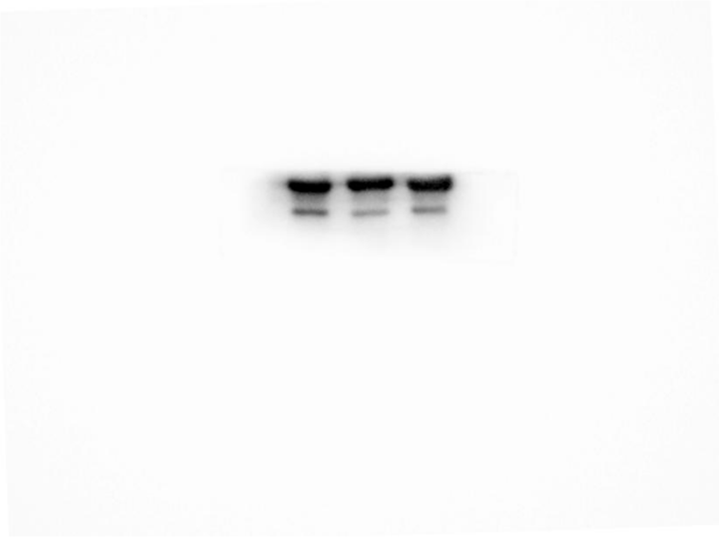

6N FXR1

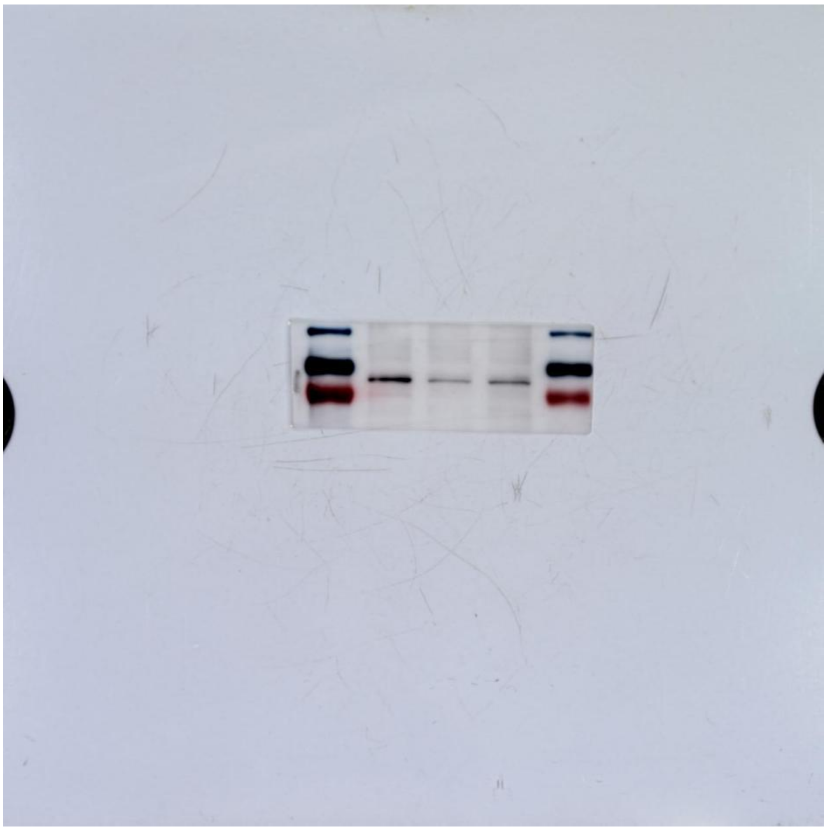

6N OTUD6B

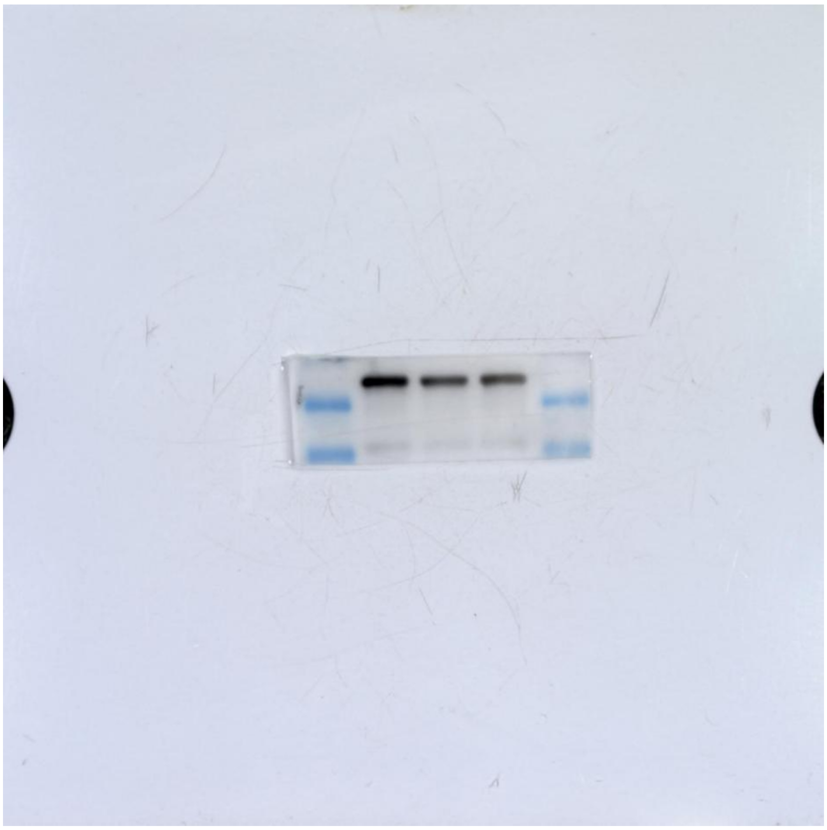

6N β-actin

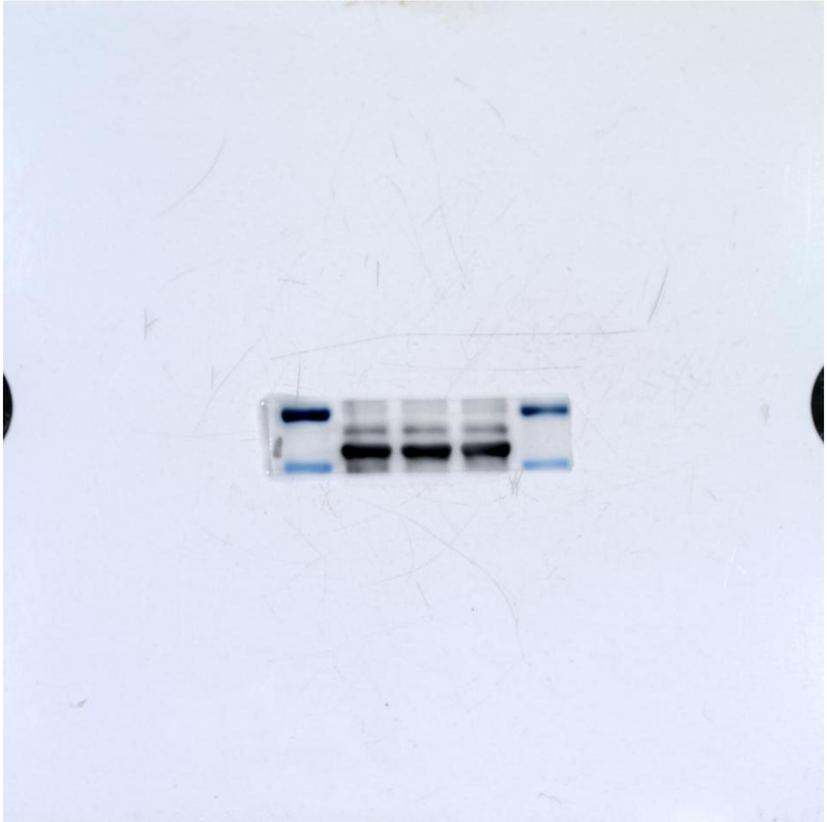

6O FXR1

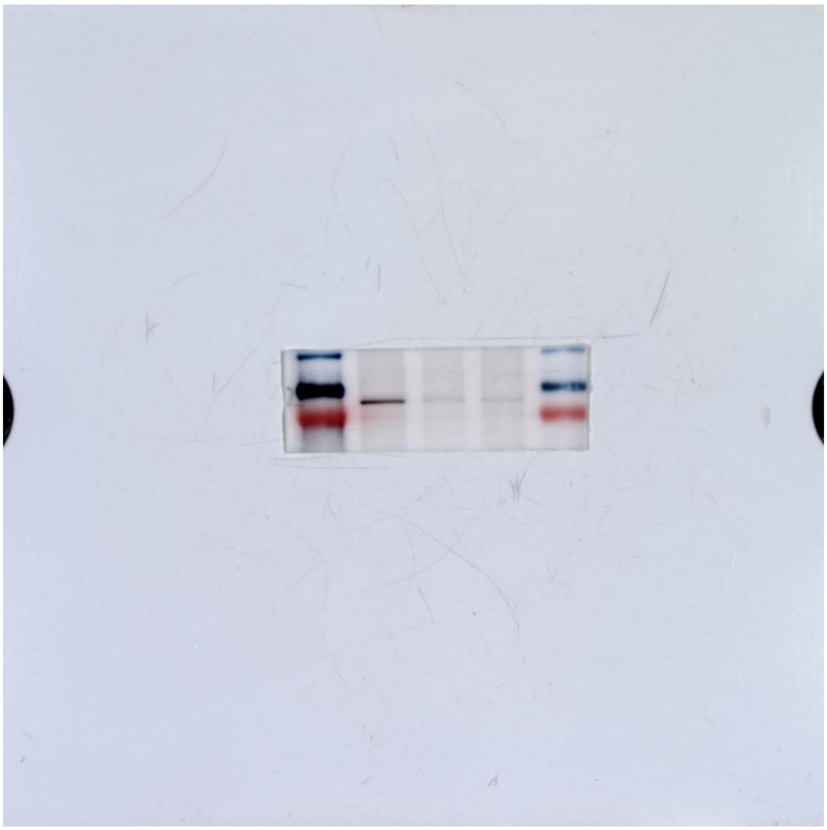

6O OTUD6B

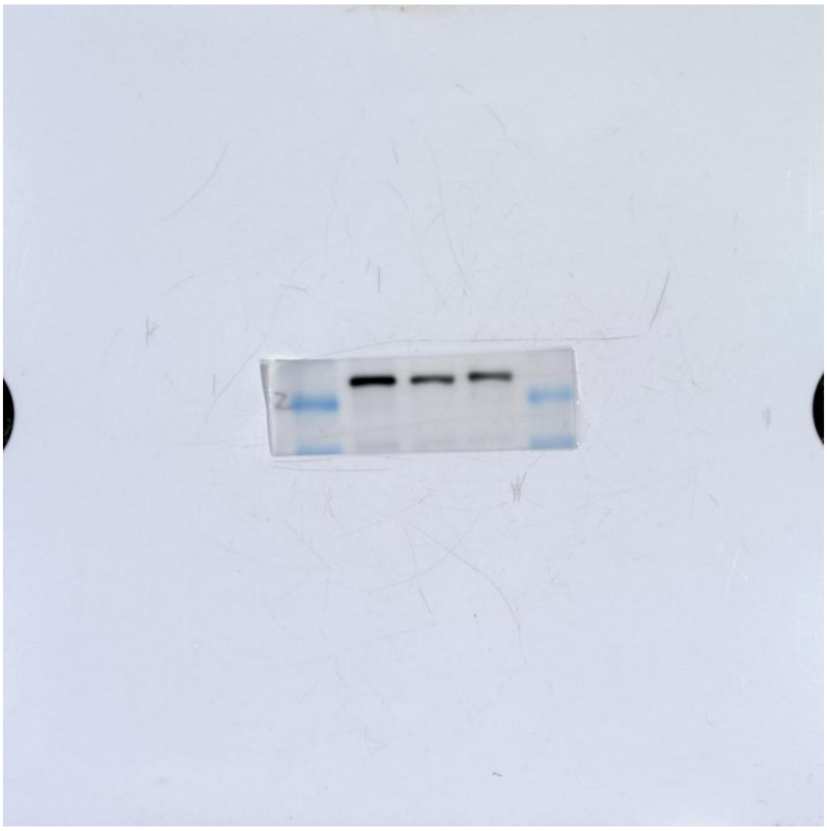

6O β-actin

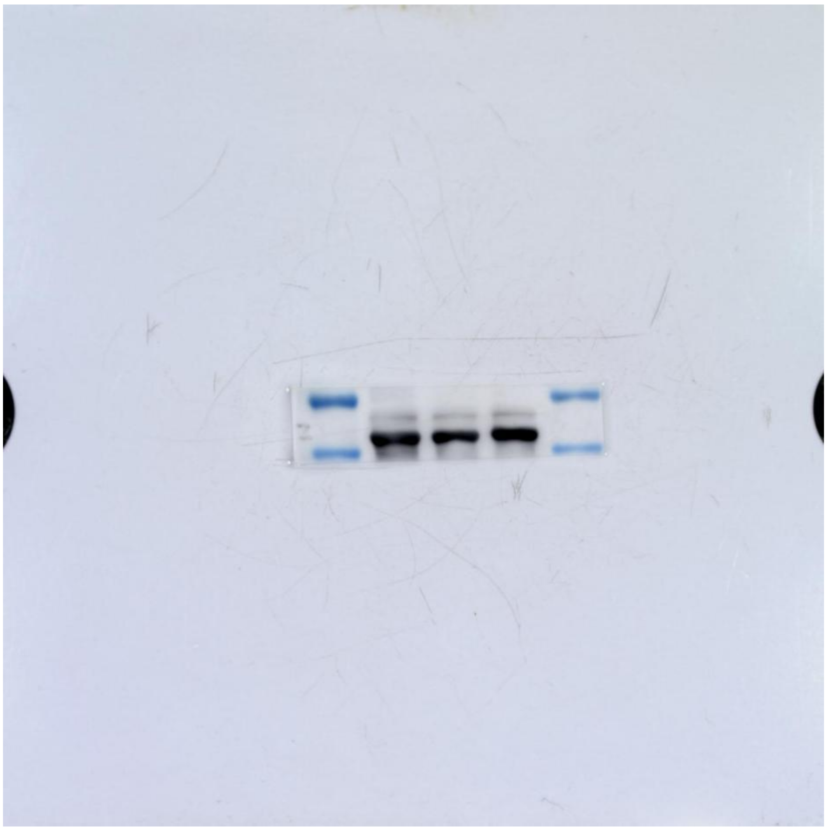

6P FXR1

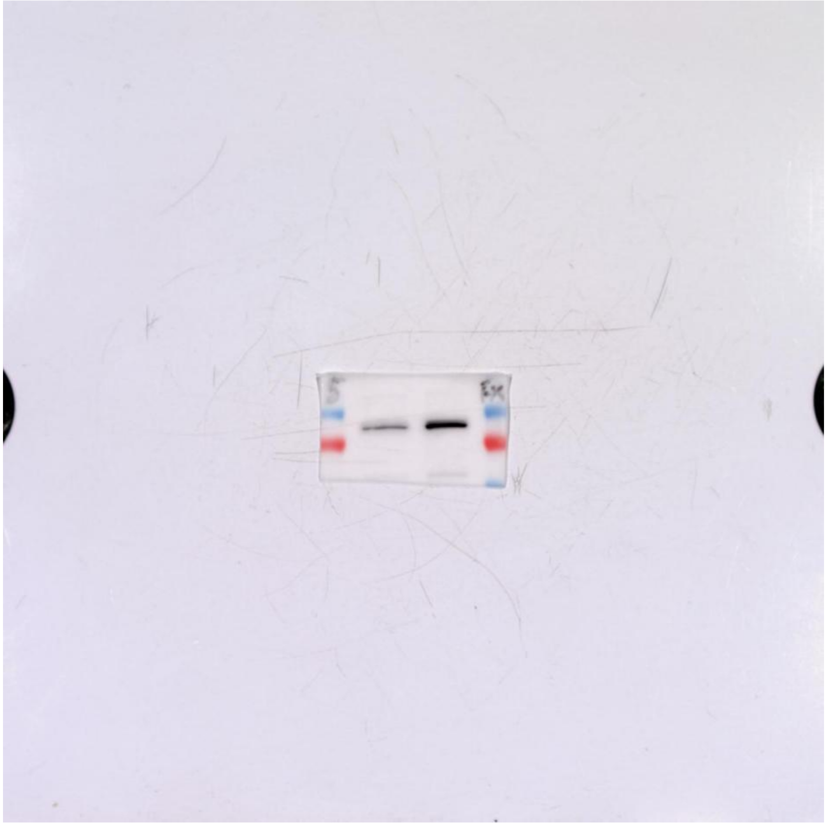

6P OTUD6B

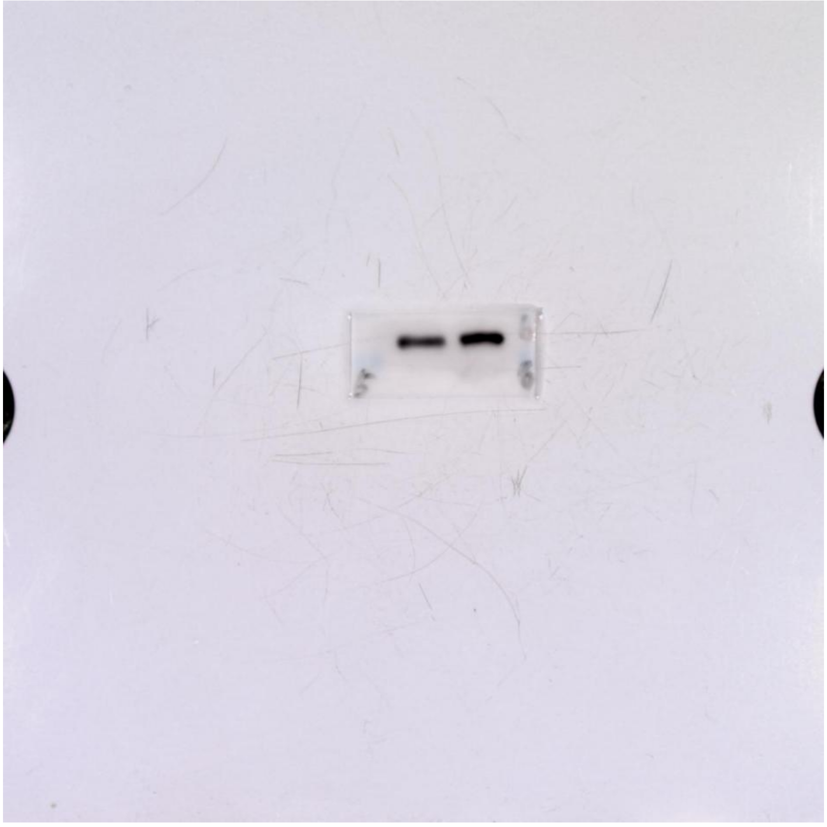

6P β-actin

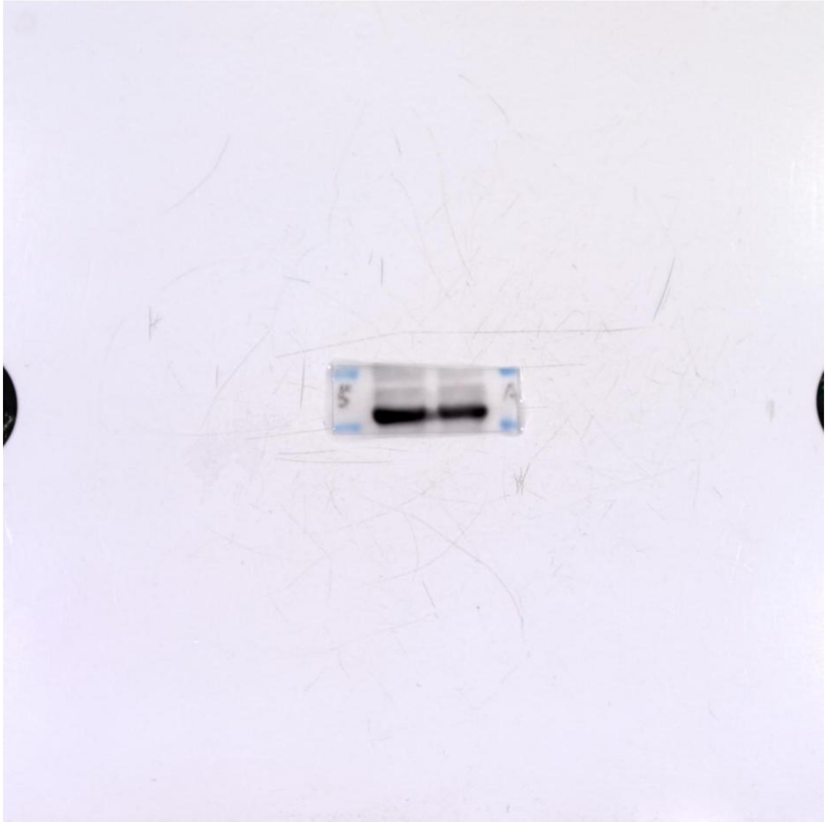

6Q FXR1

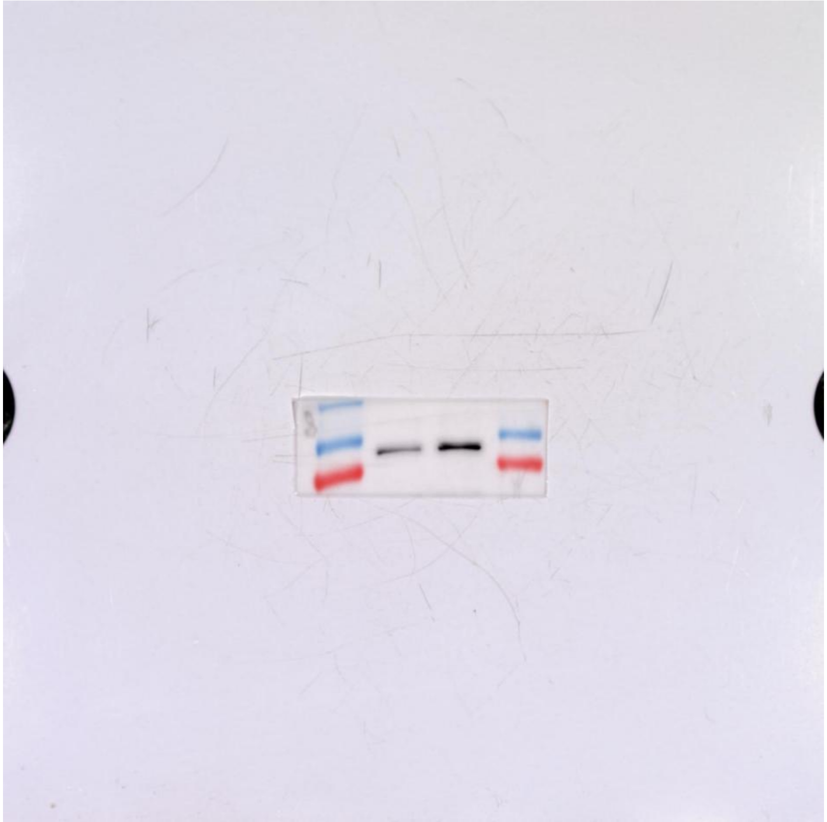

6Q OTUD6B

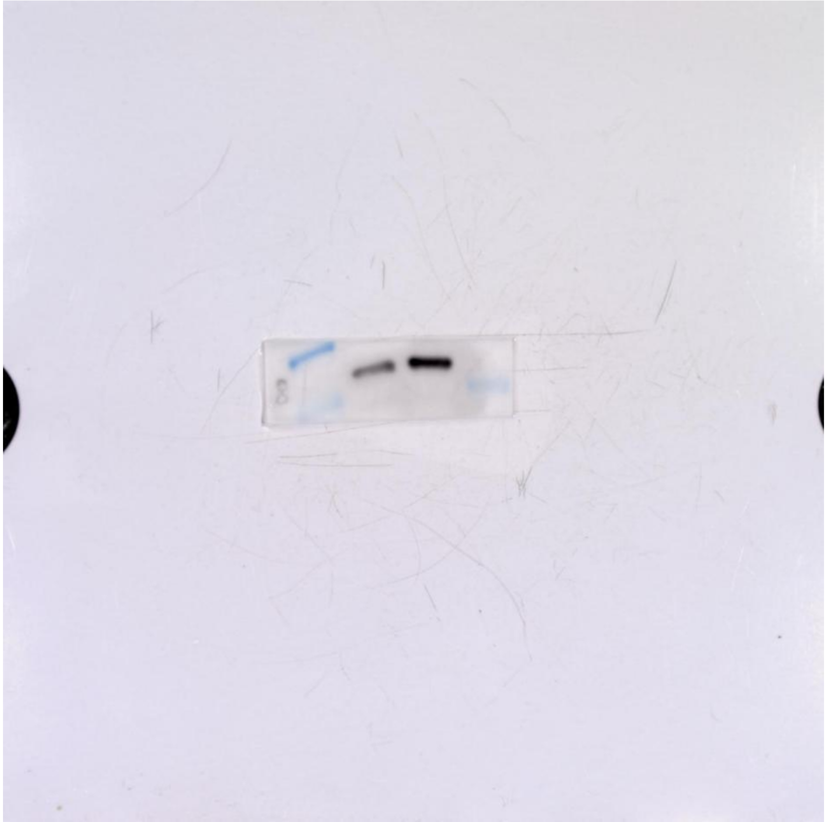

6Q β-actin

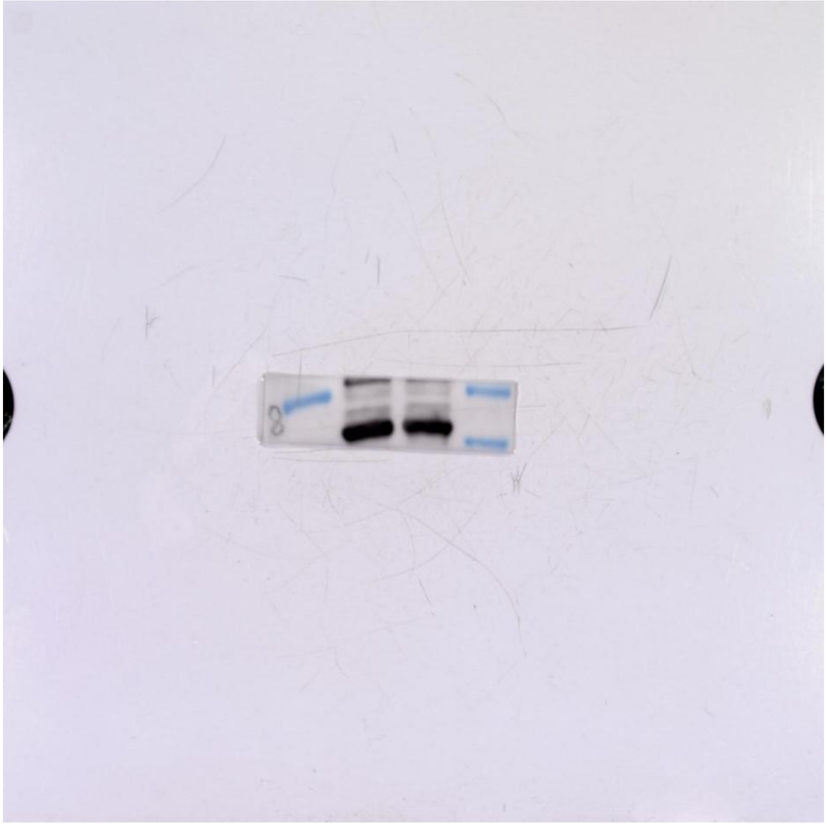

Fig. S2

S2A OTUD6B

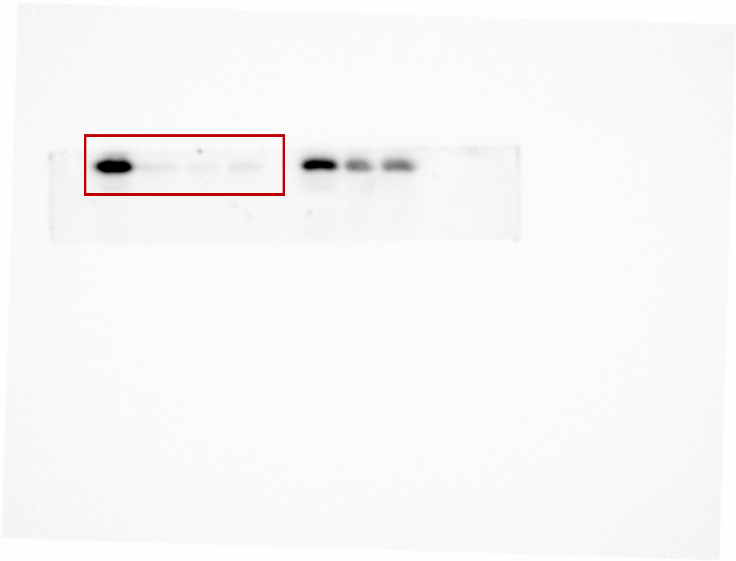

S2A β-actin

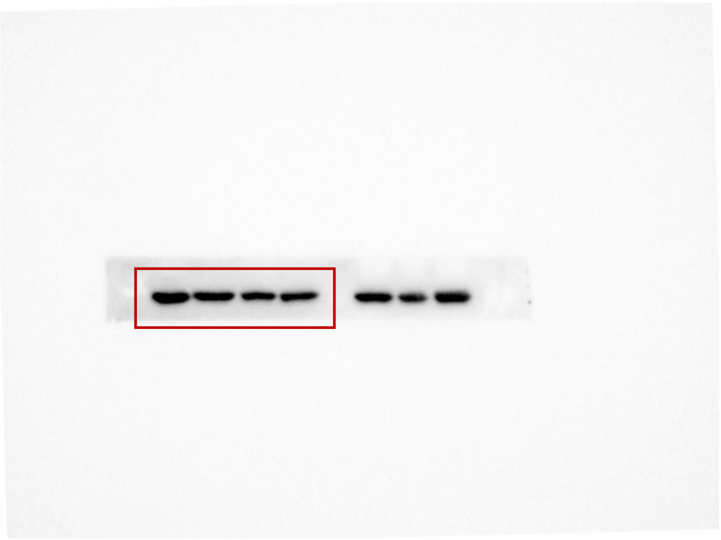

S2D HNRNPU

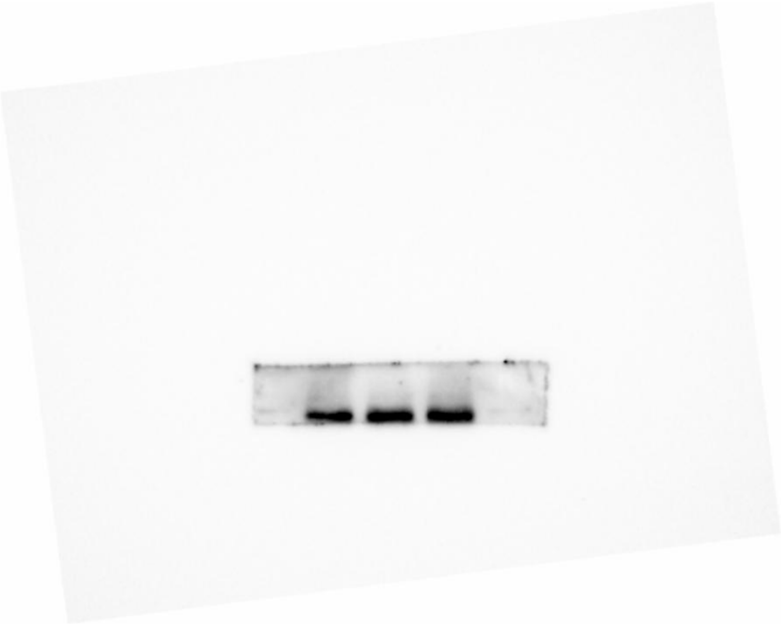

S2D FXR1

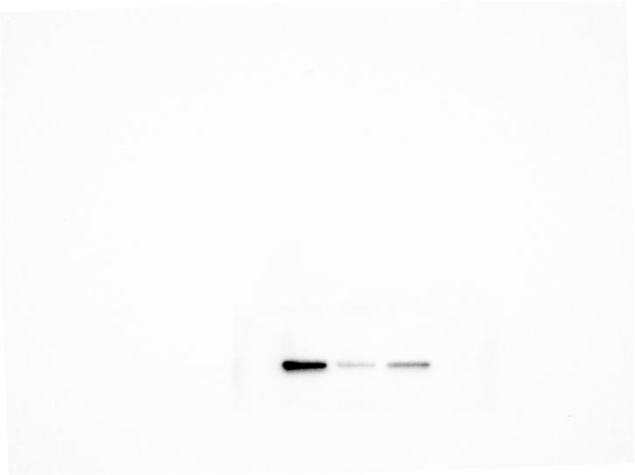

S2D RBM14

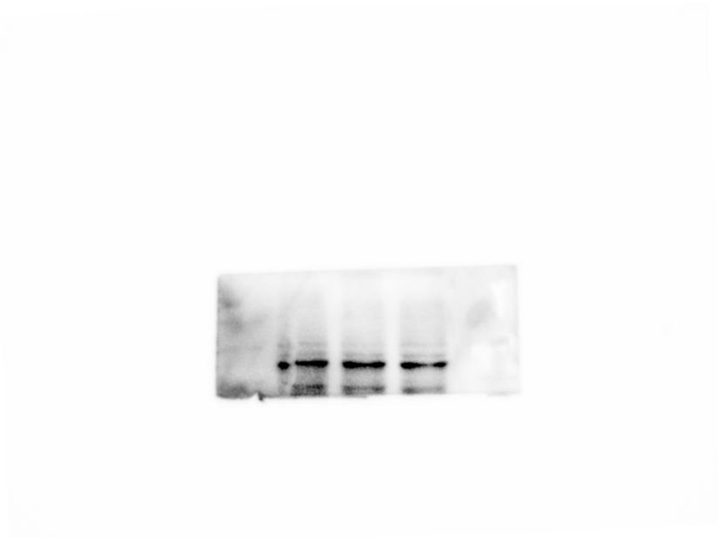

S2D TIA1

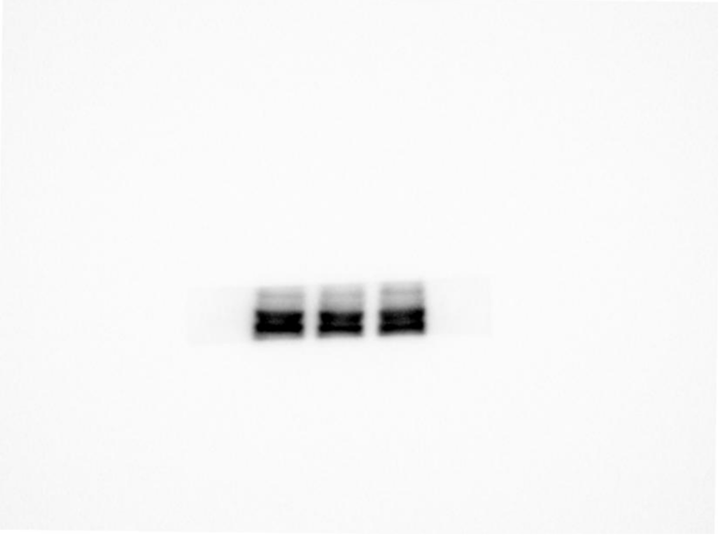

S2D RBM4B

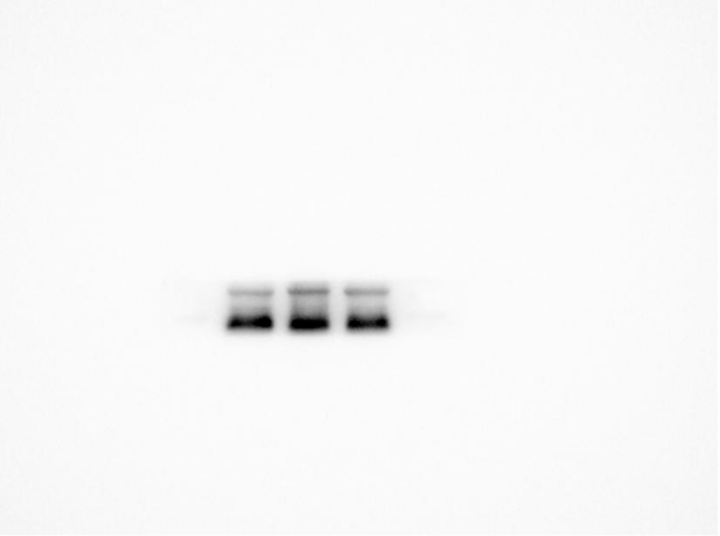

S2D OTUD6B

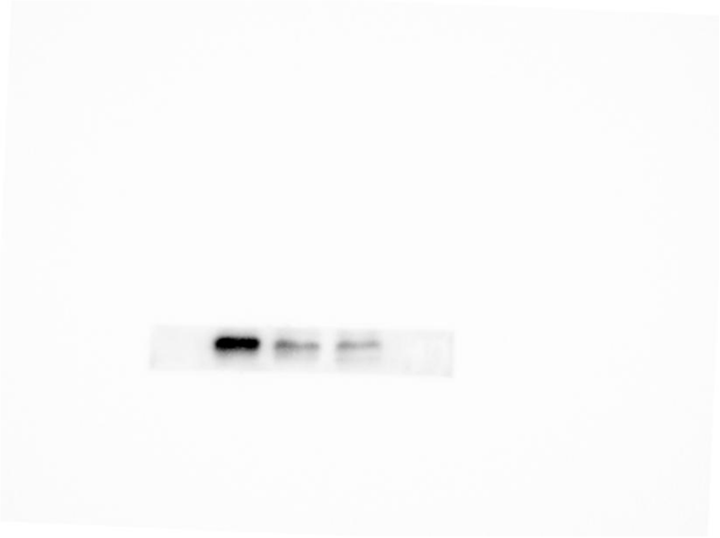

S2D SRSF3

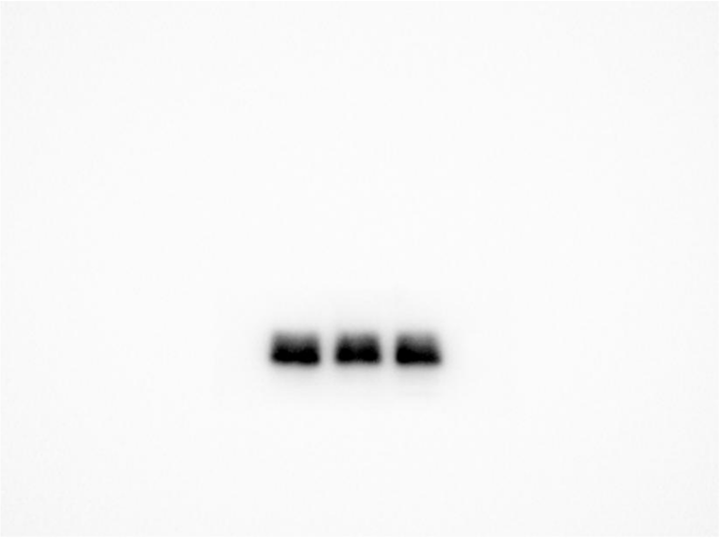

S2D β-actin

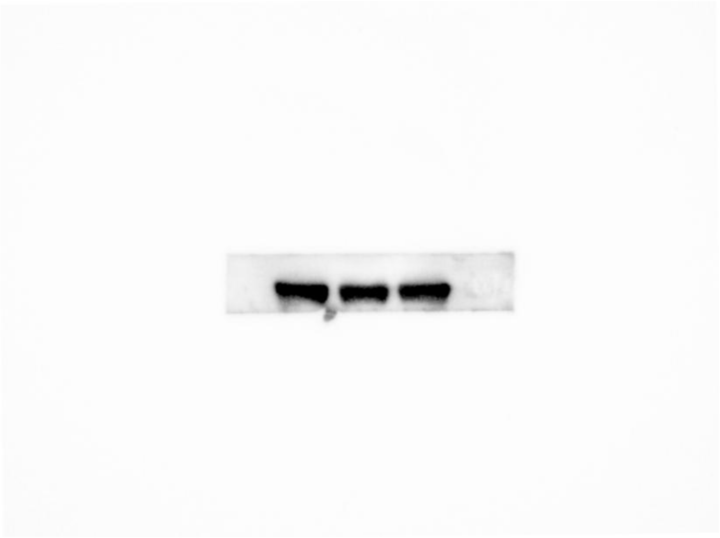

Fig. S4

S4A HA-Ub

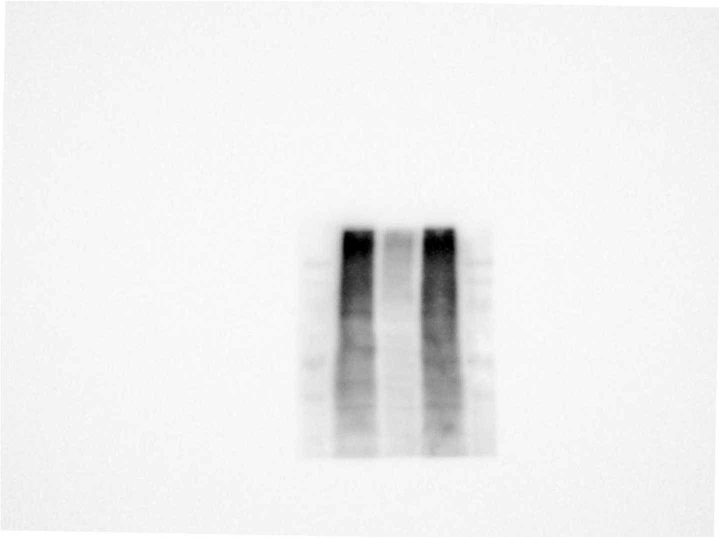

S4A Myc

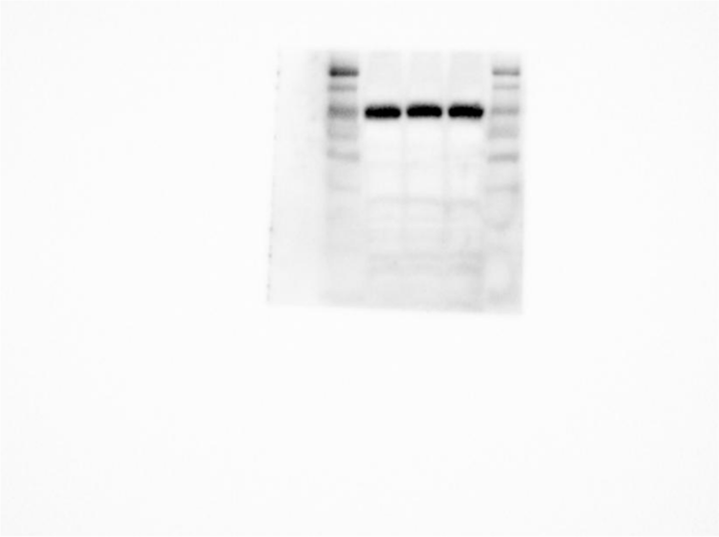

Supplement: Supplementary file 2 — Original Western Blot [file 41419_2026_8812_MOESM2_ESM.pdf]
